# Rational Design, Synthesis, and Biological Evaluation of Third Generation α-Noscapine Analogues as Potent Tubulin Binding Anti-Cancer Agents

**DOI:** 10.1371/journal.pone.0077970

**Published:** 2013-10-21

**Authors:** Naresh Kumar Manchukonda, Pradeep Kumar Naik, Seneha Santoshi, Manu Lopus, Silja Joseph, Balasubramanian Sridhar, Srinivas Kantevari

**Affiliations:** 1 Organic Chemistry Division-II (Crop Protection Chemicals Division), CSIR-Indian Institute of Chemical Technology, Hyderabad, India; 2 Academy of Scientific and Innovative Research, CSIR-Indian Institute of Chemical Technology, Hyderabad, India; 3 X-Ray Crystallography Laboratory, CSIR-Indian Institute of Chemical Technology, Hyderabad-500007, India; 4 Department of Biotechnology and Bioinformatics, Jaypee University of Information Technology, Waknaghat, Distt, Solan, Himachal Pradesh, India; 5 Department of Molecular, Cellular, and Developmental Biology, and the Neuroscience Research Institute, University of California Santa Barbara, Santa Barbara, California, United States of America; 6 UM-DAE Centre for Excellence in Basic Sciences, University of Mumbai campus, Kalina, Santa Cruz (E), Mumbai, India; Bioinformatics Institute, Singapore

## Abstract

Systematic screening based on structural similarity of drugs such as colchicine and podophyllotoxin led to identification of noscapine, a microtubule-targeted agent that attenuates the dynamic instability of microtubules without affecting the total polymer mass of microtubules. We report a new generation of noscapine derivatives as potential tubulin binding anti-cancer agents. Molecular modeling experiments of these derivatives **5a, 6a-j** yielded better docking score (-7.252 to -5.402 kCal/mol) than the parent compound, noscapine (-5.505 kCal/mol) and its existing derivatives (-5.563 to -6.412 kCal/mol). Free energy (Δ*G*_*bind*_) calculations based on the linear interaction energy (LIE) empirical equation utilizing Surface Generalized Born (SGB) continuum solvent model predicted the tubulin-binding affinities for the derivatives **5a, 6a-j** (ranging from -4.923 to -6.189 kCal/mol). Compound **6f** showed highest binding affinity to tubulin (-6.189 kCal/mol). The experimental evaluation of these compounds corroborated with theoretical studies. N-(3-brormobenzyl) noscapine (6f) binds tubulin with highest binding affinity (K_D_, 38 ± 4.0 µM), which is ~ 4.0 times higher than that of the parent compound, noscapine (K_D_, 144 ± 1.0 µM) and is also more potent than that of the first generation clinical candidate EM011, 9-bromonoscapine (K_D_, 54 ± 9.1 µM). All these compounds exhibited substantial cytotoxicity toward cancer cells, with IC_50_ values ranging from 6.7 µM to 72.9 µM; compound **6f** showed prominent anti-cancer efficacy with IC_50_ values ranging from 6.7 µM to 26.9 µM in cancer cells of different tissues of origin. These compounds perturbed DNA synthesis, delayed the cell cycle progression at G2/M phase, and induced apoptotic cell death in cancer cells. Collectively, the study reported here identified potent, third generation noscapinoids as new anti-cancer agents.

## Introduction

**Figure 1:**
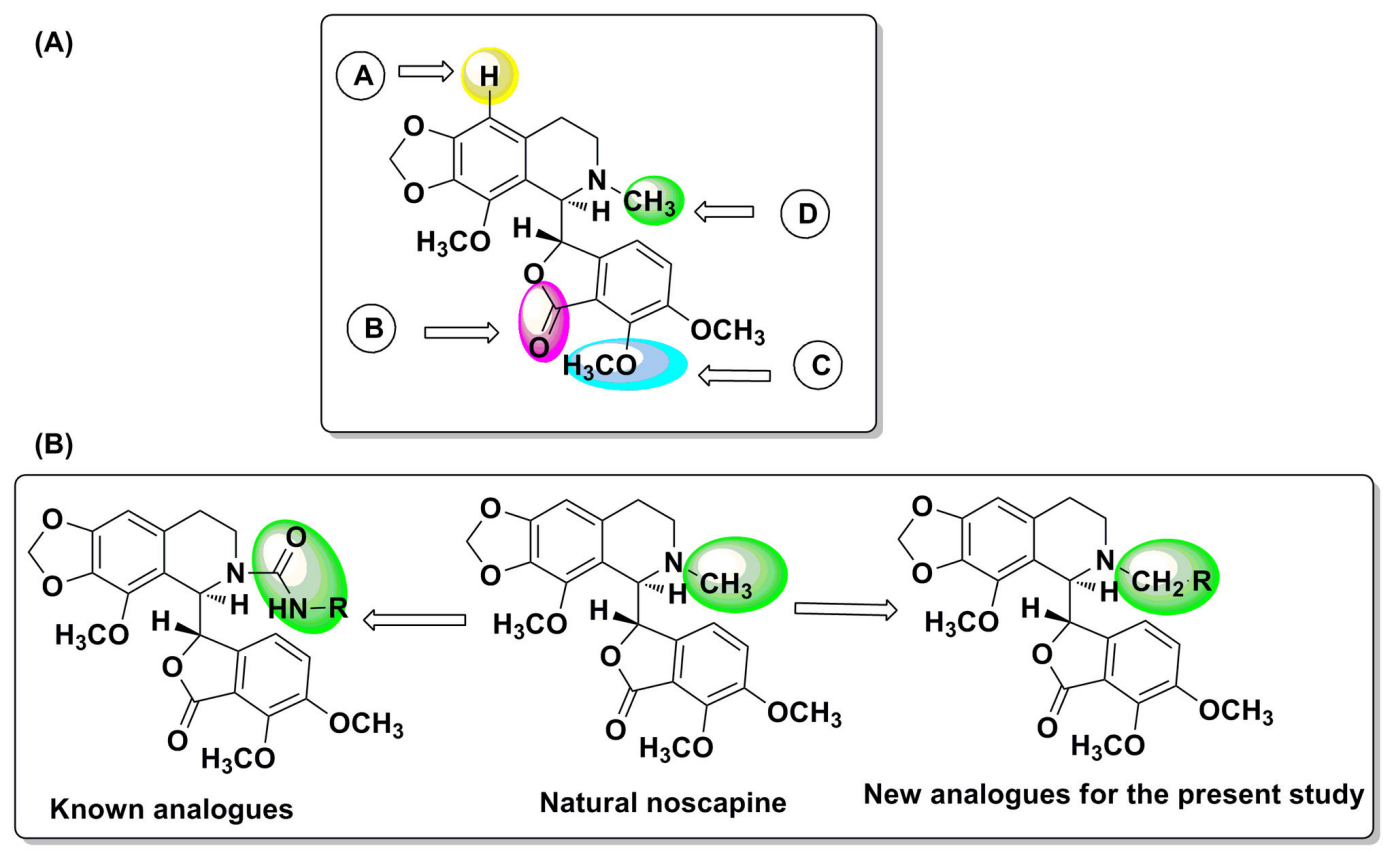
Noscapine scaffold and site of modification. (A) Various diversity points for derivatization of α-noscapine and (B) design strategy for new α-noscapine analogues (basic skeleton and stereochemistry is same as in the natural α-noscapine).

**Figure 2:**
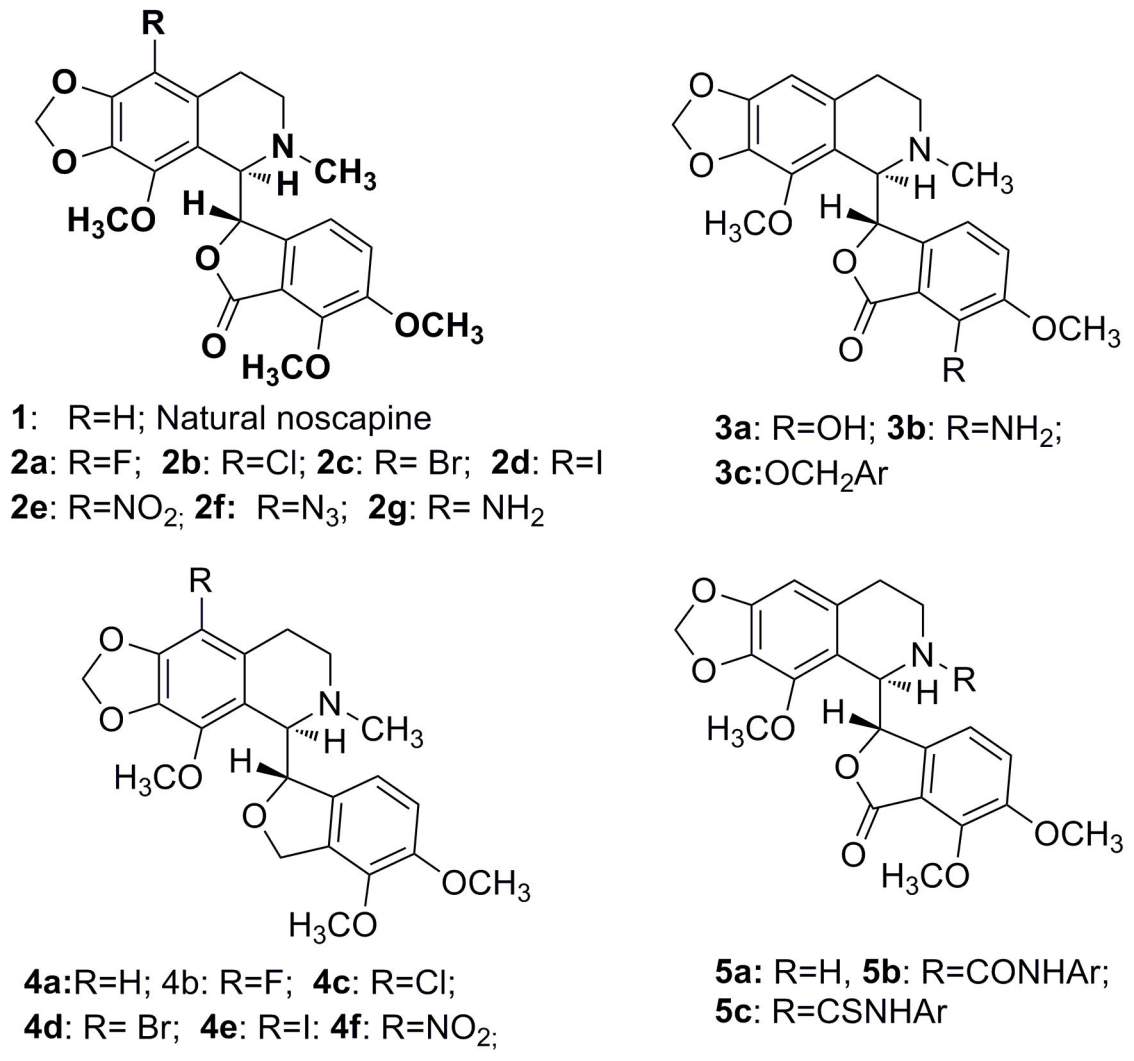
Chemical structure of noscapine and its congeners.

**Figure 3:**
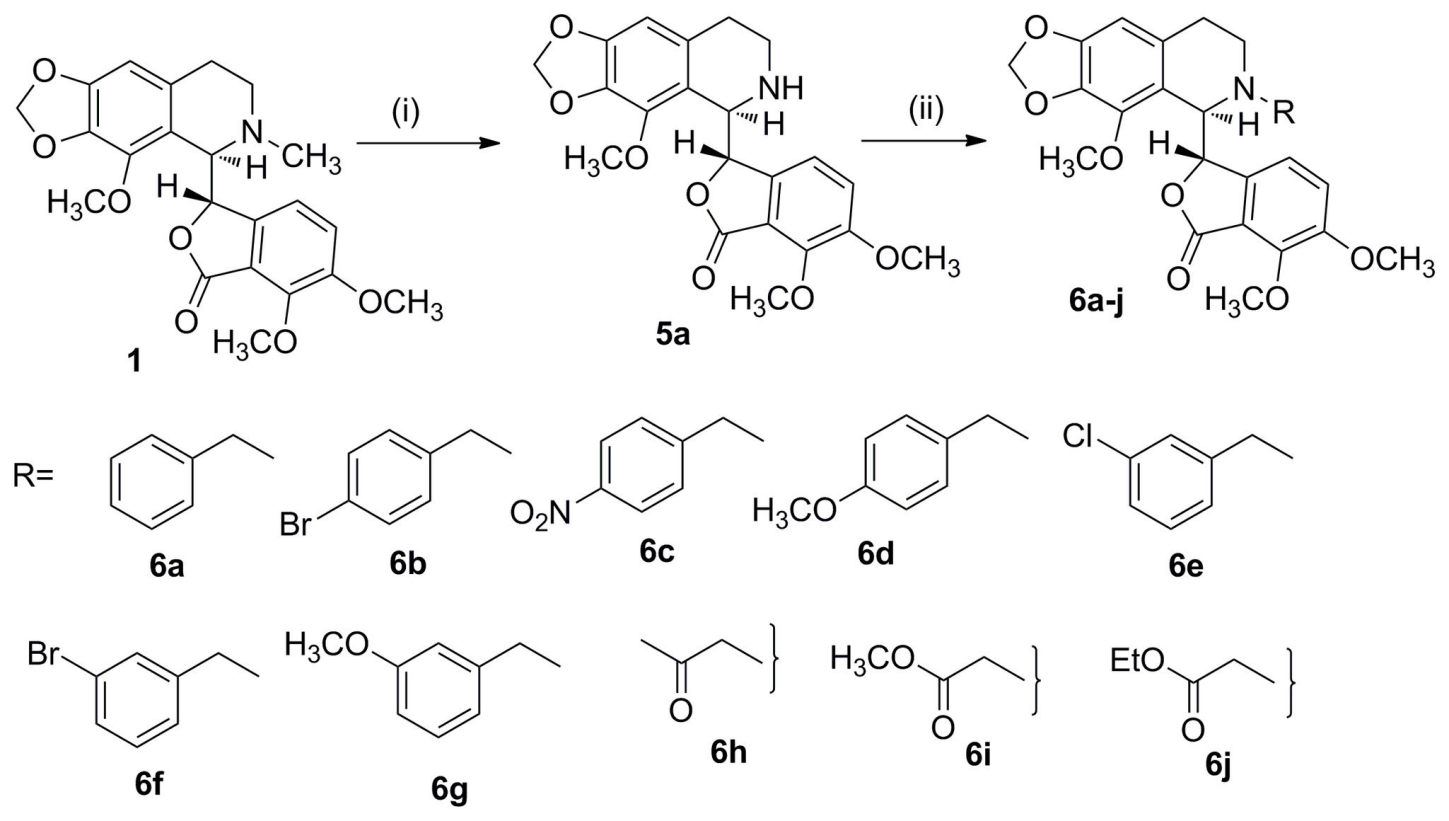
Synthesis of nornoscapine 5a and noscapinoids 6a-j from α-noscapine. Reaction conditions: (i) a: *m*CPBA, DCM; b: 2N HCl; c: FeSO_4_.7H_2_O; (ii) R-Br, KI, K_2_CO_3_, Acetone.

Unlike the current tubulin binding chemotherapeutic drugs such as Paclitaxel and Vinca alkaloids that are confounded by complications with undesirable side effects such as systemic toxicity, noscapine [1-3], an over-the-counter antitussive alkaloid [4,5], is endowed with better anti-cancer profile [6-8] and safer toxicity profile [9-11]. Mechanistically, noscapine binds tubulin with a stoichiometry of one (0.95 ± 0.02) noscapine molecule per tubulin dimer, alters tubulin conformation upon binding [12] yet allows the polymerization of tubulin into microtubules (MTs) [13,14]. Noscapine, however, induces minor suppression of the dynamic instability of microtubules [13,14]. As a result, noscapine blocks mitosis at prometaphase, and perhaps due to the compromised checkpoints, cancer cells selectively get committed to apoptotic cell death leaving normal cells unharmed [6-14]. From a pharmacological perspective, noscapine has many advantages as a microtubule-binding agent [1]. It is effective against multidrug-resistant cancer cell lines, affects cancer cells differently from the normal dividing cells [15,16], has better pharmacokinetic profile [17,18] and does not damage normal tissues (thus devoid of toxic side effects) [19,20]. Even though noscapine has been found to be cytotoxic against a broad range of cancer cells in the public library of the National Cancer Institute, USA (NCI 60-cell screen), the IC_50_ values remains in the high micro molar ranges (~21.1 to 100 μM) [12]. As a result, various noscapine-based tubulin-targeted agents have been developed by modifications mostly at A, B and C sites (Figure 1A) in the noscapine scaffold [21-28]. These compounds are referred to as noscapinoids (Figure 2). The first-generation analogues synthesized by the chemical manipulation at diversity point A on the isoquinoline ring system of noscapine (Figure 1A) included nitro [21], azido [22], amino [23,24] and halogenated [25-27] (fluoro, chloro, bromo, and iodo) α-noscapine analogues, and they displayed superior anti-cancer activity. Based on these insights, the lactone ring of benzofuranone (diversity point B, Figure 1A) was reduced to its cyclic ether analogue [28] (for example **4a**) and examined for its potency as tubulin binding agents [29]. Further diversification at point C (Figure 1A) on the benzofuranone ring system of noscapine has been reported to give the second generation O-alkylated [30]/acylated [31] noscapinoids, including the hydroxy derivative which is more potent than the parent noscapine. These reports suggest that chemical maneuvering of embodied functional groups of noscapine has significant impact on its biological activity. In continuation of our efforts on the design of new noscapine derivatives, we decided to introduce modification at diversity point D (Figure 1B) by functionalization of ‘N’ in isoquinoline unit of natural α-noscapine (we call them third generation α-noscapine analogues) that are likely to enhance the biological activity. The reports described on functionalization at ‘N’ are through urea type linkages [32] and much has not been explored for its biological efficacy. We believe that urea type linkage may not be the correct option as it will disturb the electron density at isoquinoline N through delocalization. Therefore in the present study, we have adopted to introduce functional groups keeping the electronic environment on N mostly intact (i.e. to keep the effect of methyl group intact). Hence, “H” on N-CH_3_ is the target for modification. All the derivatives described and synthesized are in line with this design strategy (Figure 3).

Here we report the third generation noscapine congeners **6a-j** that differ in the substituent coupled to isoquinoline ‘N’ of natural α-noscapine. *In silico* molecular modeling calculations of these analogues with tubulin complex were employed to investigate their binding affinity based on reasonable predictive model. The new analogues, nornoscapine **5a** and **6a-j** were chemically synthesized and examined for their tubulin binding properties, and for their effects on cell cycle progression and anti-proliferative activity in rapidly dividing cancer cells using representative human cancer cell lines of lung, myeloma, breast and cervix. 

## Materials and Methods

### A: Computational methodology

#### Ligand preparation

Molecular structures of novel derivatives of noscapine **5a, 6a-j** (Figure 3) along with the reported noscapinoids **1**, **2a-f** (Figure 2) were built using molecular builder of Maestro (version 9.2, Schrödinger). All these structures were energy minimized using Macromodel (version 9.9, Schrödinger) and OPLS 2005 force field with PRCG algorithm (1000 steps of minimization and energy gradient of 0.001). Appropriate bond order for each structure was assigned using Ligprep (version 2.5, Schrödinger). Complete geometrical optimization of these structures was carried out using hybrid density functional theory with Becke’s three-parameter exchange potential and the Lee-Yang-Parr correlation functional (B3LYP) [33,34] using basis set 3-21G* [35-37]. Jaguar (version 7.7, Schrödinger, LLC) was used for the geometrical optimization of the ligands. 

#### Protein preparation

The co-crystallized colchicine-tubulin complex structure (PDB ID: 1SA0, resolution 3.58Å) [38] was used for molecular docking and rescoring. Multi-step Schrödinger’s protein preparation wizard (PPrep) was used for the final preparation of protein. Missing hydrogen atoms were added to the structure using Maestro interface (version 9.2, Schrödinger). All the water molecules were removed from the complex and optimized the hydrogen bond network using PPrep wizard. The missing amino acids from 37 to 47 (A-chain) and 275 to 284 (B-chain) in the co-crystallized structure were filled using homology-based modeling technique based on different templates such as PDB ID: 3DU7 (C-chain) and PDB ID: 3RYC (D-chain) respectively, using Prime (version 3.0, Schrödinger). The structure obtained was energy minimized using OPLS 2005 force field with Polak-Ribiere Conjugate Gradient (PRCG) algorithm. The minimization was stopped either after 5,000 steps or after the energy gradient converged below 0.001 kcal/mol. All atom molecular dynamics (MD) simulation of protein structure in explicit water was carried out using the GROMACS 4.5.4 software [39] and the GROMOS96 force field for a time scale of 10 ns. Three-dimensional periodic boundary conditions were imposed, enclosing the molecule in a dodecahedron solvated with the SPC216 water model provided in the GROMACS package and energy minimized using 1000 steps of steepest descent. The system was neutralized with 32 Na^+^ counter ion and was locally minimized using 100 steps of steepest descent. The electrostatic term was described using the Particle Mesh Ewald algorithm [40]. The LINCS [41] algorithm was used to constrain all bond lengths and cut-off distances for the calculation of the coulombic and van der Waals interactions at 1.0 nm. The system was equilibrated by 100 ps of MD runs with position restraints on the protein to allow the relaxation of the solvent molecules at 300 K and normal pressure. The system was coupled to the external bath by the Berendsen thermostat with a coupling time of 0.1 ps with default setting. The final MD calculations were performed for 10.0 ns under the same conditions with a time step of 2 fs. The overall quality of the model obtained, stereochemical values and non-bonded interactions were tested using PROCHECK [42], ERRAT [43] and VERIFY3D [44]. The PROCHECK results showed 94.8% of backbone angles are in allowed regions with G-factors of - 0.12. Ramachandran plot [45] analysis revealed only 1.6% residues in the disallowed region and 2.3% residues in generously allowed regions. ERRAT is an “overall quality factor” calculator program for non-bonded atomic interactions. The accepted range in ERRAT is 50 and higher scores indicate the precision of the model. In the case of tubulin, the ERRAT score was 88.402 that is within the range of high quality model. Similarly, the VERIFY 3D score of 95.25% indicates a good quality model. 

#### Molecular docking of ligands and calculation of binding free energies

The receptor-grid file was generated at the centroid of the noscapinoid binding site [46] using Glide (version 5.7, Schrödinger). A bounding box of size 12Å x 12Å x 12Å was defined in tubulin and centered on the mass center of binding site in order to confine the mass center of the docked ligand. The larger enclosing box of size 12Å x 12Å x 12Å which occupied all the atoms of the docked poses was also defined. The scale factor of 0.4 for van der Waals radii was applied to atoms of protein with absolute partial charges less than or equal to 0.25. All the ligands were then docked into the binding site using Glide XP (extra precision) and evaluated using a Glide XP_Score_ function [47,48]. Furthermore, the docked complexes of these ligands were energy minimized based on hybrid Monte Carlo simulation and their binding free energy (Δ*G*_*bind*_) onto tubulin was predicted using linear interaction energy method (LIE) with a surface generalized Born (SGB) continuum solvation model. The LIE-SGB model estimates the binding affinities for a set of novel compounds utilizing the experimental binding affinity data of a set of training set. In this study we have used the original formulation of SGB–LIE (equation 1) proposed by Jorgensen [49] and implemented in Liaison package (version 5.6, Schrödinger, LLC) using the OPLS-2005 force field.

$$\Delta G_{bind}=\alpha \left(\langle U_{vdw}^{b}\rangle -\langle U_{vdw}^{f}\rangle \right)+\beta \left(\langle U_{elec}^{b}\rangle -\langle U_{elec}^{f}\rangle \right)+\gamma \left(\langle U_{cav}^{b}\rangle -\langle U_{cav}^{f}\rangle \right)$$ (1)

Here ⟨ ⟩ represent the ensemble average, *b* represents the bound form of the ligand, *f* represents the free form of the ligand, and α, β, and γ are the coefficients. *U*_*vdw*_, *U*_*elec*_, and *U*_*cav*_ are the van der Waals, electrostatic, and cavity energy terms in the SGB continuum solvent model. The cavity energy term, *U*_*cav*_, is proportional to the exposed surface area of the ligand. Various energy parameters included in equation **1** were calculated from the docked complex corresponding to each analogue using Liaison package as described previously [24]. The average LIE energy terms were used for building the binding affinity model and estimation of binding free energies of noscapine derivatives. The α*,* β, and γ LIE fitting parameters were determined using Minitab statistical package (version 16.0, Minitab Inc.) by fitting the experimental binding affinities of training set molecules. A dataset consisting of 7 noscapine derivatives (compounds: **1, 2a-f**; Figure 2) with known experimental binding affinities was used as a training set. 

### B: Experimental methodology

#### Chemical synthesis of noscapine derivatives

Reagents and all solvents were analytically pure and were used without further purification. All reactions were carried out in oven-dried flasks with magnetic stirring. All the experiments were monitored by analytical thin layer chromatography (TLC) performed on silica gel GF254 pre-coated plates. After elution, the plates were visualized under UV illumination at 254 nm for UV active materials. Staining with PMA and charring on a hot plate achieved further visualization. Solvents were removed *in vacuo* and heated on a water bath at 35 °C. Silica gel finer than 200 mesh was used for column chromatography. Columns were packed as slurry of silica gel in hexane and equilibrated with the appropriate solvent/solvent mixture prior to use. The compounds were loaded neat or as a concentrated solution using the appropriate solvent system. Applying pressure with an air pump assisted the elution. Yields refer to chromatographically and spectroscopically homogeneous materials unless otherwise stated. Appropriate names for all the new compounds were given with the help of ChemBioOffice 2010. Melting points were measured with a Fischer-Johns melting point apparatus and are uncorrected. Purity of all the compounds (> 96%) used for biological screening were determined by analytical HPLC (SPD-M20A, make: Shimadzu) using ODS column eluted with gradient mixture of acetonitrile-water. Infrared spectroscopy (IR) spectra were recorded as neat liquids or KBr pellets and absorptions are reported in cm^-1^. Nuclear magnetic resonance (NMR) spectra were recorded on 300 (Bruker) and 500 MHz (Varian) spectrometers in appropriate solvents using tetramethylsilane (TMS) as an internal standard or the solvent signals as secondary standards and the chemical shifts are shown in δ scales. Multiplicities of NMR signals are designated as s (singlet), d (doublet), t (triplet), q (quartet), br (broad), m (multiplet, for unresolved lines), etc. ^13^C NMR spectra were recorded on 75 MHz spectrometer. High-resolution mass spectra (HRMS) were obtained by using ESI-QTOF mass spectrometry. Optical rotations were measured with a Roudolph Digipol 781 Polarimeter at 25 °C. Commercially available solvents hexane, CH_2_Cl_2_, and EtOAc were used as such without further purification. Natural α-noscapine was purchased from Sigma-Aldrich and is used as such. The synthetic approach for the preparation of noscapine derivatives, **6a-j** is depicted in Figure 3. All these derivatives were synthesized from nornoscapine **5a** as starting material, which in turn was synthesized from noscapine.

**(S)-6,7-dimethoxy-3-((R)-4-methoxy-5,6,7,8-tetrahydro[1,3]dioxolo[4,5-*g*]isoquinolin-5-yl)isobenzofuran-1(3H)-one (5a):** To a solution of natural α- noscapine (2.0 g, 4.84 mmol) in dichloromethane (15 mL) was added mCPBA (1.66 g, 9.7 mmol) portion wise at 0 °C. The reaction mixture was stirred for 1h at room temperature, diluted with dichloromethane (20 mL), excess peroxide was quenched with 1M aq. solution NaHSO_3_ (15 mL), the organic layer was then separated, dried with anhydrous Na_2_SO_4_, and concentrated. The crude residue was dissolved in methanol (20 mL), acidified to pH 1.0 using 2N HCl, stirred for 5 min and filtered. The filtrate was concentrated under reduced pressure, re-dissolved in dichloromethane (20 mL), dried with anhydrous Na_2_SO_4_, filtered and concentrated. The pale yellow solid α- noscapine N-oxide.HCl salt thus obtained was dissolved in methanol (20 mL), FeSO_4_.7H_2_O (2.69 g, 9.68 mmol) was added. After stirring the mixture at room temperature for 12 h, the reaction mixture was concentrated and treated with 25% aqueous ammonia to get pH 10, extracted with dichloromethane (3 x 10 mL), dried with anhydrous Na_2_SO_4_, and evaporated under reduced pressure. The crude residue was subjected to triethyl amine treated silica gel column chromatography and eluted with 3:7 Ethyl acetate: Hexane (2:3) gave(S)-6,7-dimethoxy-3-((*R*)-4-methoxy-5,6,7,8-tetrahydro[1,3]dioxolo[4,5-*g*]isoquinolin-5-yl)isobenzofuran-1(3H)-one (5a) (0.92 g, 48%) as white solid. mp 170 °C; [α]_D_^25^ = -105.6 (c=1, Methanol), Yield: 48% IR ν_max_ (cm^-1^): 3360, 2942, 1759, 1624, 1501, 1280, 1119, 1074, 1042, 1023, 932, 796, 679 ^1^.HNMR (300 MHz, CDCl_3_): δ 6.94(d, J=8.30 Hz, 1H), 6.33(s,1H), 5.99-5.89(m,4H), 4.85(d, J = 4.53 Hz, 1H), 4.09(s,3H), 4.07(s,3H), 3.85(s, 3H), 2.69-2.58(m, 1H), 2.54-2.42(m,1H), 2.36-2.23(m,1H), 2.22-2.09(m, 1H) ^13^.CNMR (75 MHz, CDCl_3_) δ 168.5, 152.1, 148.3,147.8, 141.0, 140.4, 134.1, 131.9, 119.6, 118.3, 117.5, 116.9, 103.1, 100.7, 20.6, 62.2, 59.4, 56.6, 52.7, 39.5, 29.7 MS (ESI) *m*/*z* 400 [M+H]^+^; HRMS (ESI) Calcd for C_21_H_22_NO_7_: 400.1396, found: 400.1401.

#### General procedure for the preparation of 6a-j

To the solution of (*S*)-6,7-dimethoxy-3-((*R*)-4-methoxy-5,6,7,8-tetrahydro[1,3]dioxolo[4,5-*g*]isoquinolin-5-yl)isobenzofuran-1(3H)-one **5a** (200 mg, 0.50 mmol) in acetone (5 mL), was added potassium carbonate (1.10 mmol), potassium iodide (0.5 mmol) and alkyl bromide (0.55 mmol) and stirred at room temperature (RT) for 1 h. Crude reaction mixture was filtered, the filtrate was evaporated under vacuum, water (5 mL) and dichloromethane (2 X 10 mL) was added, organic layer was separated, washed with H_2_O, dried over anhydrous Na_2_SO_4_ and filtered. The residue thus obtained was chromatographed over triethyl amine treated silica gel column eluting with hexane / ethyl acetate (70:30) to yield **6a-j** as solid products.

**(S)-3-((*R*)-6-benzyl-4-methoxy-5,6,7,8-tetrahydro-[1,3] dioxolo[4,5-*g*] isoquinolin-5-yl)-6,7-dimethoxyisobenzofuran-1(3H)-one(6a):** Yield: 93%; mp 64°C; [α]_D_^25^ = 1.3 (c = 1, Dichloromethane); IR ν_max_ (cm^-1^): 3503, 2490, 2837, 1759, 1621, 1595, 1569, 1498, 1271, 1212, 1039, 891, 785, 695 ^1^.HNMR (300 MHz, CDCl_3_) 7.33-7.15(m, 5H), 6.98(d, J=8.30 Hz, 1H), 6.33(s, 1H), 6.17(d, J=8.30 Hz, 1H), 5.94(s, 2H), 5.66(d, J=3.96 Hz, 1H), 4.62(d, J=3.96 Hz, 1H), 4.15-4.06(m, 4H), 4.02(s, 3H), 3.86(s, 3H), 3.66(d, J=13.21 Hz, 1H), 2.51-2.36(m, 2H), 2.35-2.15 (m, 1H), 2.06-1.91(m, 1H) ^13^.CNMR (75 MHz, CDCl_3_) δ 168.1, 152.2, 148.4, 147.8, 141.3, 140.5, 139.0, 133.9, 131.9, 128.7, 126.9, 119.8, 118.2, 117.7, 116.9, 102.4, 100.7, 81.7, 61.5, 59.6, 56.7, 59.6, 62.3, 45.1, 26.5. MS (ESI) *m*/*z* 490 [M+H]^+^

**5(S)-3-((R)-6-(4-bromobenzyl)-4-methoxy-5,6,7,8-tetrahydro-[1,3]dioxolo[4,5-g]isoquinolin-5-yl)-6,7-dimethoxyisobenzofuran-1(3H)-one (6b):** Yield: 95%; mp 76 °C; [α]_D_^25^ = -146.0 (c = 1, Dichloromethane); IR ν_max_ (cm^-1^): 3493, 2939, 2837, 1759, 1622, 1596, 1497, 1479, 1271, 1213, 1115, 1079, 971, 789, 711, 644, 479, 811, 746 ^1^.HNMR (300 MHz, CDCl_3_) δ 7.37(d, J = 8.30 Hz, 2H), 7.12(d, J = 8.30 Hz, 2H), 6.92(d, J = 8.30 Hz, 1H), 6.28(s, 1H), 6.04(bs, 1H), 5.94(s, 2H), 5.59(bs, 1H), 4.57(d, J = 3.77 Hz, 1H,), 4.14-3.89(m, 7H), 3.85(s, 3H), 3.57(d, 1H), 2.46-2.09(m, 3H), 2.00-1.84(m, 1H) ^13^.CNMR (75 MHz, CDCl_3_) δ 168.1, 152.2, 148.5, 147.8, 141.0, 140.4, 138.0, 133.9, 131.8, 131.2, 130.4, 120.6, 119.8, 118.1, 117.7, 116.6, 102.4, 100.7, 81.5, 62.3, 61.0, 59.4, 59.3, 56.7, 45.3, 26.6.MS (ESI) *m*/*z* 568 [M+H]^+^. HRMS (ESI) Calcd for C_28_H_27_NO_7_: 568.0970, found: 568.0946.

**(S)-6,7-dimethoxy-3-((R)-4-methoxy-6-(4-nitrobenzyl)-5,6,7,8-tetrahydro-[1,3]dioxolo[4,5-g]isoquinolin-5-yl)isobenzofuran-1(3H)-one (6c):** Yield: 94%; mp 154 °C; [α]_D_^25^ = 68.0 (c = 1, Dichloromethane ); IR ν_max_ (cm^-1^): 3490, 3078, 2931, 2901, 2837, 1751, 1620, 1520, 1499, 1389, 1343, 1274, 1213, 1080, 972, 852, 733, 610 ^1^.HNMR (300 MHz, CDCl_3_) δ 8.13(d, J = 8.49 Hz, 2H),7.40 (d, J = 8.49 Hz, 2H), 6.93(d, J = 8.30Hz, 1H), 6.30(s, 1H), 6.01-5.92(m, 3H), 5.62(d, J = 3.77 Hz, 1H), 4.61(d, J = 3.77Hz, 1H), 4.27(d, J = 14.35 Hz, 1H), 4.10(s, 3H), 4.09(s, 3H), 3.87(s, 3H), 3.74(d, J = 14.35 Hz, 1H), 2.44-2.28(m, 2H), 2.23-2.13(m, 1H), 2.00-1.85(m, 1H) ^13^.CNMR (75 MHz, CDCl_3_) δ 168.1, 152.3, 148.6, 147.8, 147.1, 147.0, 140.6, 140.3, 134.0, 131.7, 129.0, 123.4, 119.9, 118.1, 117.7, 116.3, 102.4, 100.8, 81.4, 62.3, 61.4, 59.5, 59.4, 56.6, 46.0, 27.0. MS (ESI) *m*/*z* 557 [M+H]^+^; HRMS (ESI) Calcd for C_28_H_26_N_2_O_9_Na: 557.1536, found: 557.1557.

**(S)-6,7-dimethoxy-3-((R)-4-methoxy-6-(4-methoxybenzyl)-5,6,7,8-tetrahydro[1,3]dioxolo[4,5-g]isoquinolin-5-yl)isobenzofuran-1(3H)-one (6d):** Yield: 92%; mp 66 °C; [α]_D_^25^ = 6.66 (c = 1, Dichloromethane); IR ν_max_ (cm^-1^): 3492, 2936, 2836, 1759, 1613, 1511, 1269, 1115, 1013, 970, 821, 713, 517 cm^-1 1^.HNMR (300 MHz, CDCl_3_) 7.15(d, J = 8.49 Hz, 2H), 6.92(d, J = 8.12 Hz, 1H), 6.76(d, J = 8.49 Hz, 2H), 6.28(s, 1H), 6.12(d, J = 8.12Hz, 1H), 5.93(s, 2H), 5.58(d, J = 3.58 Hz, 1H), 4.55(d, J = 3.55 Hz, 1H), 4.10(s, 3H), 4.03(m, 4H), 3.85(s, 3H), 3.76(s, 3H), 3.55(d, J = 12.84 Hz, 1H), 2.50-2.13(m, 3H), 2.04-1.83(m, 1H) ^13^.CNMR (75 MHz, CDCl_3_) δ 168.1, 158.5, 152.1, 148.4, 147.7, 141.2, 140.5, 133.9, 131.7, 130.0, 119.7, 118.1, 117.8, 113.4, 116.6, 102.4, 100.6, 81.5, 62.3, 60.7, 59.3, 59.2, 56.7, 55.1, 44.8, 26.2.MS (ESI) *m*/*z* 520 [M+H]^+^; HRMS (ESI) Calcd for C_29_H_29_ NO_8_Na: 542.1790, found: 542.1817.

**(S)-3-((R)-6-(3-chlorobenzyl)-4-methoxy-5,6,7,8-tetrahydro-[1,3]dioxolo[4,5-g]isoquinolin-5-yl)-6,7-dimethoxyisobenzofuran-1(3H)-one (6e):** Yield: 92%; mp 62 °C; [α]_D_^25^ = 36.0 (c = 1, Dichloromethane); IR ν_max_ (cm^-1^): 3395, 3022, 2925, 2849, 1728, 1603, 1486, 1302, 1261, 1186, 1058, 811, 747 ^1^.HNMR (300 MHz, CDCl_3_) δ 7.29-7.12 (m, 4H), 6.99 (d, J = 8.30 Hz, 1H), 6.34 (s, 1H), 6.14 (d, J = 8.30 Hz, 1H), 5.95 (s, 2H), 5.67 (d, J = 3.77 Hz, 1H), 4.61 (d, J = 3.77 Hz, 1H), 4.17-4.08 (m, 4H), 4.05 (s, 3H), 3.87 (s, 3H), 3.63 (d, J = 13.59 Hz, 1H), 2.49-2.37 (m, 2H), 2.31-2.16 (m, 1H), 2.06-1.93 (m, 1H) ^13^.CNMR (75 MHz, CDCl_3_) δ 168.1, 152.2, 148.5, 141.2, 140.9, 140.4, 133.9, 131.8, 129.4, 128.4, 127.0, 126.7, 119.7, 118.1, 117.7, 116.6, 102.3, 100.7, 81.7, 64.3, 62.4, 61.2, 59.5, 59.3, 56.6, 45.5, 26.8. MS (ESI) *m*/*z* 524 [M+H]^+^.

**(S)-3-((*R***)**-6-(3-bromobenzyl**)**-4-methoxy-5,6,7,8-tetrahydro-[1,3**]**dioxolo[4,5-g**]**isoquinolin-5-yl**)**-6,7-dimethoxyisobenzofuran-1**(**3H**)**-one (6f):** Yield: 97%; mp 65 °C; [α]_D_^25^ = 52.0 (c = 1, Dichloromethane); IR ν_max_ (cm^-1^): 3503, 2940, 2837, 1759, 1621, 1498, 1387, 1271, 1212, 1039, 891, 785, 695 ^1^.HNMR: (300 MHz, CDCl_3_) δ 7.40-7.30 (m, 2H),7.24-7.09 (m, 2H), 6.99 (d, J = 8.30 Hz, 1H), 6.34 (s, 1H), 6.15 (d, J = 8.30 Hz, 1H), 5.95 (s, 2H), 5.66 (d, J = 3.96 Hz, 1H), 4.60 (d, J = 3.96 Hz, 1H), 4.17-4.06 (m, 4H), 4.04 (s, 3H) 3.87, (s, 3H), 3.63 (d, J = 13.78 Hz, 1H), 2.50-2.37 (m, 2H), 2.32-2.19 (m, 1H), 2.07-1.92 (m, 1H) ^13^.CNMR (75 MHz, CDCl_3_) 168.1, 152.2, 148.5, 147.9, 141.5, 140.4, 134.0, 131.8, 131.4, 130.0, 129.7, 127.3, 122.2, 118.1, 117.7, 116.6, 102.4, 100.7, 81.6, 81.1, 62.5, 61.1, 59.5, 59.3, 56.7, 45.4, 26.8. MS (ESI) m/z 568 [M+H]^+^.

**(S)-6,7-dimethoxy-3-((R)-4-methoxy-6-(3-methoxybenzyl)-5,6,7,8-tetrahydro-[1,3]dioxolo[4,5-g] isoquinolin-5-yl) isobenzofuran-1(3H)-one (6g):** Yield: 94%; mp 60 °C; [α]_D_^25^ = -144.01 (c = 1, Dichloromethane); IR ν_max_ (cm^-1^): 3501, 2941, 2836, 1760, 1620, 1598, 1497, 1387, 1268, 1212, 1012, 933, 786, 693 ^1^.HNMR (300 MHz, CDCl_3_) δ 7.21-7.11 (t, 1H), 7.00-6.89 (m, 2H), 6.84-6.72 (m, 2H), 6.33 (s, 1H), 6.14 (d, J = 8.30 Hz, 1H), 5.95 (s, 2H), 5.67 (d, 4.53, 1H), 4.62 (d, J = 4.53 Hz, 1H), 4.10 (s, 3H), 4.03 (s, 3H), 3.88-3.80 (m, 7H), 3.63 (d, J = 12.84 Hz, 1H), 2.51-2.33 (m, 2H), 2.33-2.16 (m, 1H), 2.03-1.87 (m, 1H) ^13^.CNMR (75 MHz, CDCl_3_) δ 168.1, 159.6, 152.2, 148.4, 147.8, 141.2, 140.7, 140.5, 133.9, 131.9, 128.8, 120.9, 119.9, 118.2, 117.7, 116.8, 113.6, 113.0, 102.4, 100.7, 81.5, 62.3, 61.6, 59.4, 59.3, 56.7, 55.2, 45.2, 26.4. MS (ESI) m/z 520 [M+H]^+^.

**(S)-6,7-dimethoxy-3-((R)-4-methoxy-6-(2-oxopropyl)-5,6,7,8-tetrahydro-[1,3]dioxolo[4,5-g] isoquinolin-5-yl) isobenzofuran-1(3H)-one (6h):** Yield: 98%; mp 179 °C; [α]_D_^25^ = -34.0 (c = 1, Dichloromethane); IR ν_max_ (cm^-1^): 3393, 2957, 2904, 2840, 1764, 1706, 1624, 1500, 1482, 1387, 1364, 1275, 1223, 1041, 1010, 941, 897, 828, 710, 693, 538.cm^-1 1^.HNMR (300 MHz, CDCl_3_) 6.90 (d, J = 8.30 Hz, 1H), 6.28 (s, 1H), 6.00-5.88 (m, 3H), 5.51 (d, J = 3.58 Hz, 1H), 4.61 (d, J = 3.58 Hz, 1H), 4.08 (s, 3H), 4.01 (s, 3H), 3.85 (s, 3H), 3.64 (d, J = 4.15 Hz , 2H), 2.50-2.39(m, 2H), 2.37-2.29 (m, 1H), 2.09 (s, 3H), 1.91-1.76 (m, 1H) ^13^.CNMR (75 MHz, CDCl_3_) δ 167.7, 153.2, 148.5, 140.3, 140.2, 131.8, 120.2, 118.0, 117.7, 116.8, 102.4, 100.7, 96.0, 81.8, 67.6, 62.1, 59.4, 59.0, 56.6, 47.3, 29.6, 28.2, 27.2. MS (ESI) *m*/*z* 456 [M+H]^+^; HRMS (ESI) Calcd for C_24_H_25_NO_8_Na: 478.1477, found: 478.1470.

**Methyl 2-((R)-5-((S)-4,5-dimethoxy-3-oxo-1,3-dihydroisobenzofuran-1-yl)-4-methoxy-7,8-dihydro-[1,3]dioxolo[4,5-g]isoquinolin-6(5H)-yl)acetate (6i):** Yield: 97%; mp 155 °C; [α]_D_^25^ = -66.6 (c = 1, Dichloromethane); IR ν_max_ (cm^-1^): 3488, 3006, 2947, 2919, 2851, 1755, 1627, 1590, 1484, 1387, 1265, 1214, 1085, 1017, 894, 789, 735, 696, 546 ^1^.HNMR (300 MHz, CDCl_3_) δ 6.96 (d, J = 8.30 Hz, 1H), 6.31 (s, 1H), 6.03 (d, J = 8.30 Hz, 1H), 5.95 (d, J = 3.21 Hz, 2H), 5.52 (d, J = 3.96 Hz, 1H), 4.86 (d, J = 3.96 Hz, 1H), 4.10 (s, 3H), 4.04 (s, 3H), 3.86 (s, 3H), 3.80 (d, J = 15.86 Hz, 1H), 3.65 (s, 3H), 3.54 (d, J = 15.86 Hz, 1H), 2.81-2.53 (m, 2H), 2.40-2.23 (m, 1H), 1.94-1.74 (m, 1H) ^13^.CNMR (75 MHz, CDCl_3_) δ 171.7, 167.8, 152.1, 148.3, 147.5, 140.2, 140.1, 134.2, 131.8, 120.1, 117.9, 117.7, 117.3, 102.3, 100.7, 82.2, 62.1, 59.4, 58.2, 58.1, 56.6, 51.1, 47.3, 28.9.MS (ESI) m/z 494 [M+H]^+^.

**Ethyl 2-((R)-5-((S)-4,5-dimethoxy-3-oxo-1,3-dihydroisobenzofuran-1-yl)-4-methoxy-7,8-dihydro-[1,3]dioxolo[4,5-*g*]isoquinolin-6(5H)-yl)acetate (6j):** Yield: 98%; mp 92 °C; [α]_D_^25^ = -174.68 (c = 1, Dichloromethane); IR ν_max_ (cm^-1^): 3511, 2945, 2921, 2839, 1763, 1727, 1625, 1481, 1389, 1270, 1203, 1111, 1032, 931, 817, 790, 703, 614 ^1^.HNMR (300 MHz, CDCl_3_) δ 6.90 (d, J = 8.12 Hz, 1H), 6.27 (s, 1H), 6.01-5.82 (m, 3H), 5.44 (d, J = 3.96 Hz, 1H), 4.84(d, J = 3.96 Hz, 1H), 4.19-4.00 (m, 8H), 3.85 (s, 3H), 3.83 (d, 1H), 3.49 (d, J = 17.56 Hz, 1H), 2.75-2.54 (m, 2H), 2.38-2.20 (m, 1H), 1.91-1.71 (m, 1H), 1.24 (t, 3H) ^13^.CNMR (75 MHz, CDCl_3_) δ 171.2, 167.7, 152.1, 148.3, 147.5, 140.3, 140.1, 134.2, 131.9, 120.2, 117.8, 117.7, 117.4, 102.3, 100.7, 96.0, 82.2, 62.2, 60.1, 59.4, 58.1, 56.6, 47.3, 29.0, 14.1. MS (ESI) m/z 486 [M+H]^+^; HRMS (ESI) Calcd for C_25_H_27_ NO_9_Na: 508.1583, found:508.1578.

#### X-ray crystallographic analysis

 X-ray data for the compounds 6h and 6i were collected at room temperature using a Bruker Smart Apex CCD diffractometer with graphite monochromated MoKα radiation (λ=0.71073Å) using ω-scan method [50]. Preliminary lattice parameters and orientation matrices were obtained from four sets of frames. Integration and scaling of intensity data was accomplished using SAINT program. The structures of 6h and 6i were solved by direct methods using SHELXS97 and refinement was carried out by full-matrix least-squares technique using SHELXL97 [50]. Anisotropic displacement parameters were included for all non-hydrogen atoms. All H atoms attached to C and N were located in difference Fourier maps and were geometrically optimized and allowed for as riding atoms, with C-H = 0.93-0.97 Å, N-H = 0.86 Å, with U_iso_(H) = 1.5U_eq_(C) for methyl H or 1.2U_eq_(C,N). The methyl groups were allowed to rotate but not to tip.

**Crystal data for 6h**: C_24_H_25_NO_8_, *M* = 455.45, colorless plate, 0.17 x 0.15 x 0.07 mm^3^, orthorhombic, space group *P*2_1_2_1_2_1_ (No. 19), *a* = 8.7173(12), *b* = 12.8144(17), *c* = 19.436(3) Å, *V* = 2171.2(5) Å^3^, *Z* = 4, *D*_c_ = 1.393 g/cm^3^, F_000_ = 960, CCD Area Detector, MoKα radiation, λ = 0.71073 Å, *T* = 294(2) K, 2*θ*_max_ = 50.0°, 21015 reflections collected, 2200 unique (R_int_ = 0.0227). Final GooF = 1.045, *R1* = 0.0279, *wR2* = 0.0774, *R* indices based on 2086 reflections with I>2σ(I) (refinement on F^2^), 302 parameters, 0 restraints, µ** = 0.105 mm^-1^. CCDC 914991 contains supplementary Crystallographic data for the structure. The detailed elucidation of crystal structure and analysis will be published elsewhere.

**Crystal data for 6i**: C_24_H_25_NO_9_, *M* = 471.45, colorless needle, 0.18 0.12 0.08 mm^3^, tetragonal, space group *P*4_3_2_1_2 (No. 96), *a* = *b* = 11.6748(4), *c* = 32.753(2) Å, *V* = 4464.3(4) Å^3^, *Z* = 8, *D*_c_ = 1.403 g/cm^3^, F_000_ = 1984, CCD Area Detector, MoKα radiation,λ = 0.71073 Å, *T* = 294(2) K, 2*θ*_max_ = 50.0°, 43048 reflections collected, 2346 unique (R_int_ = 0.0252). Final GooF = 1.051, *R1* = 0.0396, *wR2* = 0.1095, *R* indices based on 2198 reflections with I>2σ(I) (refinement on F^2^), 311 parameters, 0 restraints,*μ* = 0.108 mm^-1^. CCDC 914990 contains supplementary Crystallographic data for the structure. The detailed elucidation of crystal structure and analysis will be published elsewhere.

#### Tubulin purification

Tubulin devoid of microtubule-associated proteins (MAPs) was purified from bovine brain by cycles of temperature-dependent polymerization and depolymerization as described previously followed by phosphocellulose chromatography [51,52]. The concentration of the tubulin was determined by the method of Bradford using BSA as the standard [53]. Purified tubulin was quickly frozen as drops in liquid nitrogen and stored at - 80 °C until used. 

#### Tubulin binding assay

Noscapinoids quench the dichroic intrinsic tryptophan fluorescence of tubulin in a concentration-dependent fashion as described previously [26]. This has allowed an easy method for measuring the tubulin-noscapinoids interactions. Briefly, the compounds **5a, 6a-j** (0-200 µM) were incubated with 2 µM tubulin in PEM buffer (100 mM PIPES, pH 6.8, 3 mM MgSO_4_, and 1 mM EGTA) at 37 °C for 45 min. After incubation, the samples were excited at 295 nm. The relative intrinsic fluorescence intensity of the tubulin was then monitored in the absence and presence of the compound **5a, 6a-j** using a Varian Cary Eclipse Fluorescence Spectrophotometer (Agilent Technologies, CA, USA). The emission readings were recorded at 335 nm. We used a 0.3 cm path-length cuvette to minimize the inner filter effects caused by the absorbance of the noscapinoids. In addition, the inner filter effects were corrected using a formula *F*_corrected_ = *F*_observed·_antilog [(*A*_ex_ + *A*_em_)/2], where *A*_ex_ is the absorbance at the excitation wavelength and *A*_em_ is the absorbance at the emission wavelength. The equilibrium dissociation constant (K_D_) was determined by the formula: 1/a = K_D_/[free ligand] + 1, where *a* is the fractional occupancy and [free ligand] is the concentration of free ligand. The fractional occupancy (a) was determined by the formula *a* = Δ*F*/Δ*F*_max_, where Δ*F* is the change in fluorescence intensity when tubulin and its ligand are in equilibrium and Δ*F*_max_ is the value of maximum fluorescence change when tubulin is completely bound with its ligand. Δ*F*_max_ was calculated by plotting 1/Δ*F* versus 1/ligand, using total ligand concentration as the first estimate of free ligand concentration. Three independent experiments were performed for each noscapinoid. 

#### In vitro cell proliferation assay

The cell proliferation assay was performed in 96-well plates as described previously [54]. A panel of four cancer cell lines of different tissues of origins, namely: human lung adenocarcinoma epithelial cell line (A549), human lymphoblast cell line (CEM), human cervix cell line (HeLa) and human breast epithelial cell line (MCF-7) were studied. These cell lines were obtained from the National Repository of Animal Cell Culture, National Centre for Cell Sciences, Pune (NCCS), India. Briefly, 2 x10^3^ cells were seeded in each well and were incubated with gradient concentrations of **5a, 6a-j** compounds for 72 h. The cells were then fixed with 50% trichloroacetic acid and stained with 0.4% sulforhodamine B (SRB) dissolved in 1% acetic acid. Cells were then washed with1% acetic acid to remove excess (unbound) SRB. The protein-bound SRB was dissolved with 10 mM Tris base and the absorbance at 564 nm was determined using a SPECTRAmax PLUS 384 microplate spectrophotometer.

#### DAPI staining

Nuclear morphology of cells treated with the noscapinoids was evaluated by staining the cells with DAPI and imaging with fluorescence microscopy. Briefly, MCF-7 cells (2 x 10^3^ cells) were grown on poly-L-lysine coated coverslips in 6-well plates and were treated with the compounds at 25 μM for 72 hours. After incubation, coverslips were fixed in cold methanol and washed with PBS, stained with DAPI, and mounted on slides. Images were captured using a BX60 fluorescence microscope (Olympus, Tokyo, Japan) with an 8-bit camera (Dage-MTI, Michigan City, IN) and IP Lab software (Scanalytics, Fairfax, VA). Apoptotic cells were identified by features characteristic of apoptosis (e.g. nuclear condensation, formation of membrane blebs and apoptotic bodies).

#### Cell cycle analysis

 The flow cytometric evaluation of the cell cycle status was performed as described previously [26]. Briefly, cells (2 x 10^6^) treated with the noscapinoids up to 72 h were centrifuged, washed twice with cold PBS, and fixed in 70% ethanol. Tubes containing the cell pellets were stored at 4 °C for minimum of 24 h. Cells were then centrifuged at 1000 x g for 10 min and the supernatant was discarded. The pellets were washed twice with 5 ml of PBS and then stained with 0.5 ml of propidium iodide (0.1% of Pl + 0.6% Triton-X in PBS) and 0.5 ml of RNase A (2 mg/ml) for 45 minutes in dark. Samples were then analyzed on a FACS Calibur flow cytometer (Beckman Coulter Inc., Fullerton, CA).

## Results and Discussion

### Molecular modeling studies

**Figure 4:**
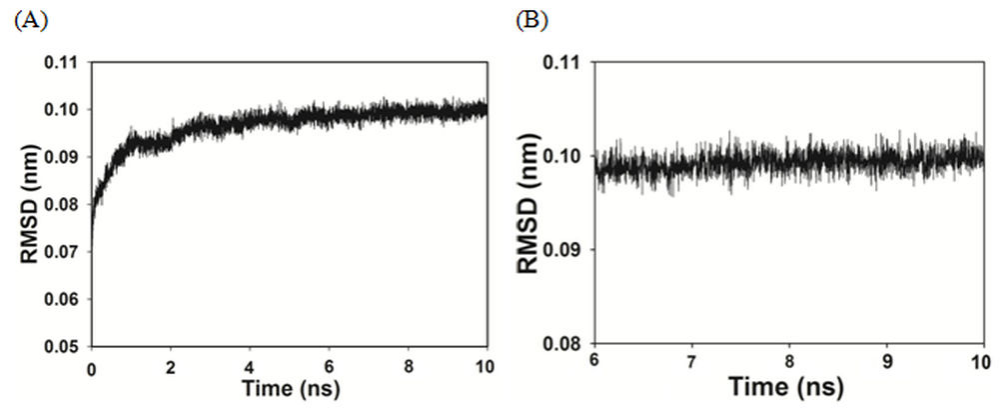
The relative fluctuation in the root-mean-square deviations (rmsd) of tubulin (αβ heterodimmer) during molecular dynamic (MD) simulation. (A) Time series of the rmsd for the Cα carbon atoms of tubulin during 10 ns of MD simulation starting from the initial structure. The relative fluctuation in the rmsd of the Cα atoms is very small after the initial equilibration (~3 ns) demonstrating the convergence of the simulation. (B) The relative fluctuation in the rmsd of the Cα atoms for the last 2000 frames that are used to generate the average structure of tubulin for docking study.

**Figure 5:**
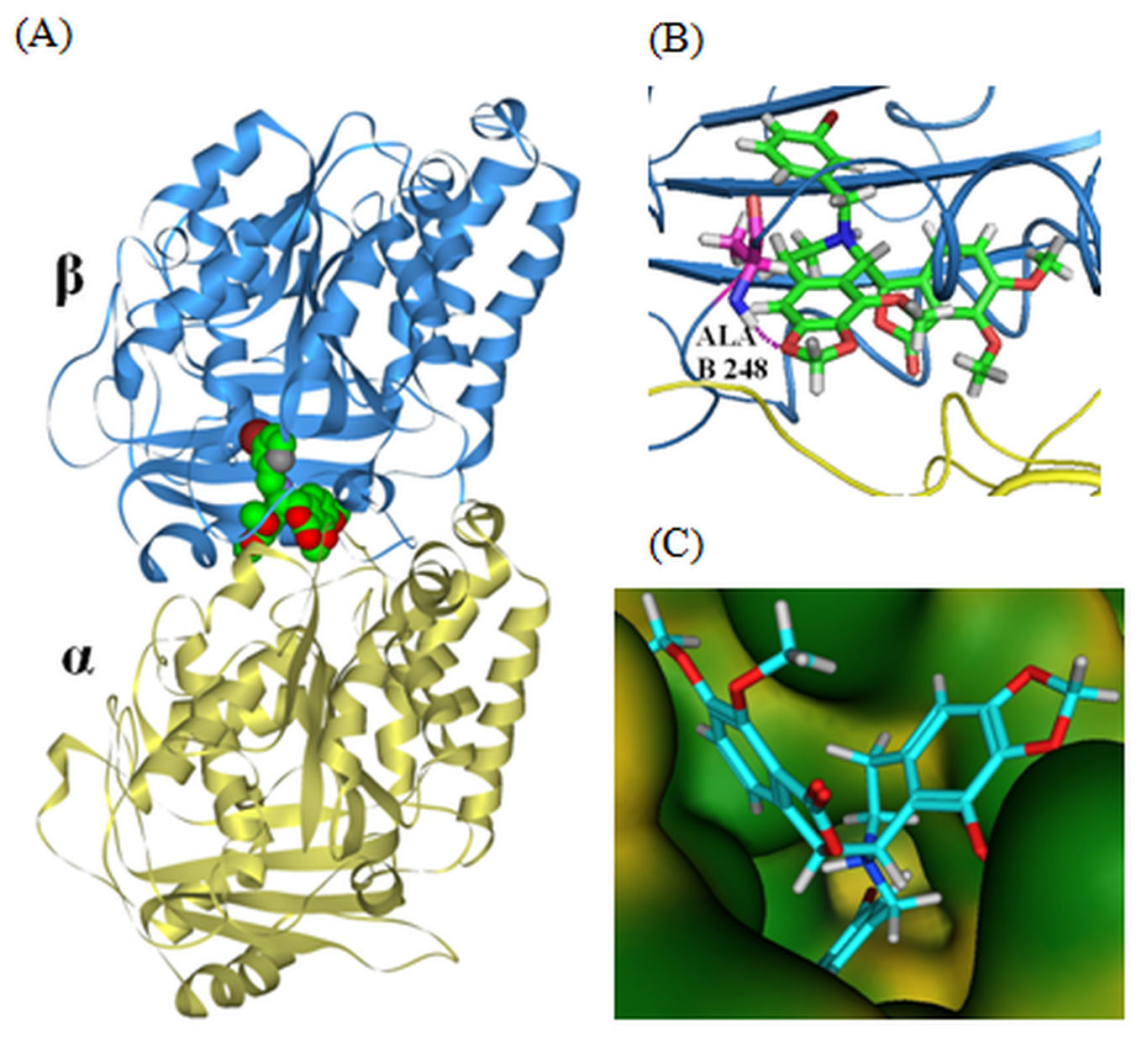
Typical snapshot of (A) docking complex of 6f (space field) bound to tubulin at colchicine binding site from molecular docking experiment. (B) The enlarge view of the docked complex with H-bonding. (C) The bound ligand **6f** is well-accommodated in the binding site.

**Figure 6:**
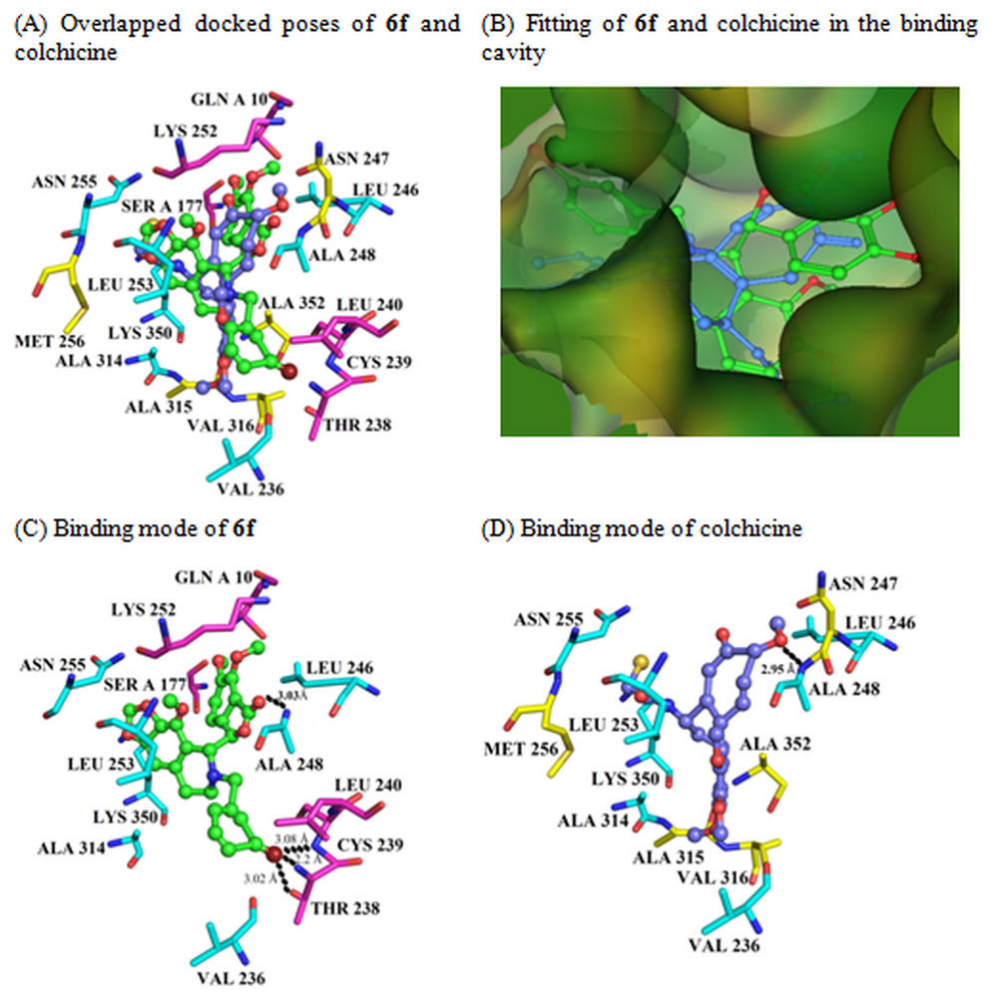
Three dimensional representation of the mode of interactions observed between the most potent noscapinoid, 6f and colchicine demonstrate distinct interaction with tubulin. **Panel A** represents snapshot of overlapped docked poses of **6f** (green) and colchicine (blue). The amino acids that are uniquely involved in the interaction of colchicine with tubulin are Asn 247, Met 256, Ala 315, Val 316, Ala 352 (yellow carbon). In contrast, the amino acids that are uniquely involved in the interaction of **6f** with tubulin are Gln A10, Ser A177, Thr 238, Cys 239, Leu 240, Lys 252 (pink carbon). The other amino acids that are common in the interaction of both **6f** and colchicine are Asn 255, Leu 253, Lys 350, Ala 314, Val 236, Ala 248 and Leu 246 (cyan carbon). Binding of colchicine has been highly biased towards β-tubulin whereas **6f** partially interacts with α-tubulin as well. **Panel** **B** represents the fitting of **6f** and colchicine inside the binding cavity. **Panel** **C** demonstrates the mode of interaction of **6f** with the residues of tubulin. The ligand, **6f**, distinctly interacts with the amino acid residues of tubulin in comparison to colchicine, and involves 4 hydrogen bonds (dashed lines): the bromine atom of **6f** hydrogen bonded with OG1 of Thr B238 (bond length 3.02 Å), N1 of Thr B238 (bond length 2.02 Å) and N1 of Cys B239 (bond length 3.08 Å). Similarly, the oxygen atom of the isobenzofuranone ring of **6f** hydrogen bonded with N1 of Ala B248 (bond length 3.03 Å). **Panel** **D** shows the binding mode of colchicine with tubulin. The interaction of colchicine with the residues of tubulin involves only one hydrogen bond (dashed line) between the oxygen atom of one of the methoxy group of colchicine with N1 of Ala B248 (bond length 2.95 Å). Only those amino acids that are within 4.5 Å distances from the docked ligands are shown.

Since noscapinoids binds to tubulin, we were interested in determining the binding affinity of newly designed congeners of noscapine with tubulin via molecular docking and LIE-SGB model building. The initial structure of tubulin was obtained from the PDB database, the missing amino acids were filled based on homology model building and the protein structure was refined further using MD simulation. The equilibration of the MD trajectories was monitored based on the convergence of plots of root-mean-square deviations (RMSDs) of Cα carbon atoms of tubulin (αβ heterodimmer) during 10 ns of MD simulation starting from the initial structure (Figure 4A). As can be seen from Figure 4A, the relative fluctuation in the rmsd of the Cα atoms is very small after the initial equilibration (~3 ns), demonstrating the convergence of the simulation. A total of 5000 frames were generated out of which the last 2000 frames were used to generate an average structure of the tubulin for the docking study. The relative fluctuation in the rmsd of the Cα atoms for the last 2000 frames is very small as shown in Figure 4B. A reasonable prediction model for calculating binding free energy (Δ*G*_*bind*_) of ligands was developed by utilizing the linear interaction energy (LIE) empirical equation (1). The α, β, and γ coefficients for the van der Waals, electrostatic, and cavity energy terms in the Surface Generalized Born (SGB) continuum solvent model were determined empirically by fitting to the experimentally determined binding free energies for a training set of ligands. The fitted equation considerably well represents the experimental binding free energy (R^2^ = 0.780) of the training set (Table 1). Glide XP_score_ also shows a better correlation with the experimental binding free energy (R^2^ = 0.615) of the training set (Table 1). Furthermore, the LIE-SGB empirical equation was used to predict the binding affinities of the newly designed congeners of noscapine **5a, 6a-j**. Free energy (Δ*G*_*bind*_) calculations of these compounds showed a range of -4.923 to -6.189 kcal/mol, and compound **6f** showed highest binding affinity to tubulin (-6.189 kcal/mol) (Table 2). Glide XP docking experiment corroborate similar findings; all the analogues, **5a, 6a-j** yielded better docking score (-7.252 to -5.402 kcal/mol) (Table 2) than the parent compound, noscapine (-5.505 kcal/mol) or the other existing, tested derivatives (-5.563 to -6.412 kcal/mol) (Table 1). The binding modes of **5a, 6a-j** involves interactions of the compounds with both α- and β- tubulin at a site very close to or overlapping with the well-characterized colchicine binding site (supporting information, Figure S1). In a representative example, compound **6f** binds with both α- and β- tubulin (Figure 5a) and was well accommodated inside the binding cavity (Figure 5b & 5c). Further, the binding mode of the most potent derivative **6f** is compared with the well characterized tubulin binding inhibitor, colchicine (Figure 6a-d). The overlapped docked poses of both **6f** (green) and colchicine (blue) at their binding site is shown in Figure 6a. The mode of interaction of colchicine is highly biased towards β-tubulin whereas **6f** partially interacted with α-tubulin as well. Both colchicine and **6f** were well accommodated inside the binding cavity due to availability of sufficient space (Figure 6b). The binding mode was represented in two steps: (a) receptor residues that have strong interactions with the ligand, such as a favorable hydrogen-bonding interactions, and (b) receptor residues that are close to the ligand, but whose interactions with the ligand are weak or diffuse, such as hydrophobic interaction. The mode of interactions of **6f** with the residues of tubulin was distinct in comparison to colchicine. As seen in Figure 6c, 6f distinctly interacted with the residues of tubulin in comparison to colchicine and the interaction involved 4 hydrogen bonds (dashed lines): the bromine atom of **6f** hydrogen bonded with OG1 of Thr B238 (bond length 3.02Å), N1 of Thr B238 (bond length 2.02 Å) and N1 of Cys B239 (bond length 3.08 Å). Similarly, the oxygen atom of the isobenzofuranone ring of **6f** hydrogen bonded with N1 of Ala B248 (bond length 3.03 Å). In contrast, the interaction of colchicine with the residues of tubulin involved only one hydrogen bond (dashed line) between the oxygen atom of one of the methoxy group of colchicine with N1 of Ala B248 (bond length 2.95 Å) (Figure 6d). Inspired by our computational findings, we synthesized all the compounds **5a, 6a-j** to evaluate their activities experimentally.

**Table 1:** Molecular docking results (Glide XP) as well as calculated energies based on LIE-SGB model of noscapine analogues: van der Waals (vdw), electrostatic (elec), cavity (cav), predicted and experimental binding free energy (Δ*G*_*bind*_).

Ligand	Glide XP_score_ (kcal/mol)	<U_vdw_> (kcal/mol)	<U_elec_> (kcal/mol)	<U_cav_> (kcal/mol)	Predicted ΔG_bind_ (kcal/mol)	K_D_ value (µM)	Experimental ΔG_bind_ (kcal/mol)
1	-5.505	-55.418	30.32	0.846	-4.946	152 ± 1.0	-5.246
2a	-5.563	-57.935	83.89	1.064	-5.603	81 ± 8.0	-5.587
2b	-6.337	-54.219	87.74	1.649	-5.911	40 ± 8.0	-6.006
2c	-5.684	-46.846	119.9	1.442	-5.344	54 ± 9.1	-5.827
2d	-5.868	-57.614	129.3	1.857	-6.560	22 ± 4.0	-6.360
2e	-5.790	-59.016	63.66	1.335	-5.837	86 ± 6.0	-5.551
2f	-6.412	-58.032	68.17	2.032	-6.452	14 ± 1.0	-6.628

<U_vdw_>, <U_elec_> and <U_cav_> energy terms represents the ensemble average energy terms calculated as the difference between bound and free state of the ligands and its environment. Experimental Δ*G*_*bind*_ was calculated from the dissociation constant (K_D_ value) using the relationship: Δ*G*_*bind*_ = *RT* ln K_D_ where T = 298 K and R = 0.00199 (kcal/mol.K). Predicted Δ*G*_*bind*_ was calculated using linear interaction energy (LIE) empirical equation: *ΔG*_*bind*_= 0.072⟨*U*_*vdw*_⟩−0.006⟨*U*_*elec*_⟩−0.951⟨*U*_*cav*_⟩. The K_D_ values are mean ± S.D. from three different experiments performed in triplicate.

**Table 2:** Molecular docking results (Glide XP) as well as calculated energies based on LIE-SGB model of third generation noscapine analogues (5a, 6a-j): van der Waals (vdw), electrostatic (elec), cavity (cav), predicted and experimental binding free energy (Δ*G*_*bind*_).

Ligand	Glide XP_score_ (kcal/mol)	<U_vdw_> (kcal/mol)	<U_elec_> (kcal/mol)	<U_cav_> (kcal/mol)	Predicted ΔG_bind_ (kcal/mol)	K_D_ value (µM)	Experimental ΔG_bind_ (kcal/mol)
5a	-5.639	-53.39	87.69	0.835	-5.164	68 ± 0.7	-5.691
6a	-5.997	-62.81	67.52	0.820	-5.707	-	-
6b	-6.918	-60.25	57.27	0.988	-5.622	-	-
6c	-6.087	-63.68	41.56	0.846	-5.639	91 ± 8.0	-5.518
6d	-6.882	-64.85	35.95	0.865	-5.708	-	-
6e	-6.907	-61.85	79.091	0.972	-5.852	-	-
6f	-7.252	-62.62	76.07	1.287	-6.189	38 ± 4.0	-6.036
6g	-5.767	-61.34	64.56	0.445	-5.227	-	-
6h	-7.196	-65.08	55.79	1.033	-6.003	-	-
6i	-5.712	-62.12	41.14	0.723	-5.407	79 ± 8.0	-5.602
6j	-5.402	-57.50	7.551	0.776	-4.923	228 ± 10.0	-4.973

Experimental Δ*G*_*bind*_ was calculated from the dissociation constant (K_D_ value) using the relationship: Δ*G*_*bind*_ = *RT* ln K_D_ where T = 298 K and R = 0.00199 (kcal/mol.K). Predicted Δ*G*_*bind*_ was calculated using linear interaction energy (LIE) empirical equation: *ΔG*_*bind*_= 0.072⟨*U*_*vdw*_⟩−0.006⟨*U*_*elec*_⟩−0.951⟨*U*_*cav*_⟩. The K_D_ values are mean ± S.D. from three different experiments performed in triplicate. The variation in binding affinity (K_D_ value) among the third generation α-Noscapine derivatives was statistically significant using 1-way anova test (F = 561.14, P < 0.001).

### Synthesis of noscapine derivatives

**Figure 7:**
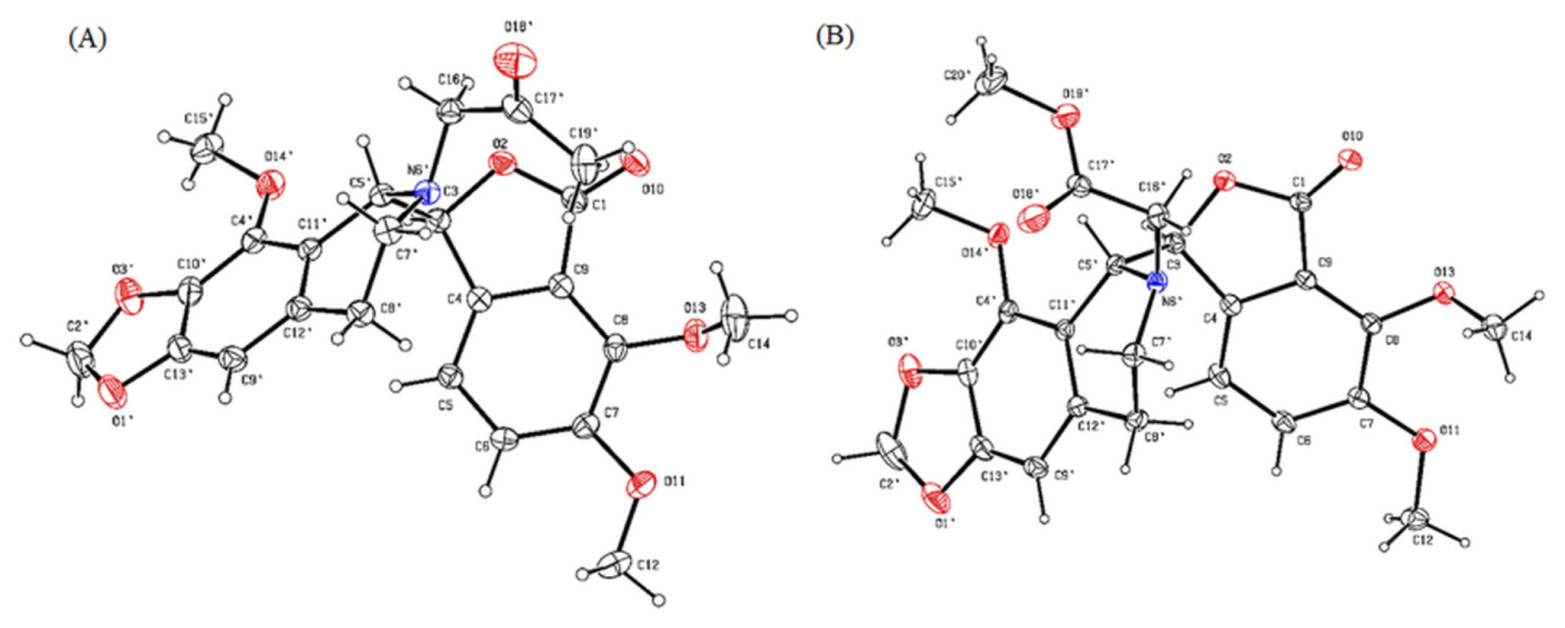
ORTEP representations of (A) 6h and (B) 6i with thermal displacement ellipsoids drawn at the 30% probability level and H atoms are represented by circles of arbitrary radii.

The synthetic approach for the preparation of noscapine derivatives **6a-j** is depicted in Figure 3. All these derivatives were synthesized from nornoscapine **5a** as starting material, which in turn was synthesized from noscapine. Briefly, commercially available natural noscapine **1** was reacted with meta-chloroperbenzoic acid (mCPBA) and acidified to generate the respective N-oxide HCl salt. It was then treated with FeSO_4_.7H_2_O (under modified non-classical Polonovski reaction conditions) [55] to give N-demethylated noscapine (nornoscapine) **5a** in 48% over all yields. **5a** was fully characterized by its IR, ^1^H & ^13^C NMR and Mass (ESI and HRMS) spectra data. It was further functionalized with various substituted benzyl halides and alkyl halides in the presence of base to yield new noscapinoids **6a-j**. Given the complexity in noscapine architecture and sensitivity of C-C bond between two heterocyclic lobs, alkylation was carried out using weaker bases such as carbonates rather than stronger bases like NaH, NaOH. Use of cesium carbonate as base resulted complex reaction mixture, whereas sodium carbonate gave low yield of product **6a**. Potassium carbonate in the presence of potassium iodide in acetone solvent was the best choice and alkylation proceeded well at room temperature to give desired products **6a-j** in excellent yields (92-98%) with > 96% HPLC purity. Choice of alkylation rather than acylation on isoquinoline is due to the fact that natural compound has methyl group and may be contributing to maintain requisite over all electron density of α-noscapine. All the products **5a** and **6a-j** were fully characterized by IR, ^1^H & ^13^C NMR and Mass (ESI and HRMS) spectra data (supporting information, Table S1). Single crystal X-ray analysis [50] of **6h** and **6i** unambiguously confirmed the structure (Figure 7A & B). X-ray diffraction data for **6h** (CCDC 914991) and **6i** (CCDC 914990) can be obtained free of charge from Cambridge Crystallographic Data Centre via www.ccdc.cam.ac.uk/data_request/cif. 

### Tubulin binding activity of noscapine analogues

**Figure 8:**
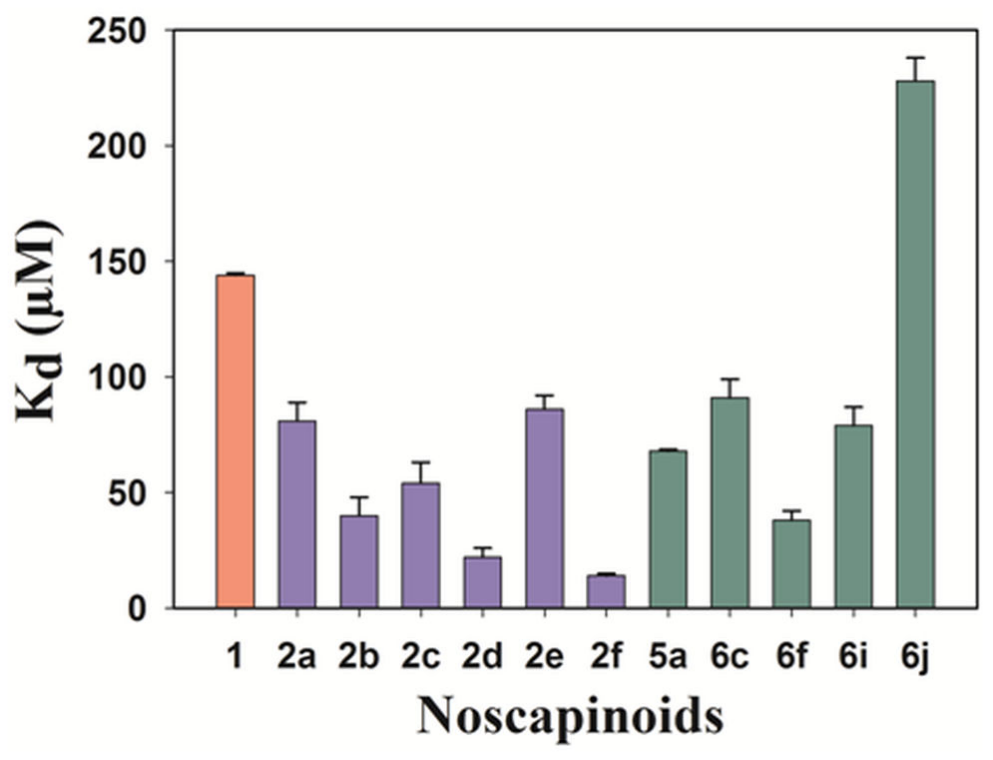
Bar diagram comparing K_D_ values of previously reported and the newly synthesized noscapinoids. The red bar represents the K_D_ value of noscapine, blue bars represent the K_D_ values of first generation noscapinoids12.13 and the green bars represent the K_D_ values of newly designed noscapinoids included in this manuscript.

Natural noscapine has been known to bind tubulin with an equilibrium dissociation constant (K_D_) of 144 µM [26]. To test if the new noscapine analogues **5a**, **6a-j** also bind to tubulin, we used the standard tubulin-binding assay (fluorescence quenching titration) [26,27] in the presence of different concentrations of these compounds. We found that the compounds **5a**, **6c**, **6f**, **6i-j** bind tubulin in a concentration-dependent manner (supporting information, Table S2), as evident from the quenching patterns of the intrinsic tryptophan fluorescence of tubulin with K_D_ values ranging from 38 µM to 228 µM (Figure 8; Table 2). Experimental Δ*G*_*bind*_ for these analogues was calculated from the dissociation constant (K_D_ value) using the relationship: Δ*G*_*bind*_ = *RT* ln K_D_ where T = 298 K and R = 0.00199 (kcal/mol.K). In fact, these experimentally determined values of Δ*G*_*bind*_ considerably better correlated (R^2^ = 0.675) with the predicted values of Δ*G*_*bind*_ (Table 2) suggesting that the LIE-SGB method is reasonably accurate in rational design of potent noscapinoids. The other analogues **6a-b**, **6d-e** and **6g-h** were also showed potential binding to tubulin as indicated by their ability to quench the intrinsic tryptophan fluorescence of tubulin. However, we could not obtain reliable and reproducible K_D_ values for these compounds due to the non-linear nature of the quenching pattern and higher absorbance of the compounds at the excitation and emission wavelengths (Data not shown). Among all the new series of noscapine analogues, **6f** was found to be most reliable and effective tubulin binding agent (K_D,_ 38 ± 4 µM) in comparison to many of the first generation potent analogues (Figure 8) reported previously [21,24,26-28].

### Effect of compounds 5a, 6a-j on proliferation of cancer cells

**Figure 9:**
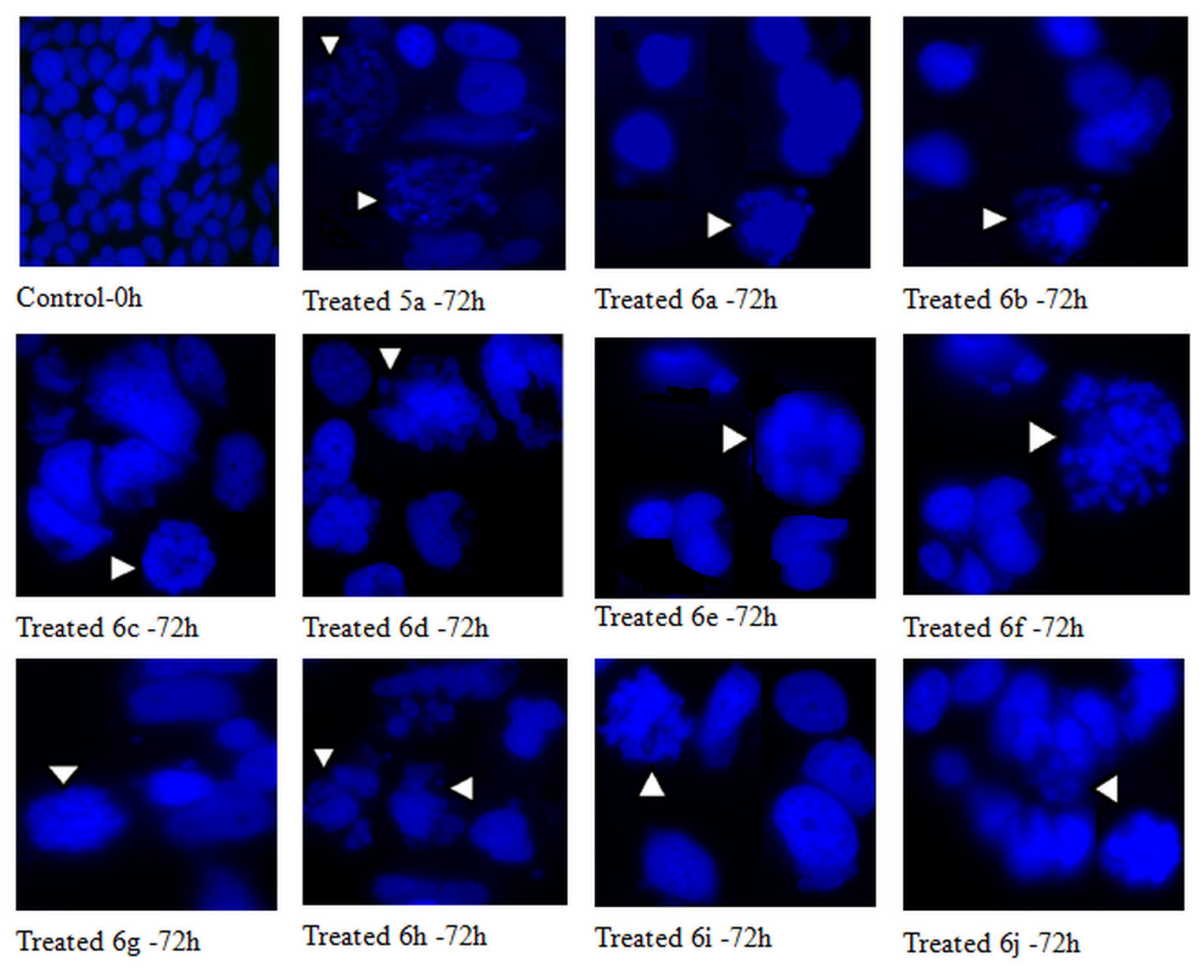
Morphologic indicators of apoptotic cell death include chromatin condensation along the nuclear envelope and plasma membrane blebbing followed by formation small apoptotic bodies. Panels show morphological evaluation of nuclei stained with DAPI in the absence and presence of the analogues **5a** and **6a-j** (25 µM each). Several typical features of apoptotic cells such as condensed chromosomes, numerous fragmented micronuclei, and apoptotic bodies are evident (indicated by white head arrows) upon 72 hours of drug treatment. (Scale bar = 15 µm).

A pharmacological study at the cellular level was carried out to determine if all the analogues **5a, 6a-j** affect cancer cell proliferation. As a preliminary screen, all compounds including the lead compound noscapine were evaluated for their anti-proliferative activity in a variety of human cancer cell lines of different tissues in origin such as lymphoblastoid cells (CEM), cervix (HeLa), lung (A549) and breast (MCF-7). Each compound was dissolved in DMSO to provide a concentration range of 10 nM to 1000 µM. We used a sulforhodamine B (SRB) *in vitro* proliferation assay to determine the IC_50_ values (the drug concentration required for a 50% inhibition of cell proliferation) of each compound. The IC_50_ values for the analogues **5a, 6a-j** for these four cell lines are included in Table 3. Our results showed that all the cancer cell types were more susceptible to compounds **5a, 6a-j** in comparison to the lead compound, noscapine, with lower IC_50_ value. It is evident that tubulin represents a potential target for these new series of compounds. We also found that the IC_50_ values do not show a correlation among these analogues and were cell-type dependent. Moreover, the IC_50_ values of these novel analogues were found to be better in comparison to many of the first generation analogues reported previously using CEM cancer cell line [22]. Besides the anti-proliferative effect, DAPI staining of the cells treated with noscapinoids showed condensed chromatin along with numerous fragmented nuclei (shown by white arrow heads), indicative of apoptotic cell death (Figure 9).

**Table 3:** IC_50_ values (a drug concentration required to achieve a 50% inhibition of cellular proliferation) of noscapine derivatives 5a & 6a-j for various cancer cell types^**a**^.

Noscapine analogue	CEM (µM)	HeLa (µM)	A549 (µM)	MCF-7 (µM)
5a	8.9 ± 0.6	20.9 ± 1.8	41.6 ± 2.1	33.6 ± 1.3
6a	8.3 ± 0.4	19.4 ± 1.4	37.7 ± 1.8	31.2 ± 1.5
6b	9.0 ± 0.4	21.2 ± 2.1	42.4 ± 2.3	34.1 ± 1.6
6c	10.0 ± 0.8	23.7 ± 1.5	49.0 ± 2.5	38.2 ± 2.5
6d	7.7 ± 0.3	17.9 ± 0.9	33.5 ± 1.7	28.7 ± 0.8
6e	7.5 ± 0.6	17.3 ± 0.7	32.1 ± 1.6	27.8 ± 1.3
6f	6.7 ± 0.3	15.3 ± 0.5	26.9 ± 1.4	24.5 ± 0.8
6g	11.9 ± 0.8	28.3 ± 2.1	61.3 ± 3.1	45.8 ± 2.2
6h	6.9 ± 0.5	15.8 ± 0.5	28.0 ± 1.5	25.3 ± 1.2
6i	9.5 ± 0.7	22.3 ± 1.2	45.4 ± 2.5	36.0 ± 1.9
6j	13.6 ± 1.3	32.4 ± 2.4	72.3 ± 3.5	52.6 ± 3.8
Nos	14.5 ± 2.5	24.0 ± 2.9	72.9 ± 4.6	42.3 ± 2.7

a Cancer cells used in the assay namely, CEM: human lymphoblast cell line, HeLa: human cervix cell line, A549: human lung adenocarcinoma epithelial cell line and MCF7: human breast epithelial cell line. Each value represents mean ± S.D. from three different experiments performed in triplicates. The variation in IC_50_ values are statistically significant among the third generation α-Noscapine derivatives (F = 169.93, P < 0.001) as well as among different cancer cell lines (F = 1530.48, P < 0.001) based on 2-way anova test.

### Effects of analogues 5a, 6a-j on cell cycle progression in cancer cell

**Figure 10:**
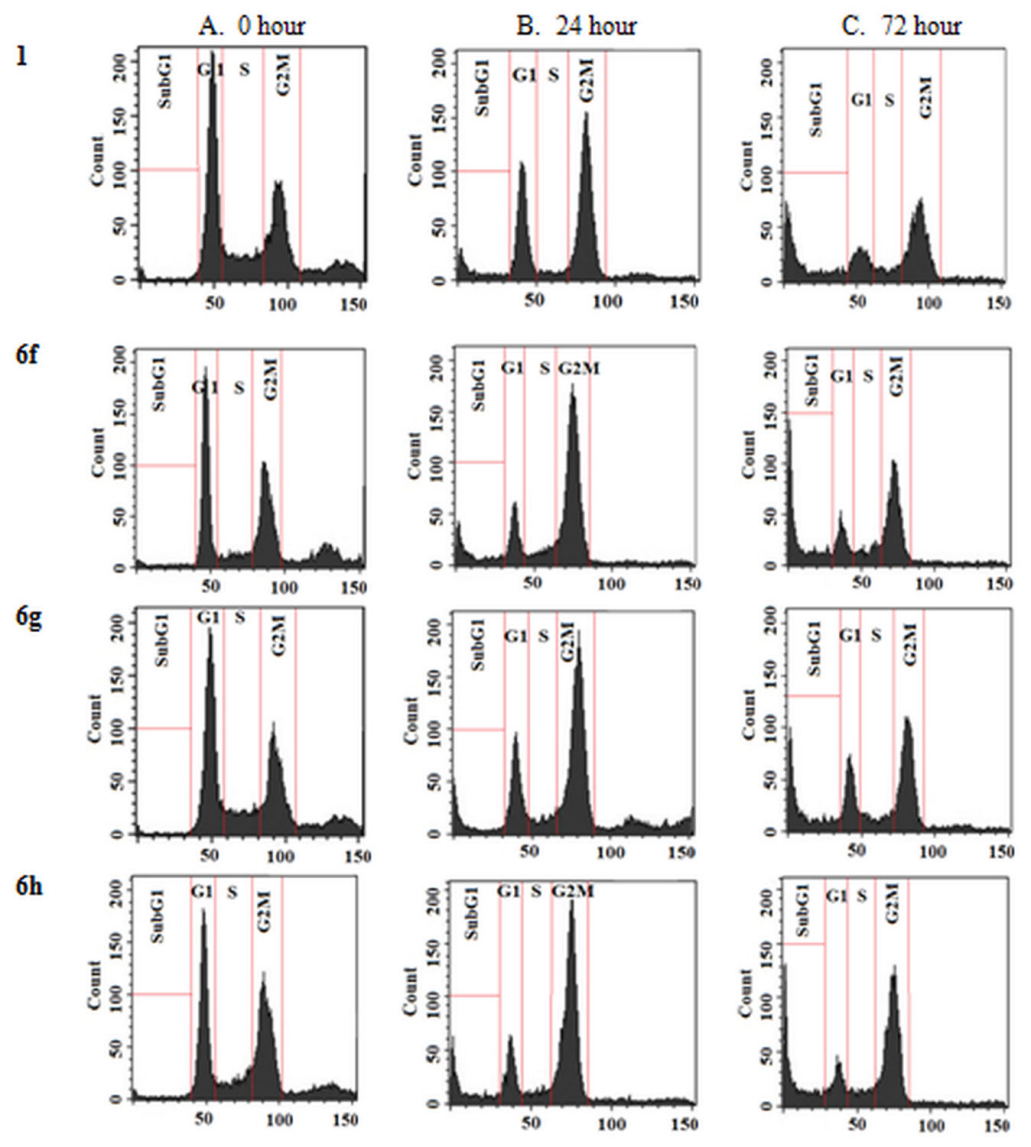
Noscapine (1) and its analogues 6f-h inhibit cell cycle progression at mitosis followed by the appearance of a characteristic hypodiploid (sub-G1) DNA peak, indicative of apoptosis. Panels A-C depict analyses of cell cycle distribution in a two-dimensional disposition as determined by flow cytometry in MCF-7 cells treated with 25 µM of noscapine (1), **6f, 6g**, and **6h** for 0, 24 and 72 hours, respectively.

**Figure 11:**
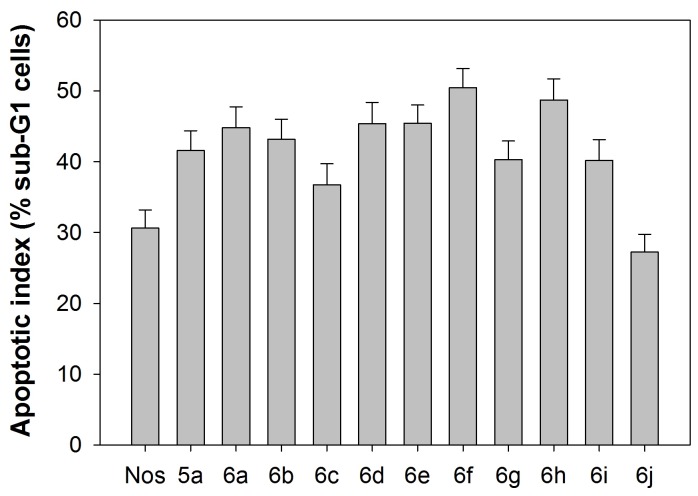
Graphical representation of the quantitation of apoptotic index (percent sub-G1 cells) at 25 µM at 72 hours for all the compounds. Results are representative of three experiments performed in triplicate. The variation in apoptotic index among the third generation α-Noscapine derivatives is statistically significant (F = 18.30, P < 0.001) based on 1-way ANOVA test.

Microtubule-interfering agents, including noscapine [12], are known to arrest cell cycle progression at the G2/M phase in mammalian cells [56]. Therefore, we next investigated the effect of analogues **5a, 6a-j** on the percentage of G2/M and sub-G1 cell populations in MCF-7 cancer cells as a function of time using fluorescence activated cell sorting (FACS) analysis. We studied the effect of all these compounds at a concentration of 25 µM for 0, 24 and 72 hours of drug treatment. Figure 10 shows the representative cell cycle profiles for the compounds **6f-h**. The DNA content of a cell is a reliable indicator of cell cycle progression and cell death [29,30]. Treatment of MCF-7 cells with the analogues for 24 and 72 hours led to considerable perturbations in the cell cycle progression at 25 µM (Figure 10) as indicated by their FACS profiles. Specifically, the two-dimensional FACS profiles indicated a pronounced increase in the population of cells that accumulated with less than 2N DNA (sub-G1 population) at 72 h, indicative of the dead and dying cells. This is preceded by a massive accumulation of cell populations with 4N DNA indicating G2/M arrest. The distribution of cell populations over G0/G1, S, G2/M and sub-G1 phases of the cell cycle for 25 μΜ drug treatment is shown in Table 4. The anti-cancer activity of all the analogues was thus evident in their ability to produce a significant sub-G1 population having hypodiploid (2N) DNA content that reflects fragmented DNA, a characteristic of apoptosis. The apoptotic index (percent sub-G1 population) has been plotted for all the analogues in Figure 11, showing the extent of their deleterious effect in comparison to noscapine on the cell.

**Table 4:** Effect of noscapine derivatives (5a & 6a-j) on cell cycle progression of MCF-7 cells.^a^

Cell cycle parameters %	0 hour				24 hours				72 hours			
	Sub-G_1_	G_0_/G_1_	S	G_2_/M	Sub-G_1_	G_0_/G_1_	S	G_2_/M	Sub-G_1_	G_0_/G_1_	S	G_2_/M
Noscapine	0.29	58.85	10.24	24.29	7.24	19.48	4.19	62.38	30.62	19.05	8.53	37.36
5a	0.27	63.37	12.93	19.45	8.46	13.47	4.08	66.37	41.57	10.74	7.08	39.25
6a	1.44	67.06	9.53	20.17	8.12	16.28	4.94	63.18	44.82	9.06	7.39	37.72
6b	0.74	55.39	12.17	28.79	7.37	19.42	4.16	64.42	43.18	11.38	7.53	35.84
6c	0.82	60.66	11.49	24.29	8.08	20.72	4.85	63.49	36.73	15.38	8.27	38.41
6d	0.52	56.82	12.28	28.39	7.27	22.73	4.62	59.41	45.38	8.29	7.83	36.18
6e	0.87	62.57	11.93	23.15	8.17	12.84	4.57	68.42	45.42	7.02	6.83	38.52
6f	1.04	64.26	9.51	22.47	9.47	11.82	6.29	67.48	50.44	6.38	4.72	37.29
6g	0.62	63.32	12.92	21.37	10.86	20.94	4.08	62.28	40.29	12.76	8.07	37.93
6h	0.35	61.16	12.29	25.09	11.37	12.25	5.92	68.58	48.73	7.19	6.28	36.18
6i	0.46	57.41	11.08	27.14	12.25	19.57	4.62	63.38	40.18	14.37	6.43	37.15
6j	0.39	59.15	9.47	24.35	8.29	25.71	3.09	66.17	27.24	22.16	8.63	39.48

a MCF-7 cells were treated with 25 µM solution for the indicated time (h) and then stained with propidium iodide for cell cycle analysis.

## Conclusions

In summary, we reported a new series of noscapine derivatives (we call them third- generation noscapinoids) **5a**, **6a-j**. These compounds were successfully designed by introducing structural modification of methyl substituent at ‘N’ in isoquinoline unit of natural α-noscapine **1**. All compounds were well accommodated within the binding cavity on tubulin with better docking score and predicted binding free energy. Inspired by computational evaluation, the new congeners of noscapine **5a, 6a-j** were chemically synthesized in high yield, and were fully characterized by spectral data. Tubulin binding assay indicated that the newly designed noscapinoids bind to tubulin with a greater affinity in the following order of magnitude: 6f > 5a > 6i > 6c > 6j. N-(3-brormobenzyl) noscapine (6f) binds tubulin with higher affinity (K_D_, 38 ± 4 µM) in comparison to parent natural noscapine (K_D_, 144 ± 2.8 µM) and first generation clinical candidate, 9-bromonoscapine (K_D_, 54 ± 9.1 µM). Moreover, the new congeners of noscapine **5a, 6a-j** exhibited improved therapeutic potential for a variety of cancer cell types. Interestingly, N-(3-brormobenzyl) noscapine **6f** showed a pronounced anti-proliferative activity against all the cell lines used in the study compared to the lead compound, noscapine. Furthermore, the mechanism of cell death caused by this series of noscapine analogues is preserved, in that, like noscapine, cell death was preceded by extensive mitotic arrest. Treatment of MCF-7 cells with **6f** led to profound perturbations of the cell cycle profile with progressive generation of cells having hypodiploid DNA content, which is a reflection of fragmented DNA, indicative of cell death. The apoptotic index for this compound is higher than noscapine, showing its superior deleterious effect on cell cycle progression and ability to induce cell death. Taken together, N-(3-brormobenzyl) noscapine **6f** hold a great potential as potent cytotoxic compound for further preclinical and clinical evaluation. 

## Supporting Information

Figure S1 **Typical snapshot of (**A**) newly designed noscapinoids 5a and 6a-j (blue stick) bound to tubulin at colchicine binding site from molecular docking experiment.** (B) The enlarge view of the colchicine binding site with the bound noscapinoids **5a** and **6a-j**. (C) The bound noscapinoids **5a** and **6a-j** are well-accommodated in the binding site.
(TIF)

Table S1 **Binding of new noscapinoids 5a, 6c, 6f, 6i-j to tubulin as measured by fluorescence quenching of tubulin.** (A) quenching of tubulin fluorescence emission by noscapinoids **5a, 6c, 6f, 6i-j** in a concentration-dependent manner (control 0 μM (♦), 50 μM (■), 100 μM (▲) and 200 μM (x)). (B) double-reciprocal plot showing a dissociation constant (K_d_) of compounds (5a & 6a-j) binding to tubulin.
(DOCX)

Table S2 **^1^H NMR, ^13^C NMR and ESI / HRMS spectra of noscapinoids 5a and 6a-j.**
(DOCX)
