# Supplementary figures and images for: Rational Design, Synthesis, and Biological Evaluation of Third Generation α-Noscapine Analogues as Potent Tubulin Binding Anti-Cancer Agents
Source: PLoS One. 2013 Oct 21;8(10):e77970. doi: 10.1371/journal.pone.0077970 (PMC3804772; doi:10.1371/journal.pone.0077970)

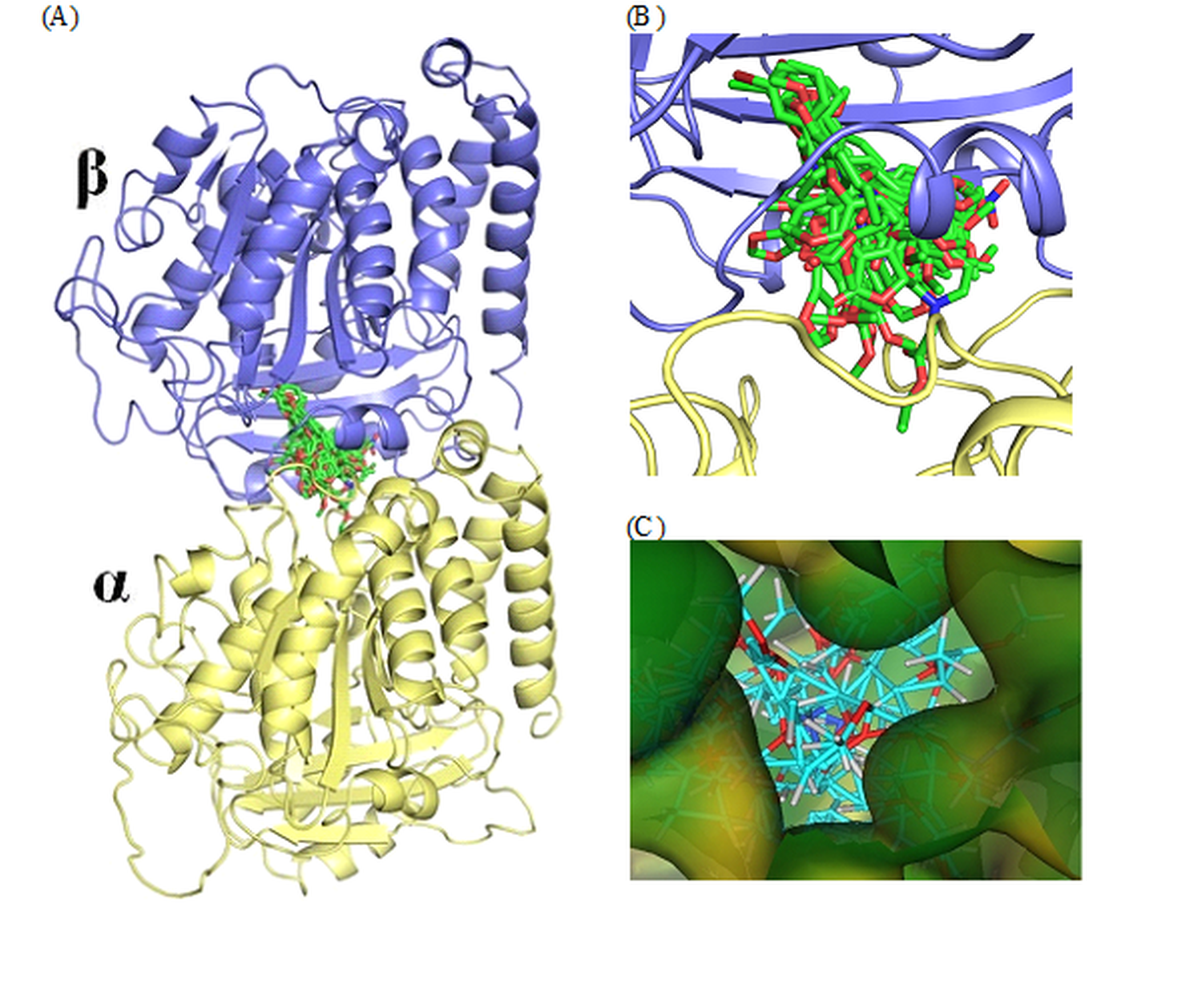

Supplement: Figure S1 — Typical snapshot of (A) newly designed noscapinoids 5a and 6a-j (blue stick) bound to tubulin at colchicine binding site from molecular docking experiment. (B) The enlarge view of the colchicine binding site with the bound noscapinoids 5a and 6a-j. (C) The bound noscapinoids 5a and 6a-j are well-accommodated in the binding site. (TIF) [file pone.0077970.s001.tif]
