# Supplementary material for: Rational Design, Synthesis, and Biological Evaluation of Third Generation α-Noscapine Analogues as Potent Tubulin Binding Anti-Cancer Agents
Source: PLoS One. 2013 Oct 21;8(10):e77970. doi: 10.1371/journal.pone.0077970 (PMC3804772; doi:10.1371/journal.pone.0077970)
Supplement: Table S1 — Binding of new noscapinoids 5a, 6c, 6f, 6i-j to tubulin as measured by fluorescence quenching of tubulin. (A) quenching of tubulin fluorescence emission by noscapinoids 5a, 6c, 6f, 6i-j in a concentration-dependent manner (control 0 μM (♦), 50 μM (■), 100 μM (▲) and 200 μM (x)). (B) double-reciprocal plot showing a dissociation constant (Kd) of compounds (5a & 6a-j) binding to tubulin. (DOCX) [file pone.0077970.s002.docx]

**Table S1:** ^1^H NMR, ^13^C NMR and ESI / HRMS spectra of noscapinoids **5a** and **6a-j.**

**^1^H NMR spectra of 5a.**


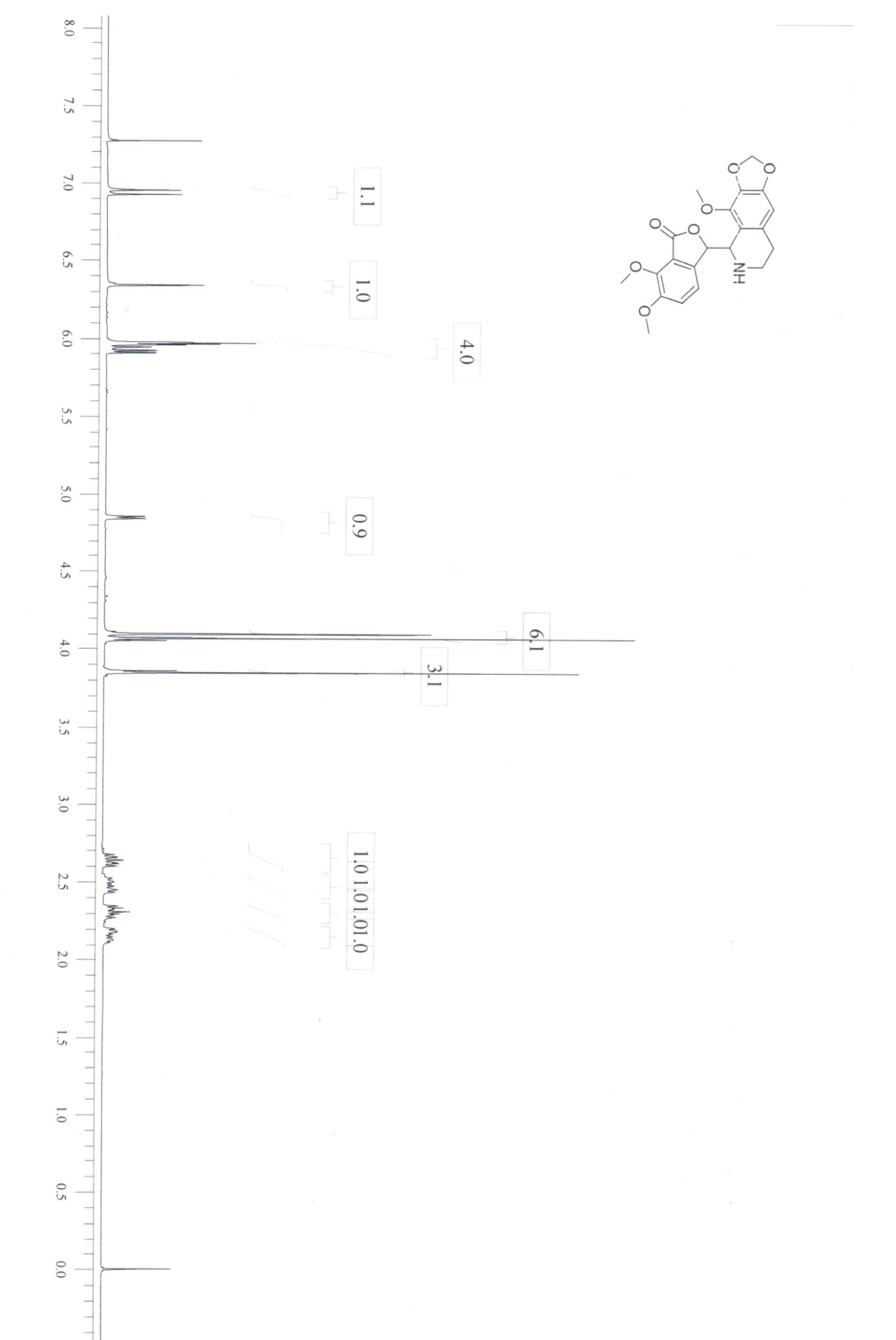


**^13^C NMR spectra of 5a.**


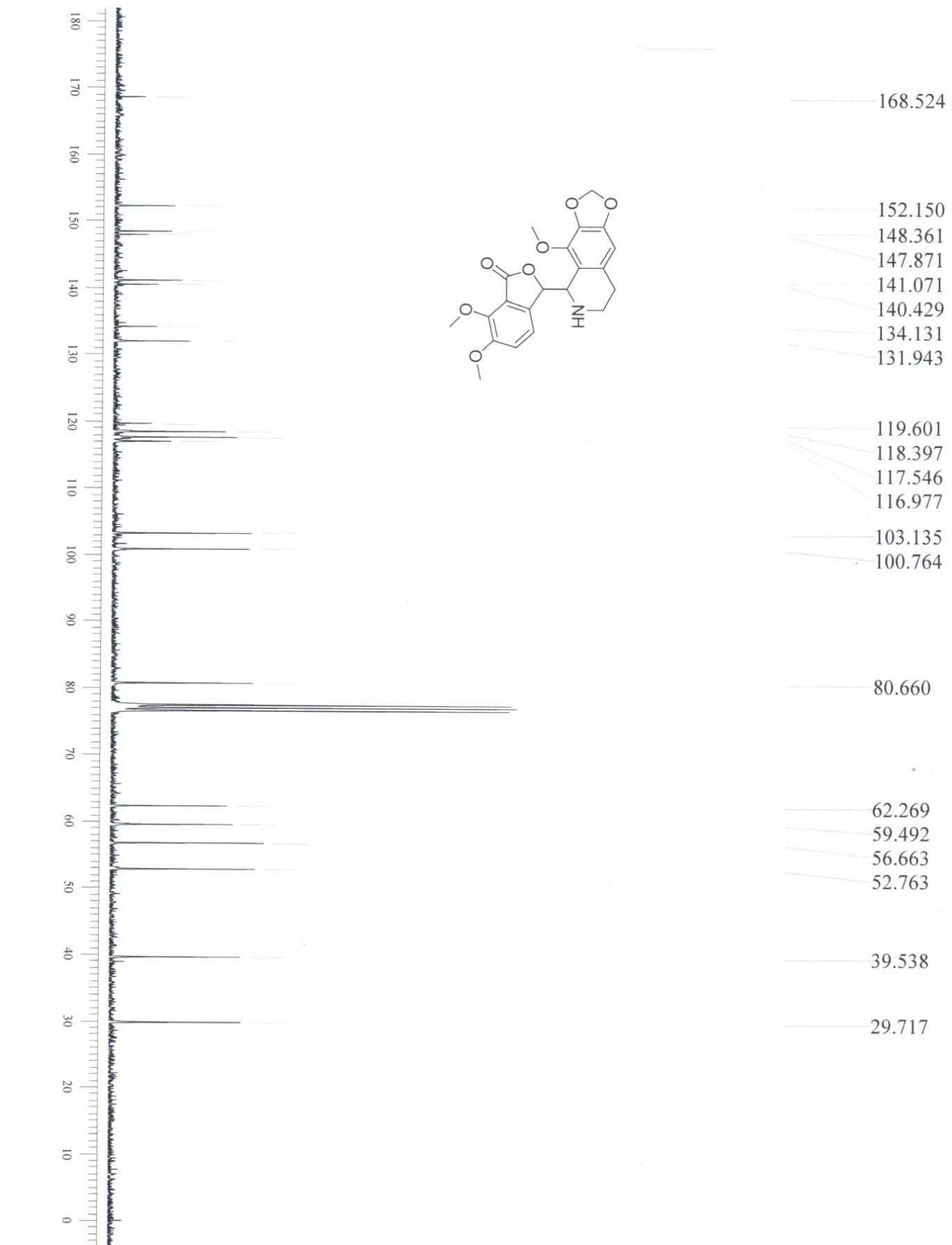


**ESI spectra of 5a.**


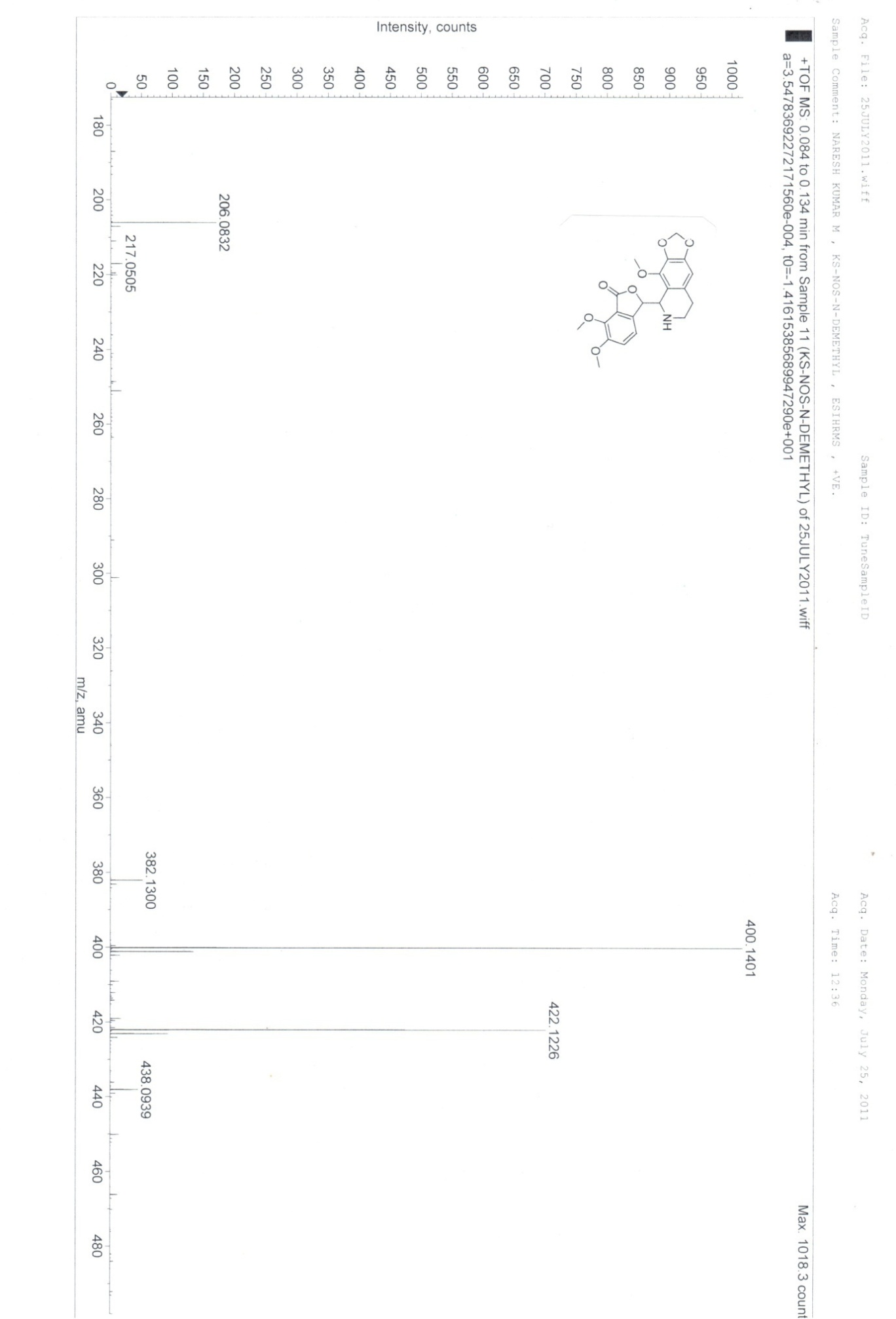


**HRMS spectra of 5a.**


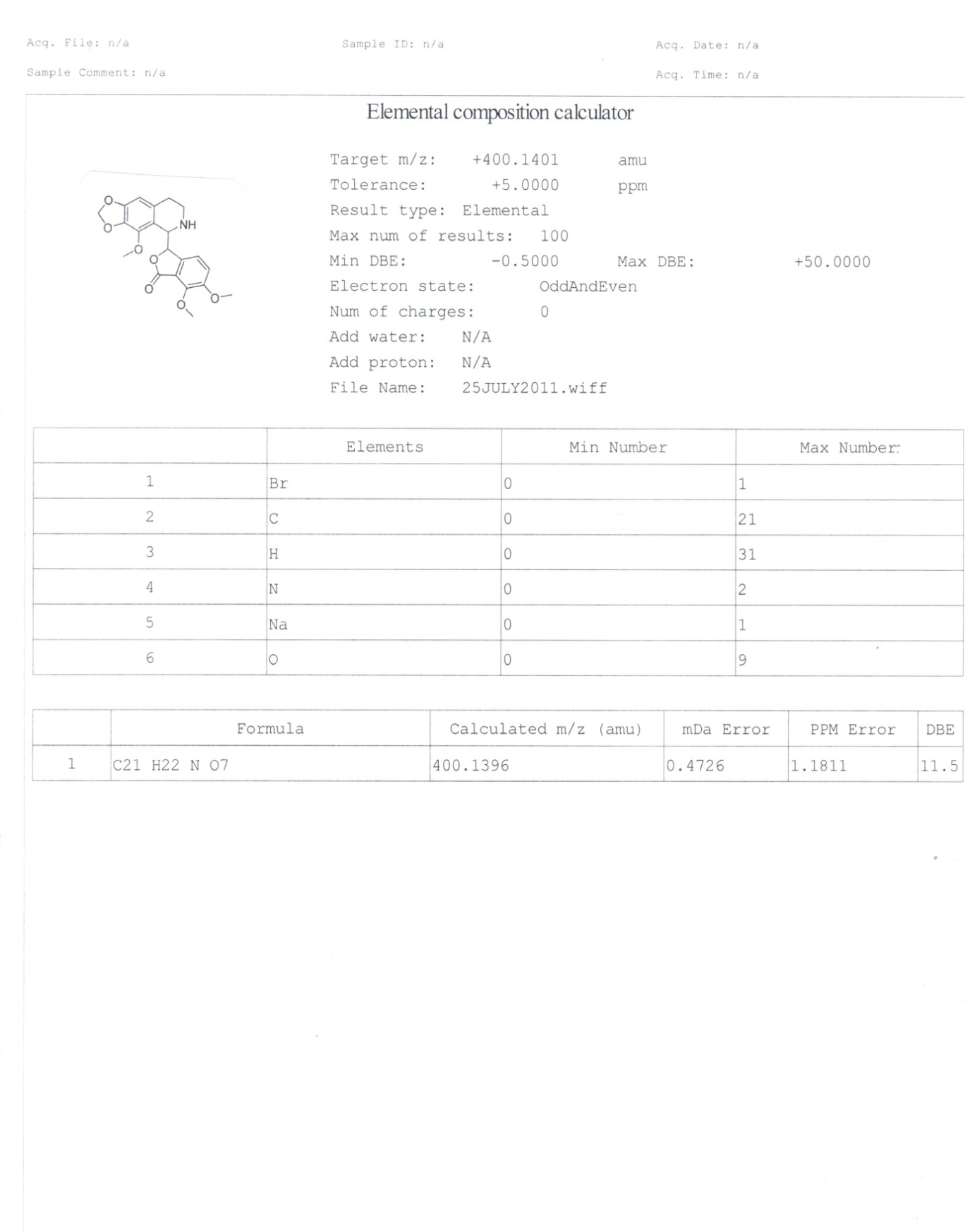


| **^1^H NMR spectra of 6a.** |
| --- |
| 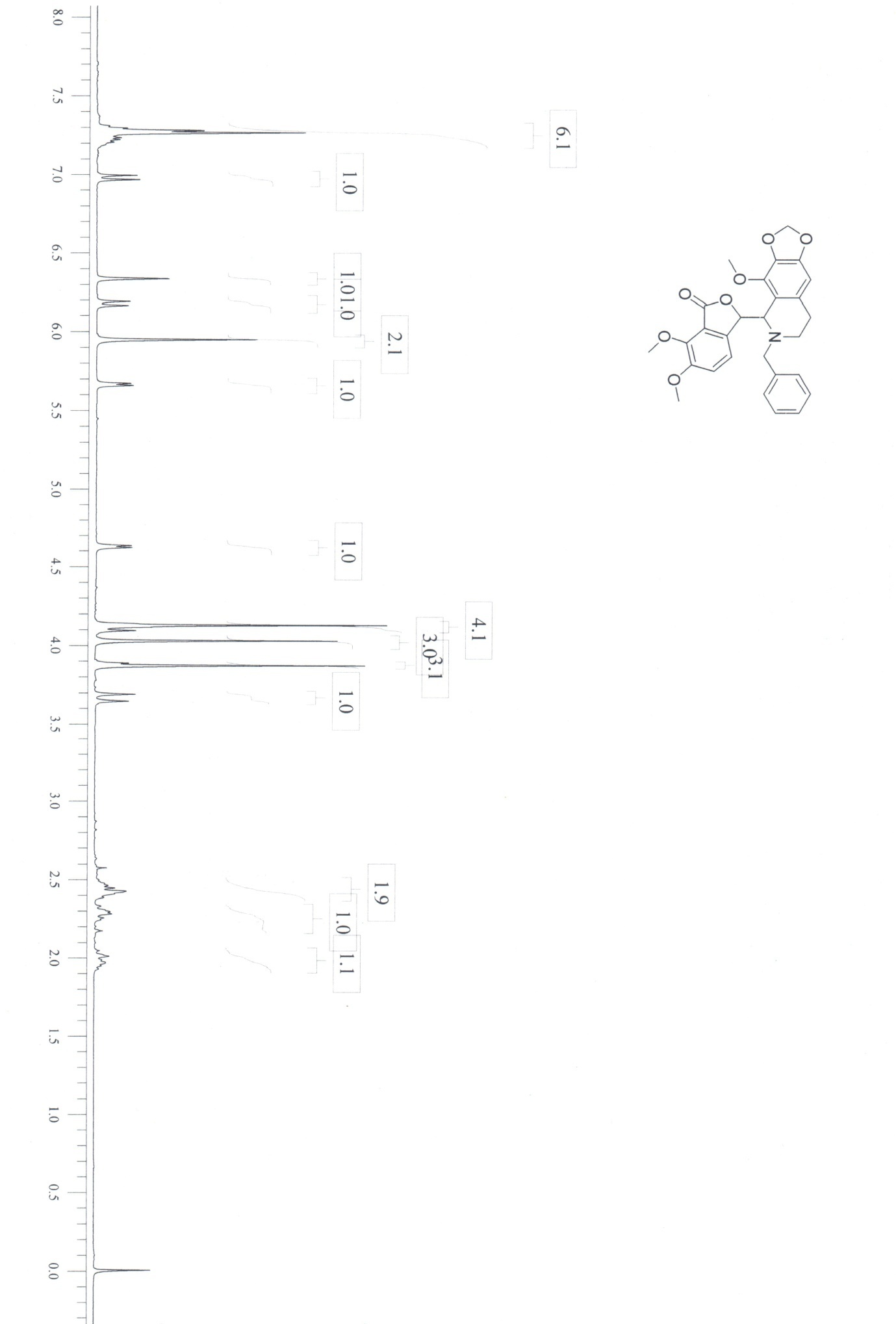 |

| **^13^C NMR spectra of 6a.** |
| --- |
| 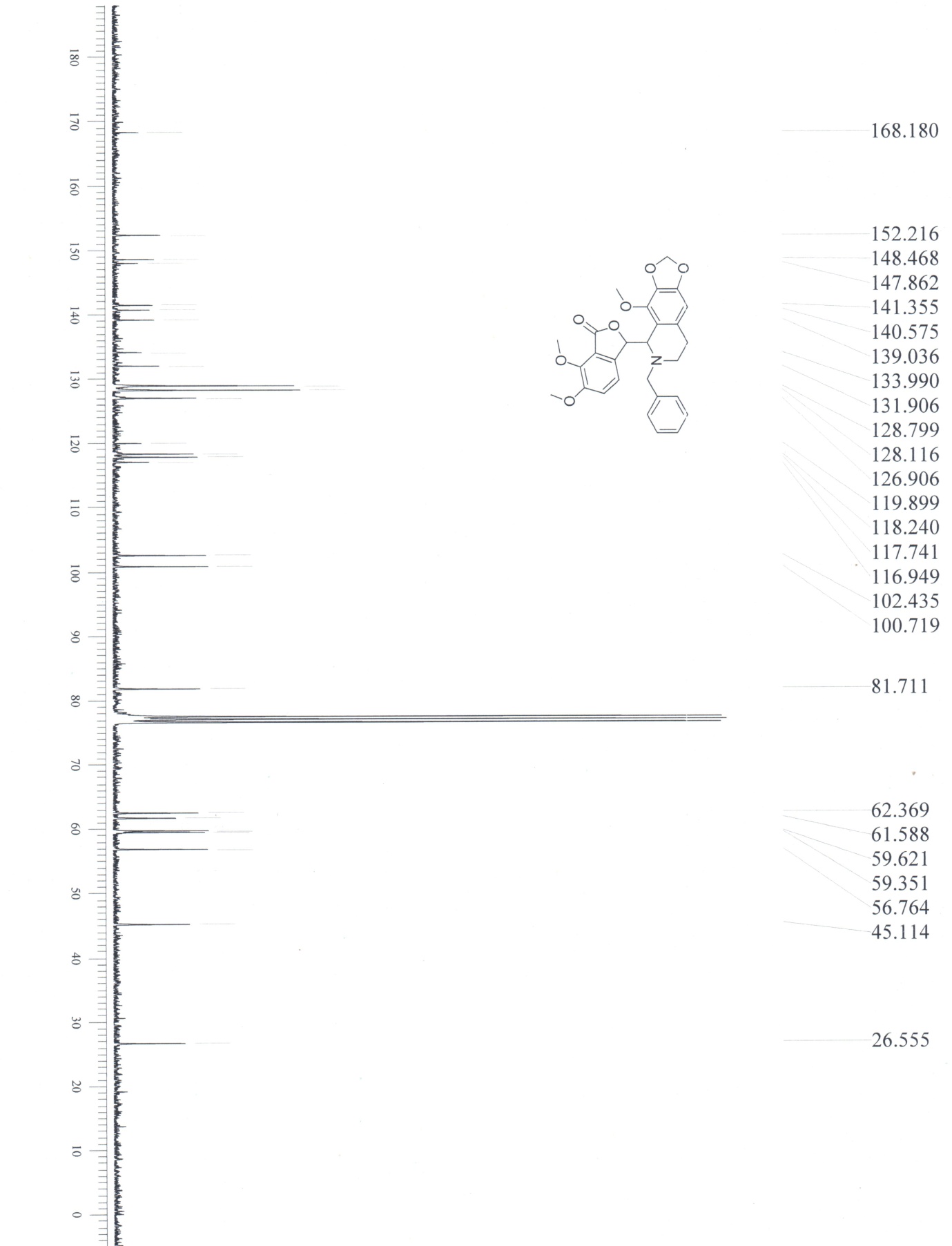 |

| **ESI spectra of 6a.** |
| --- |
| 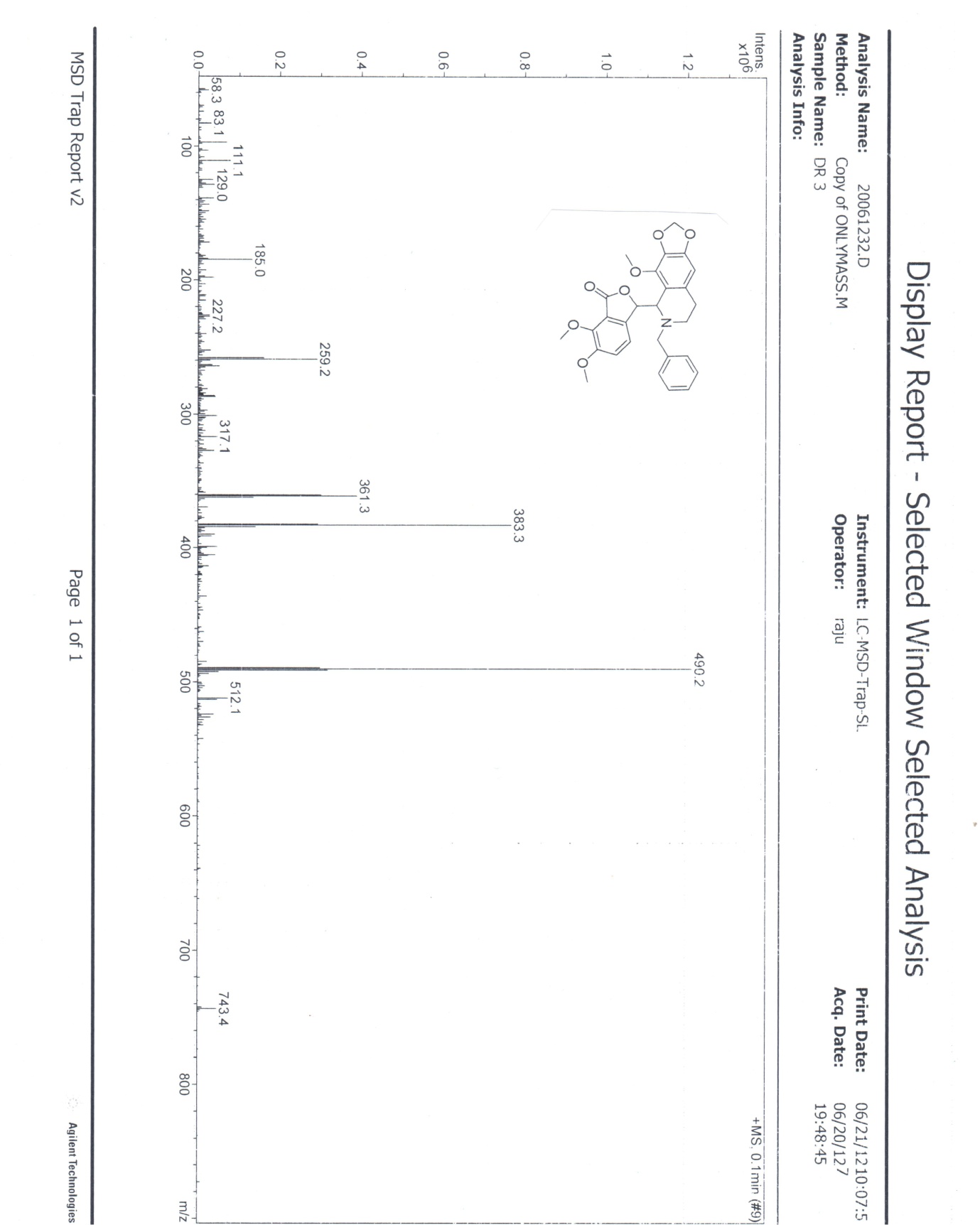 |

| **^1^H NMR spectra of 6b.** |
| --- |
| 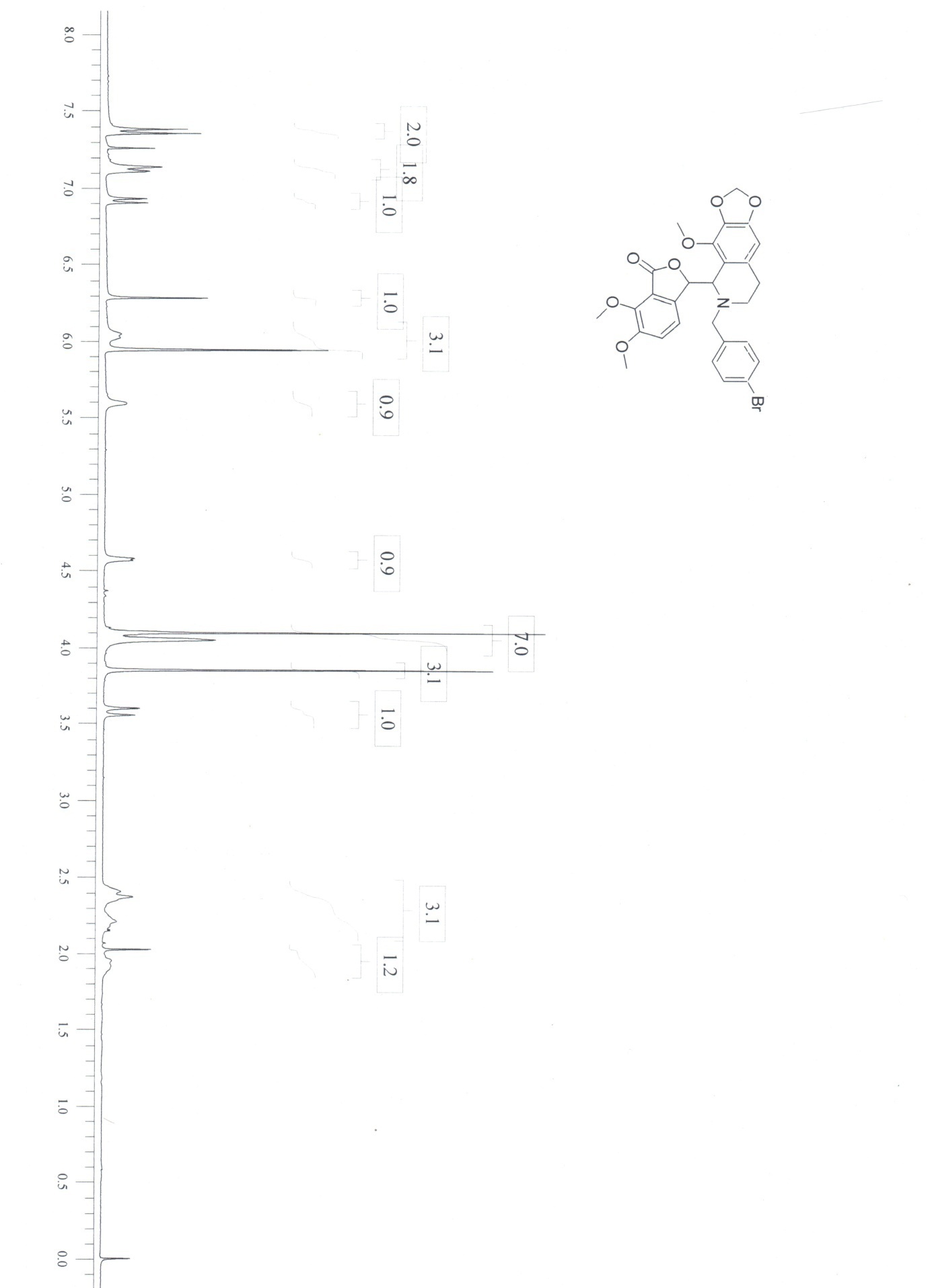 |

| **^13^C NMR spectra of 6b.** |
| --- |
| 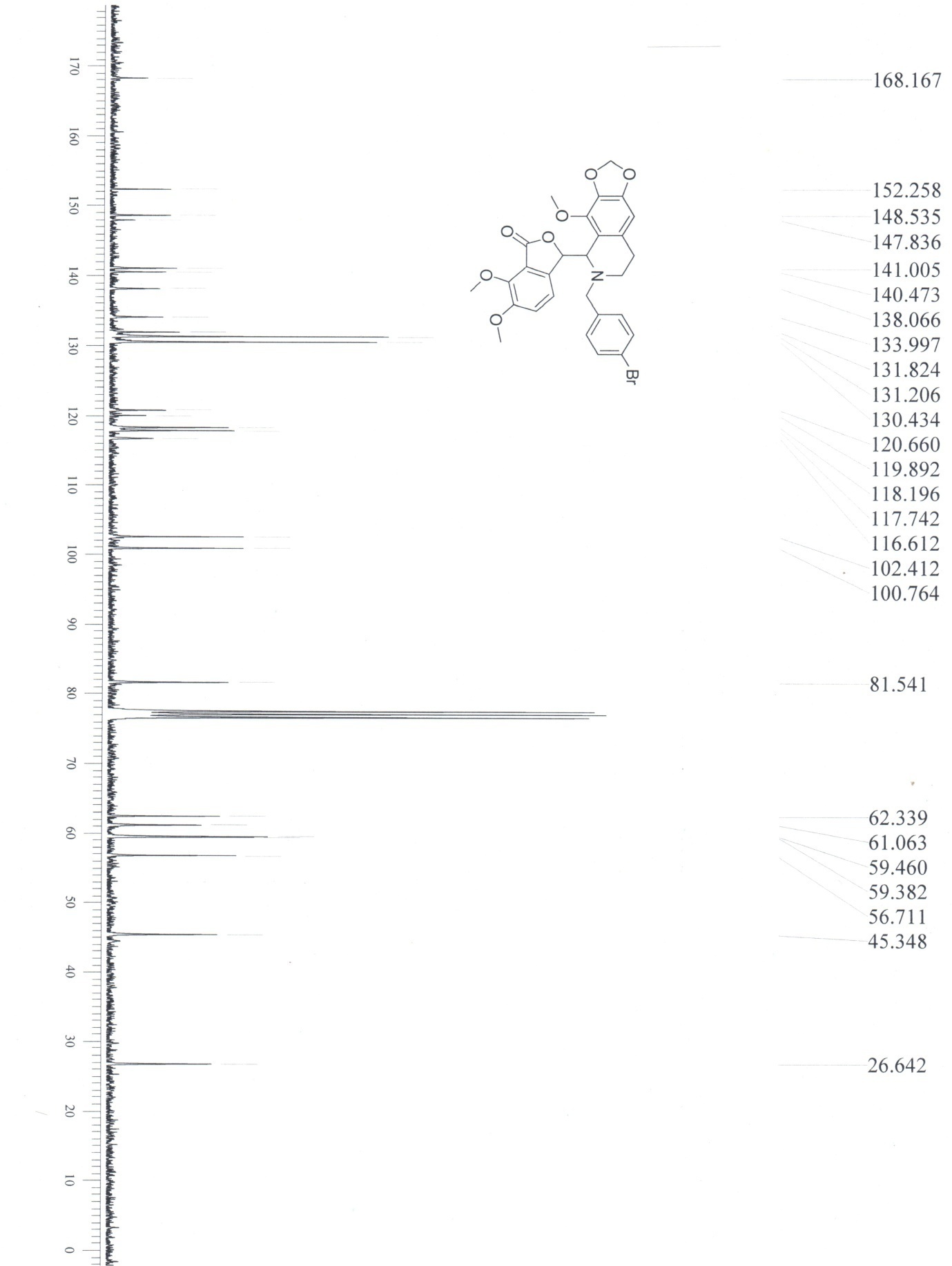 |

| **ESI spectra of 6b.** |
| --- |
| 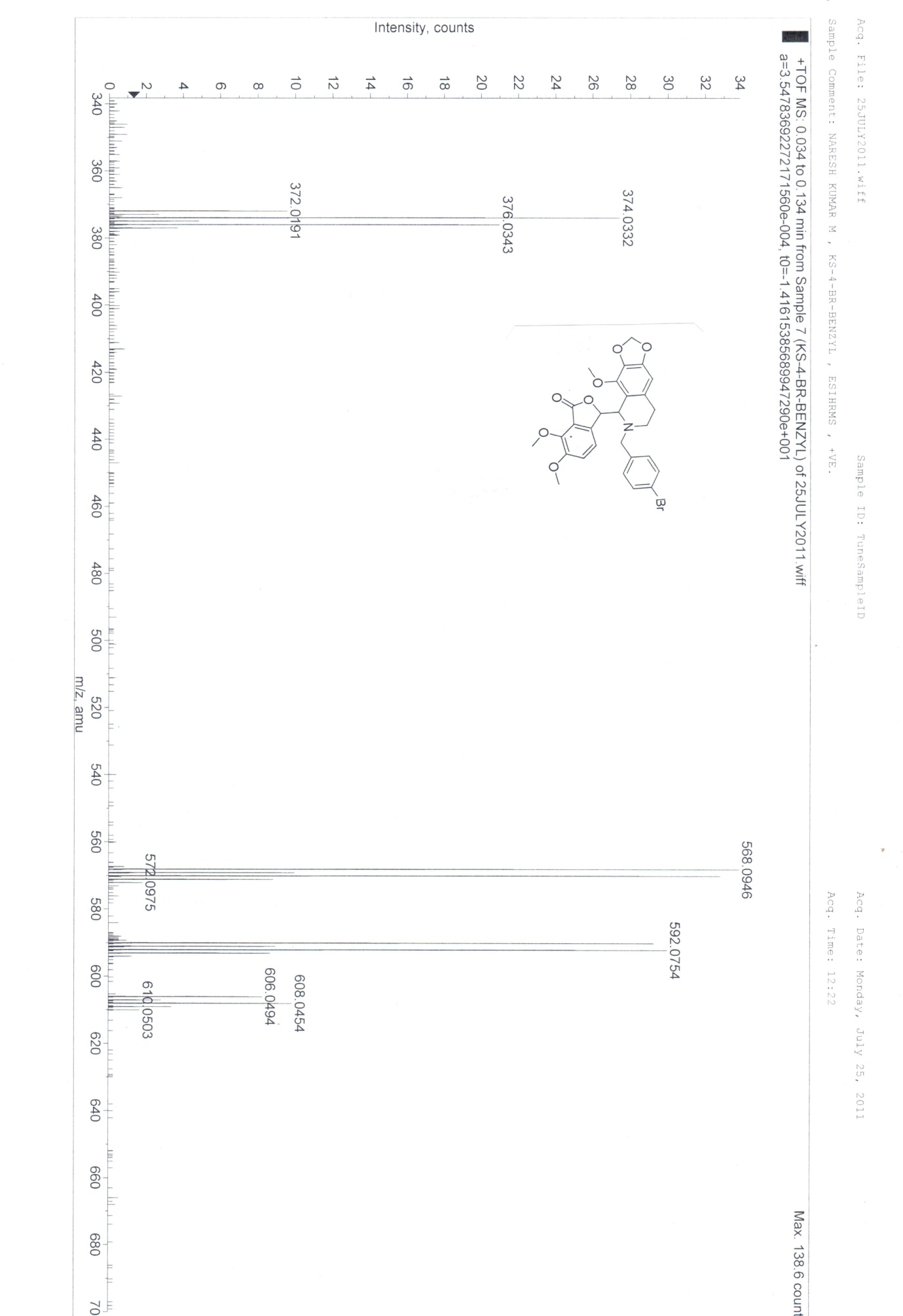 |

| **HRMS spectra of 6b.** |
| --- |
| 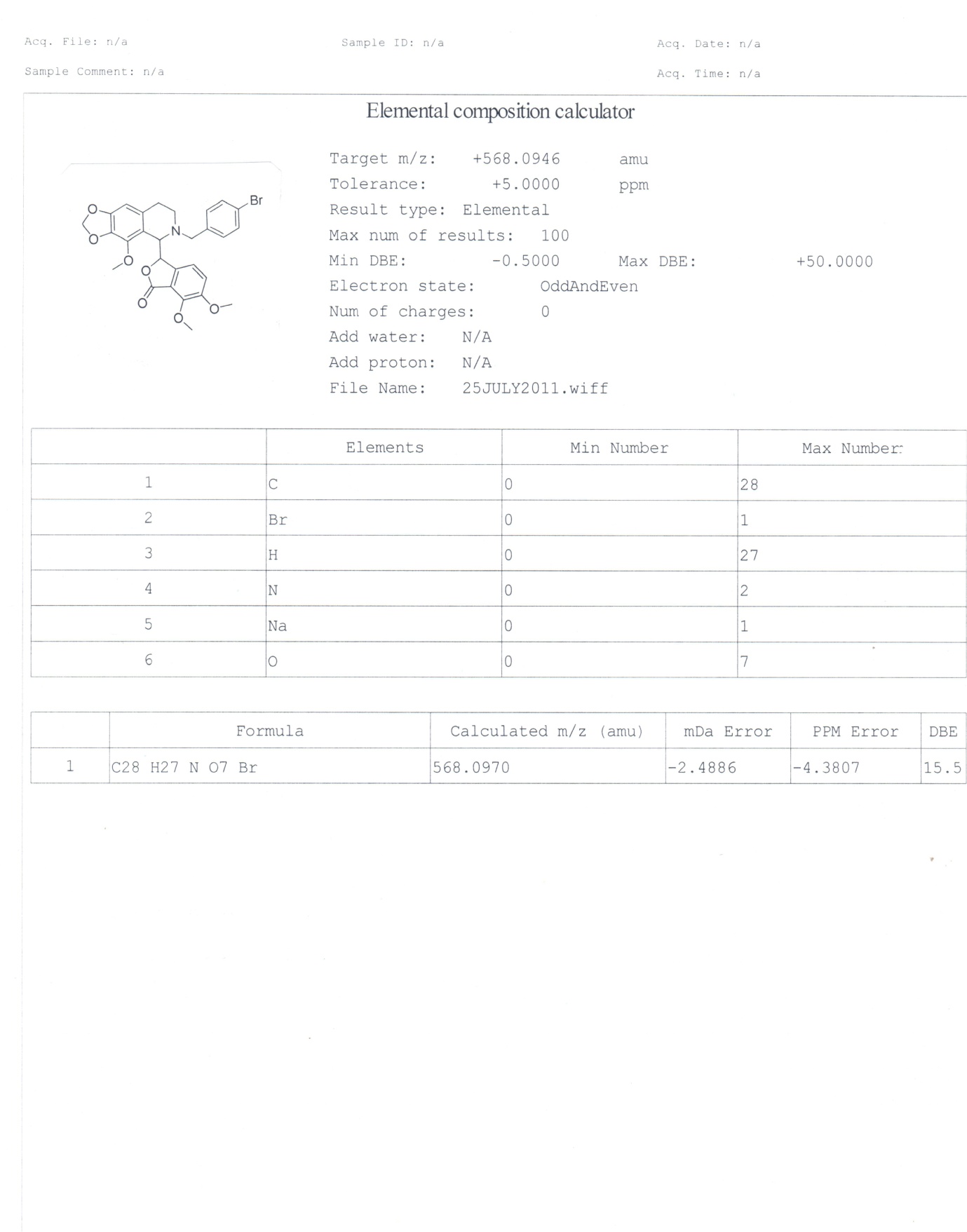 |

| **^1^H NMR spectra of 6c.** |
| --- |
| 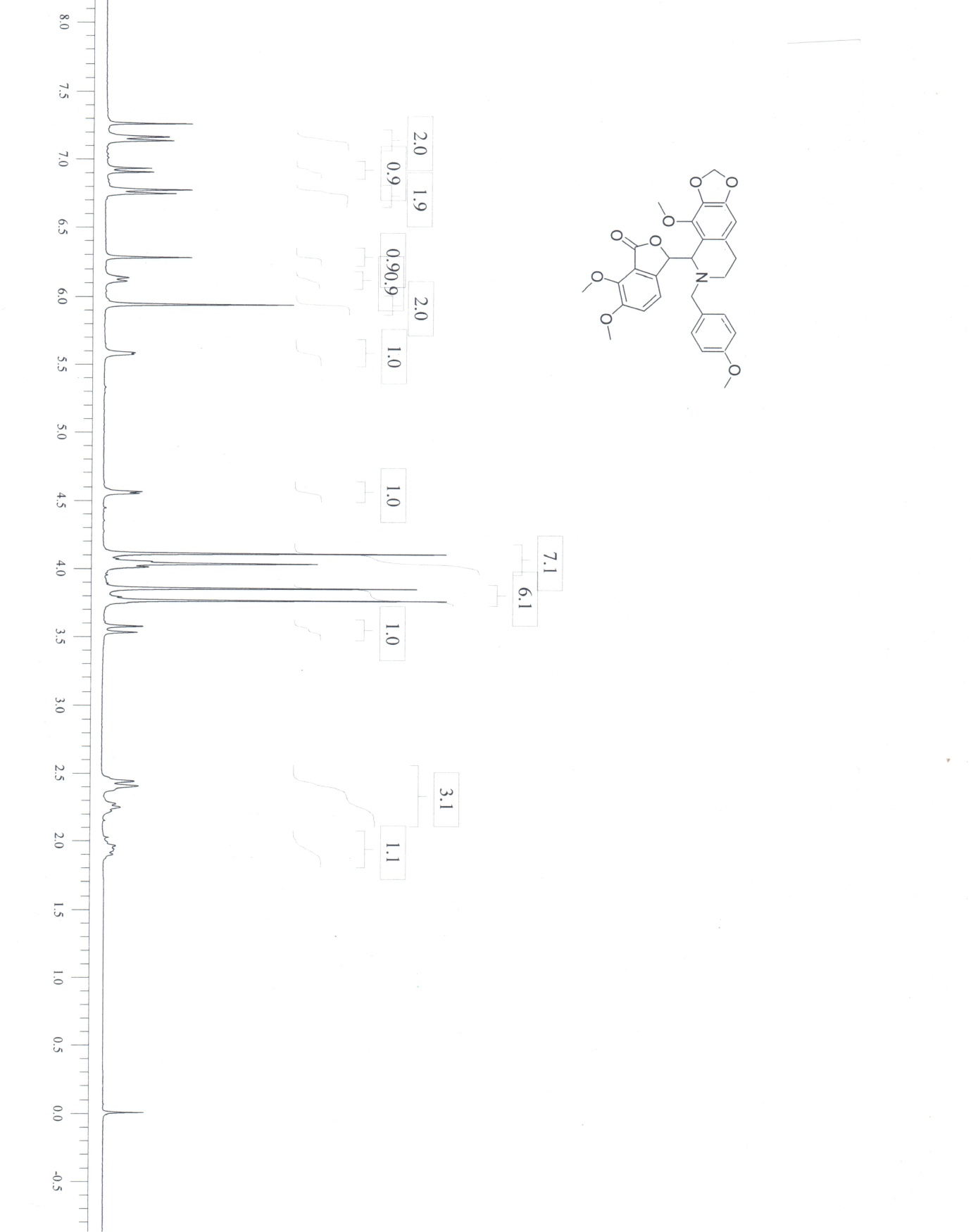 |

| **^13^C NMR spectra of 6c.** |
| --- |
| 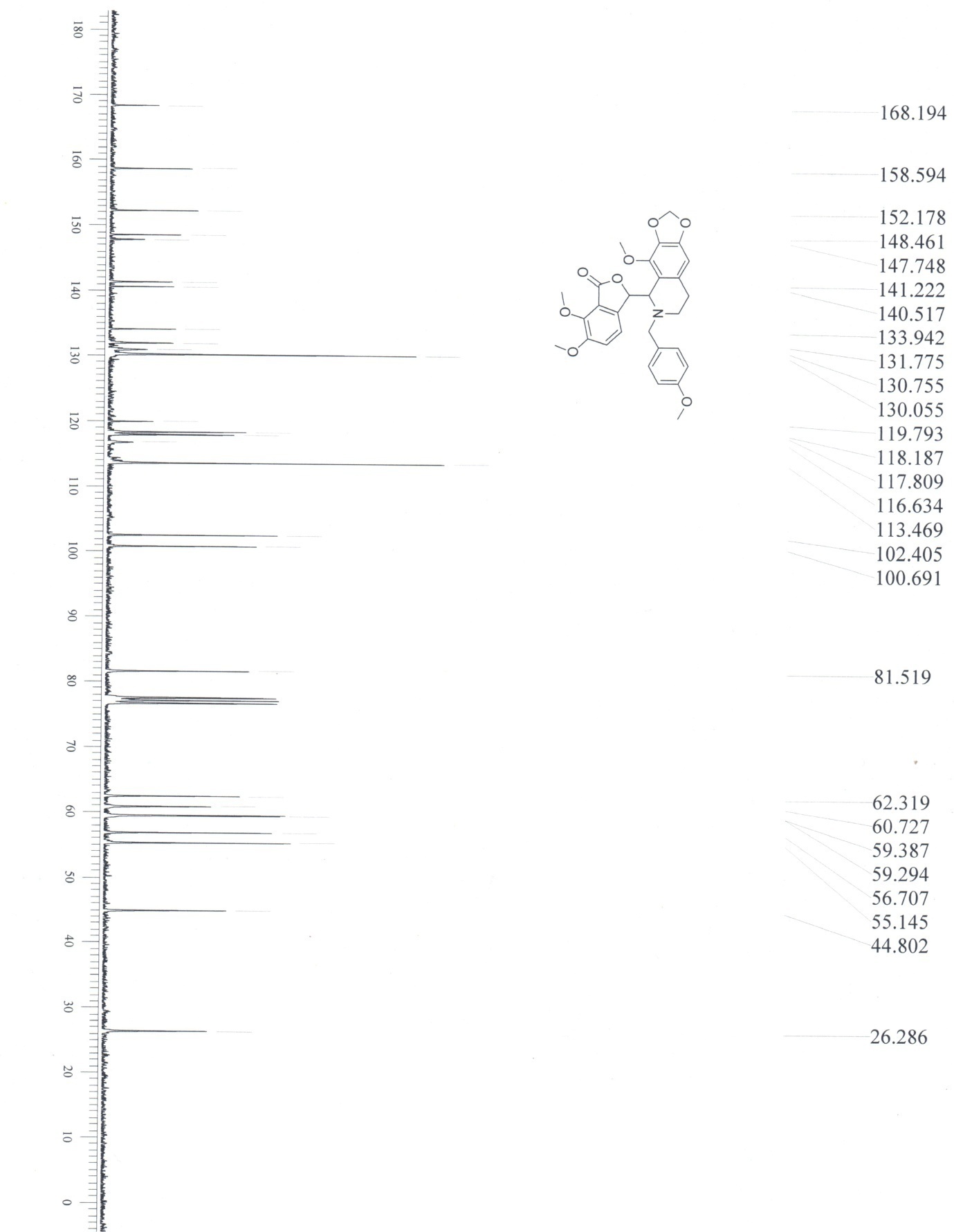 |

| **ESI spectra of 6c.** |
| --- |
| 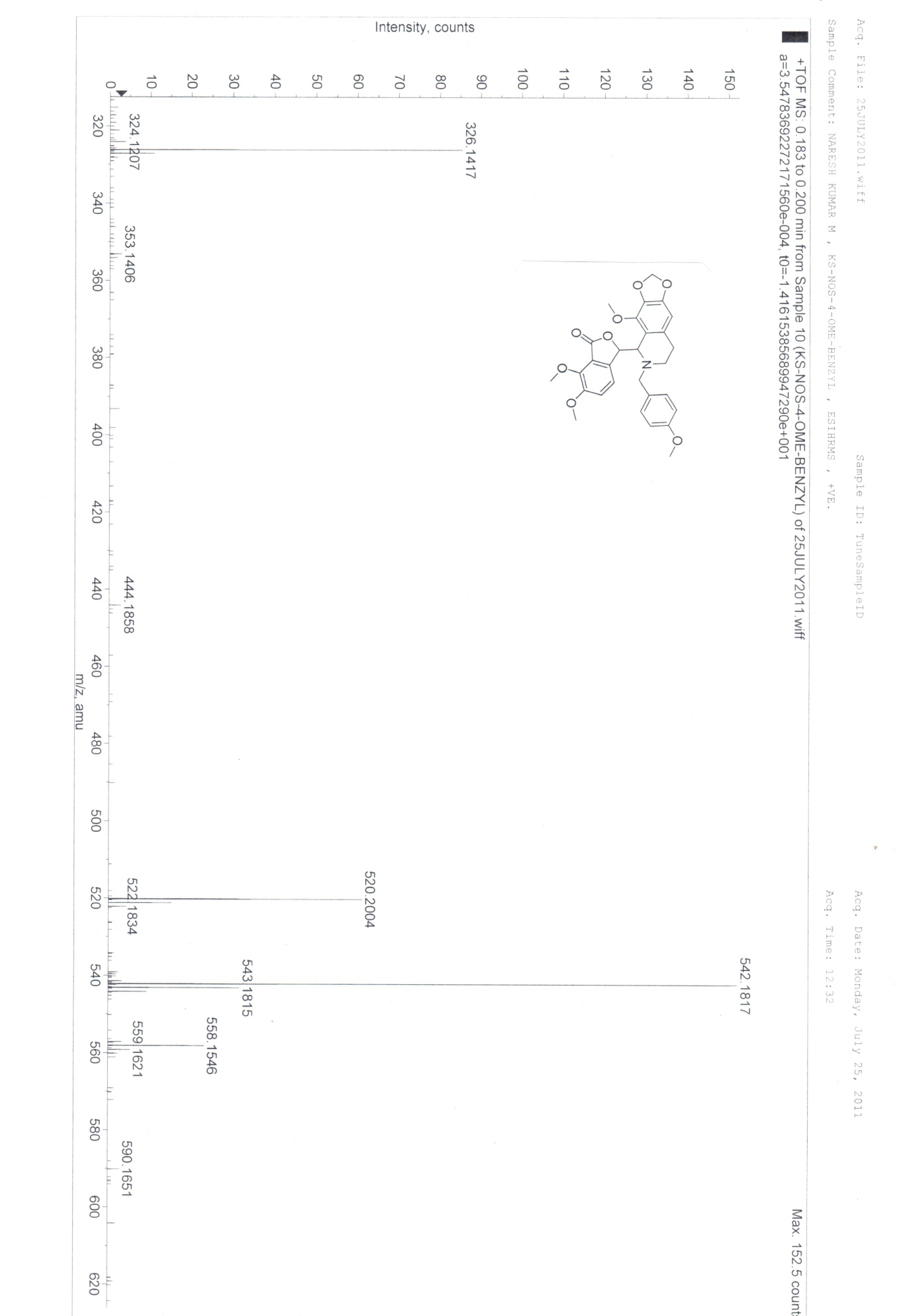 |

| **HRMS spectra of 6c.** |
| --- |
| 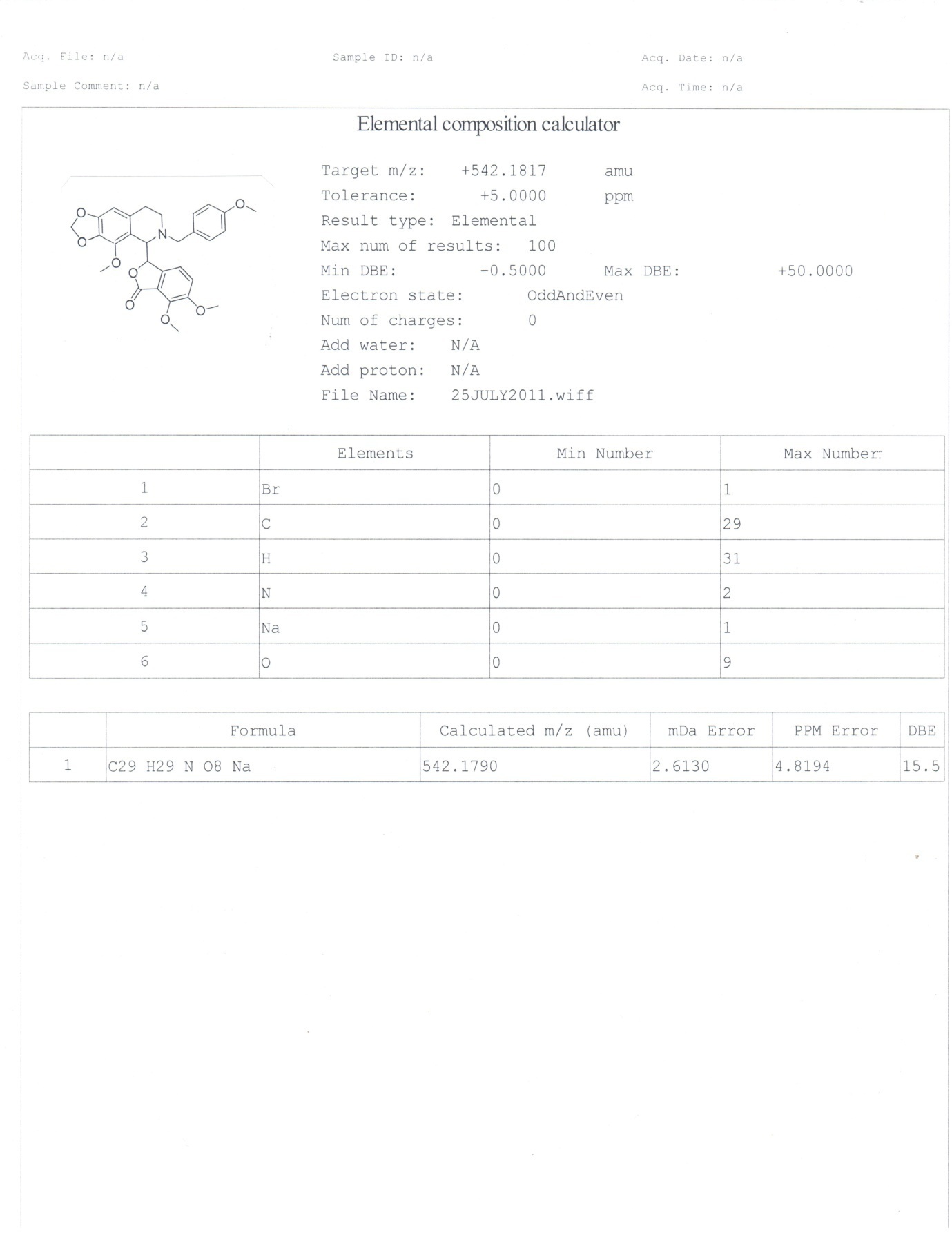 |

| **^1^H NMR spectra of 6d.** |
| --- |
| 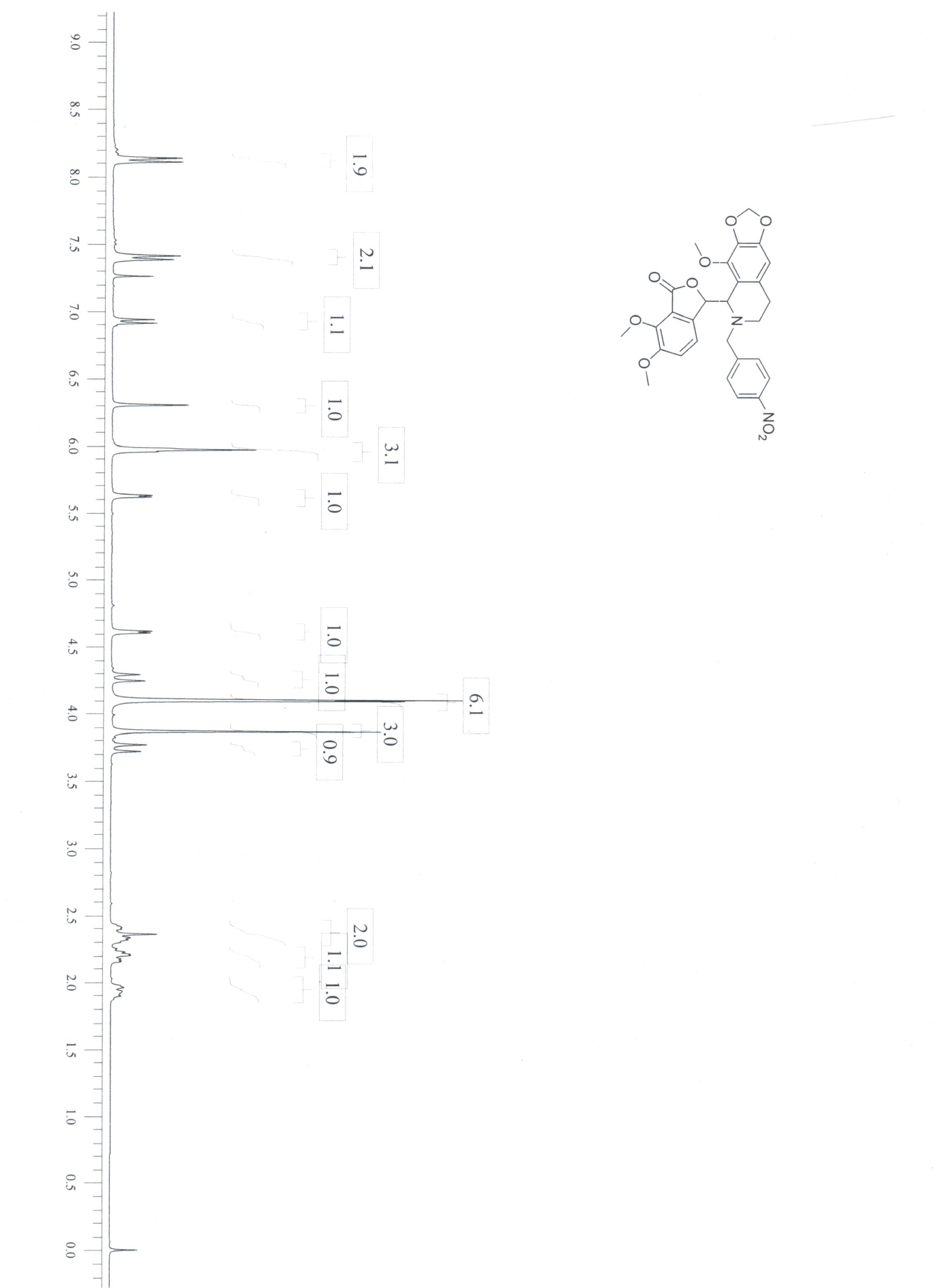 |

| **^13^C NMR spectra of 6d.** |
| --- |
| 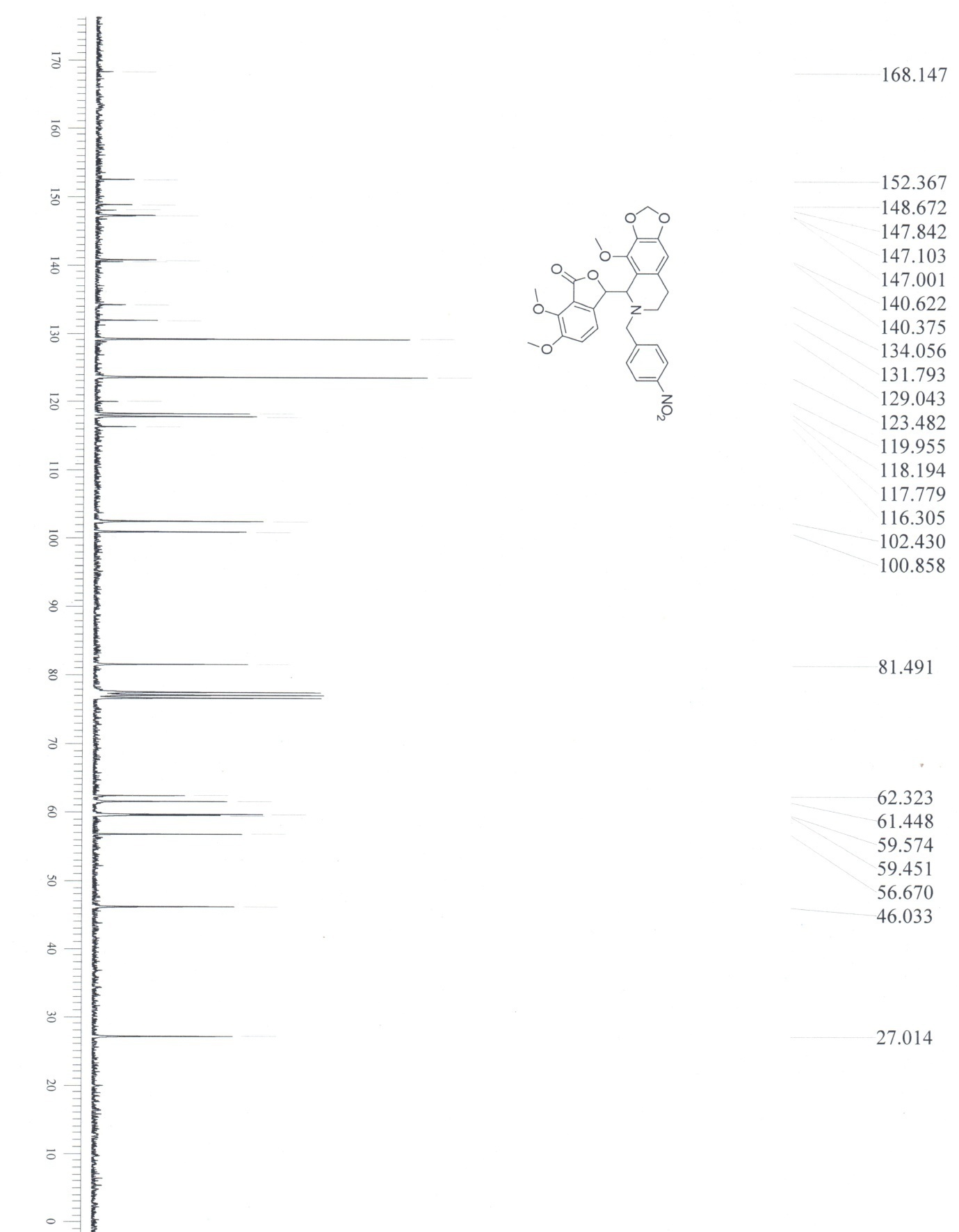 |

| **ESI spectra of 6d.** |
| --- |
| 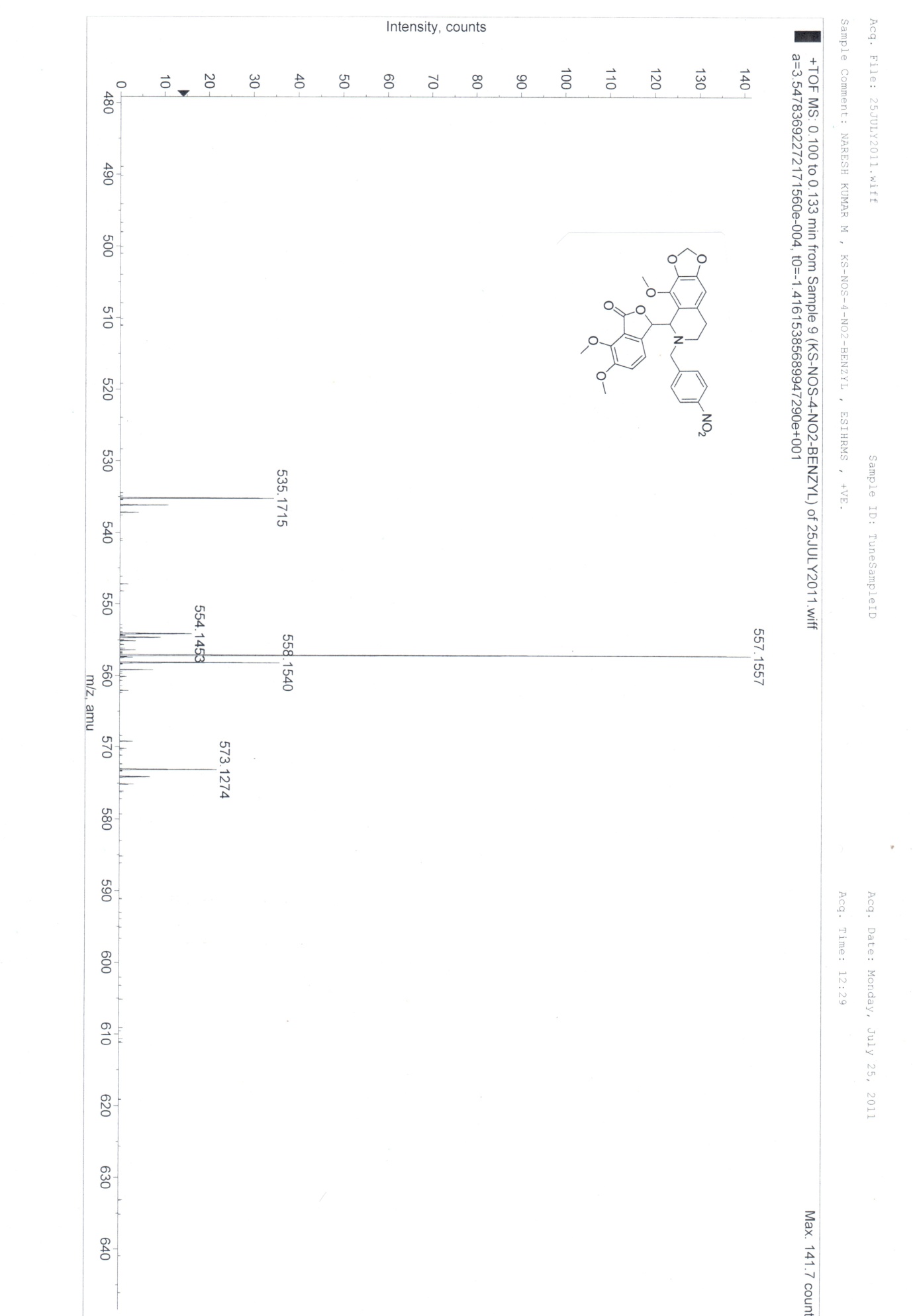 |

| **HRMS spectra of 6d.** |
| --- |
| 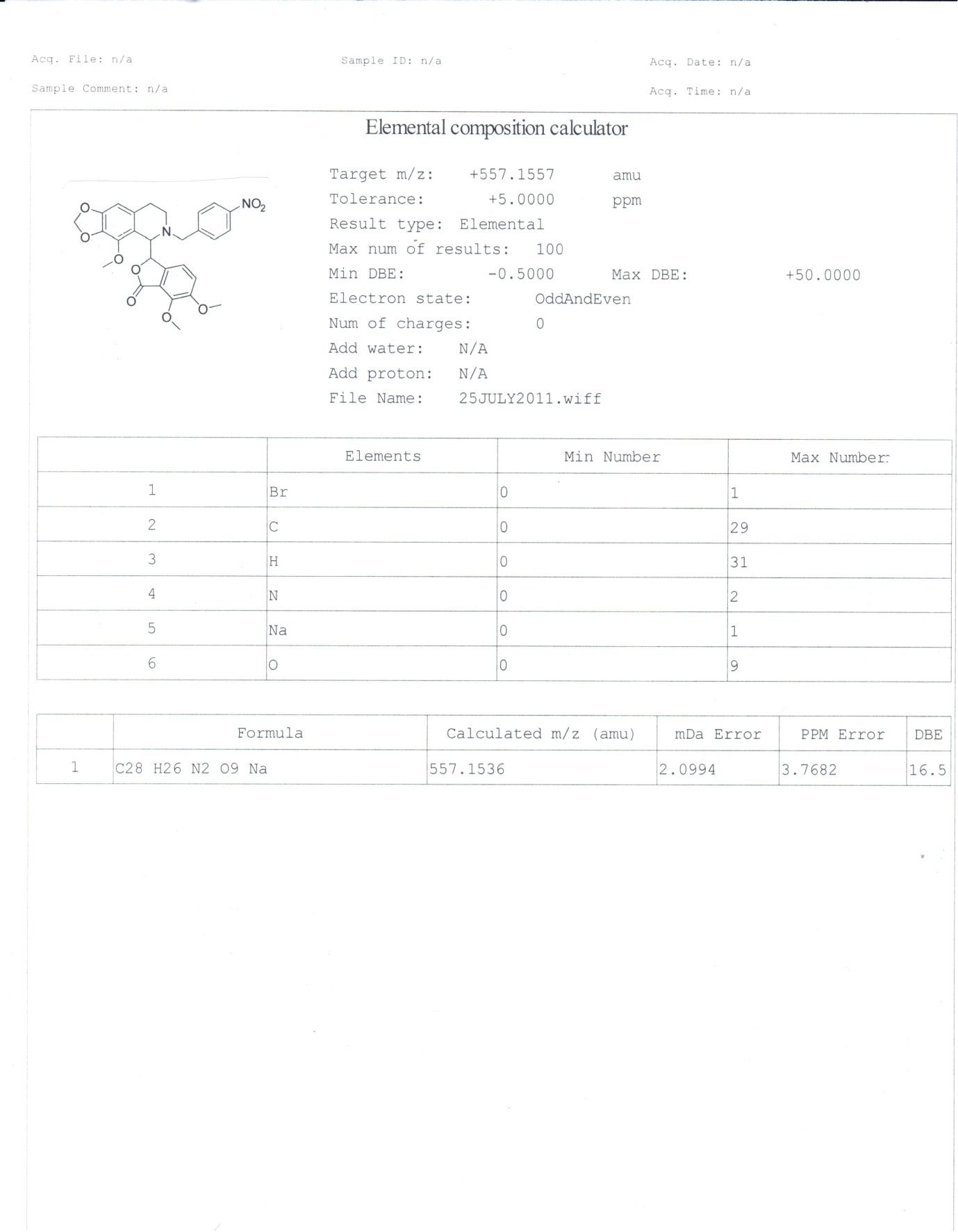 |

| **^1^H spectra of 6e.** |
| --- |
| 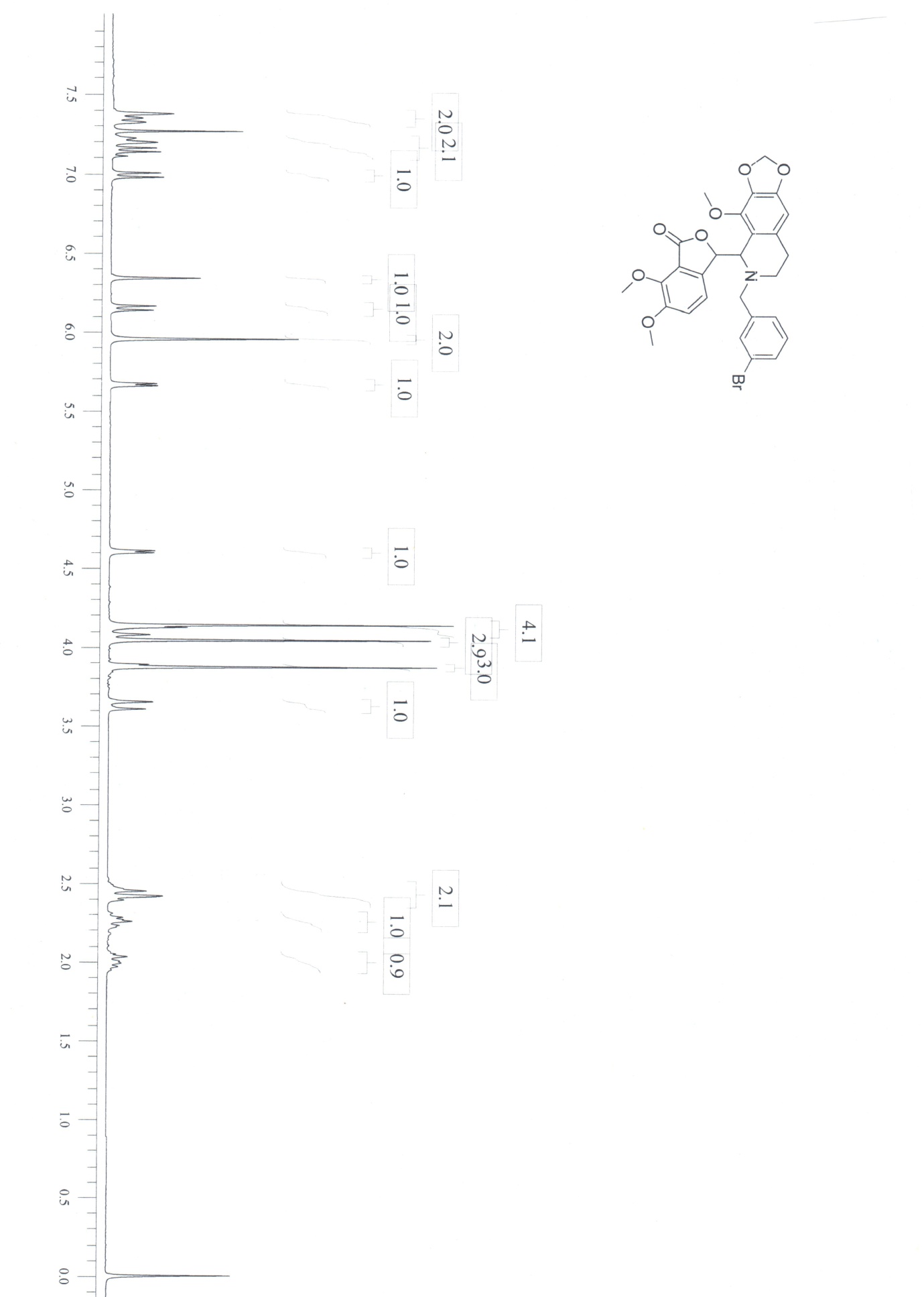 |

| **^13^C NMR spectra of 6e.** |
| --- |
| 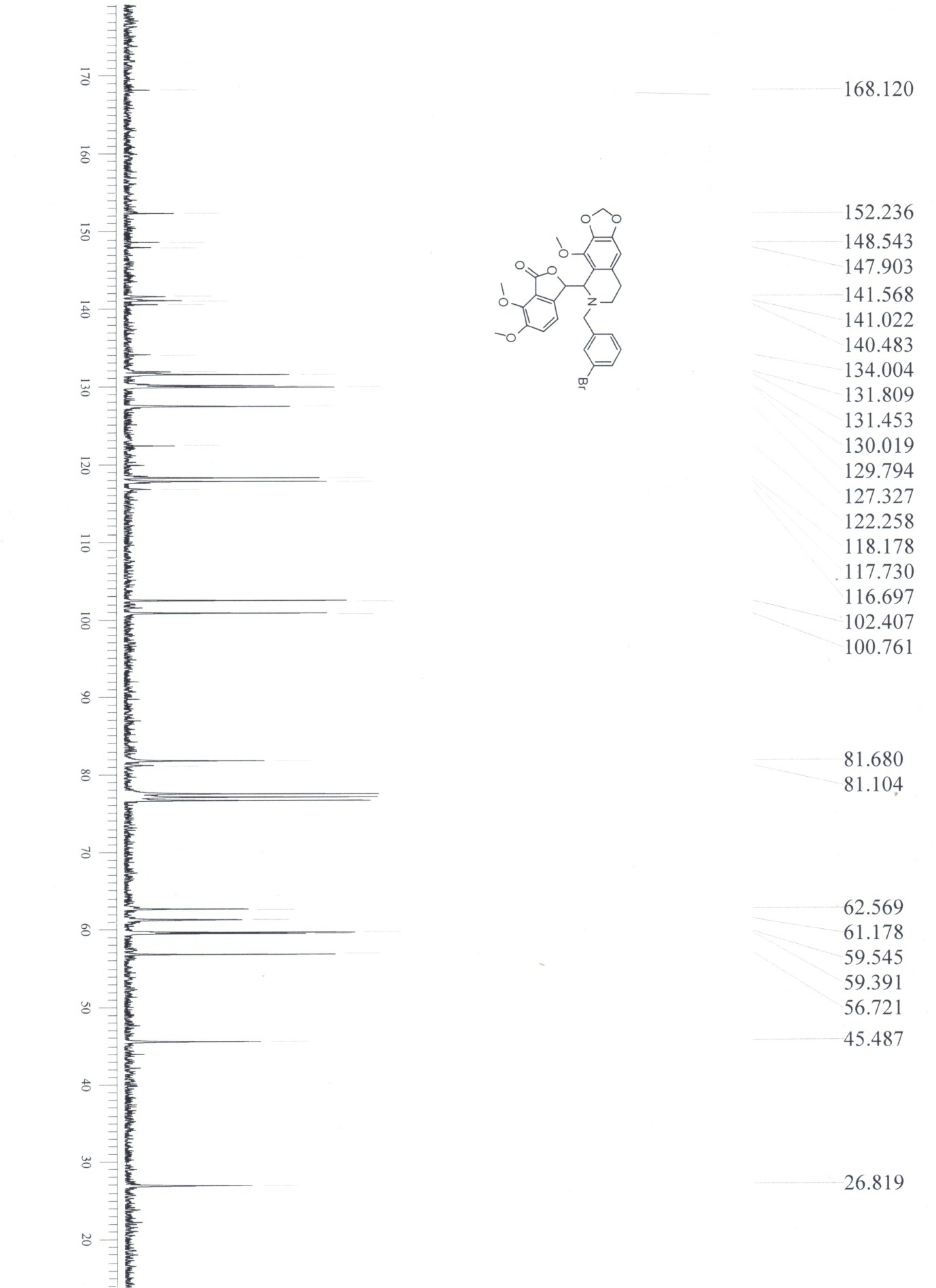 |

| **ESI spectra of 6e.** |
| --- |
| 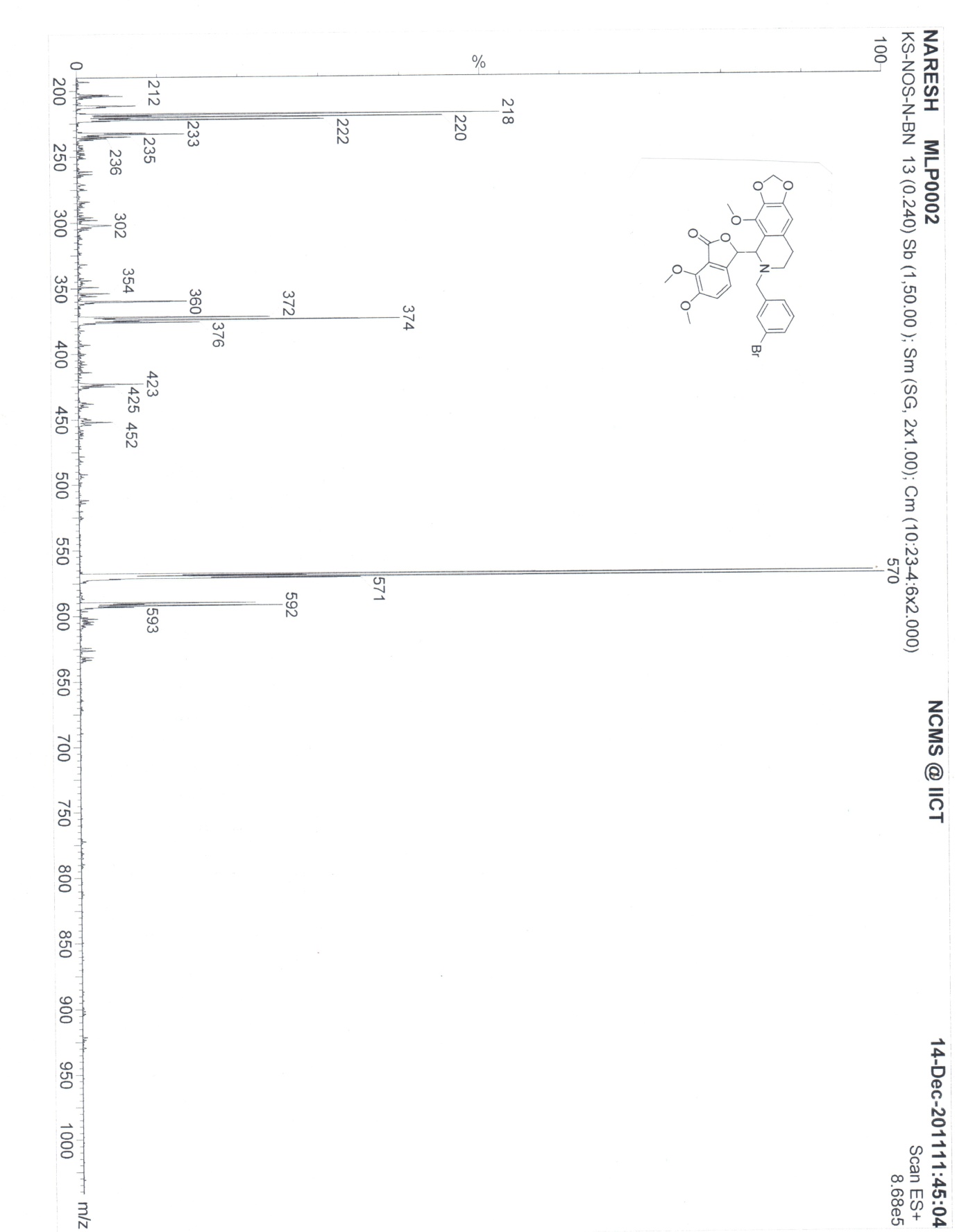 |

| **^1^H NMR spectra of 6f.** |
| --- |
| 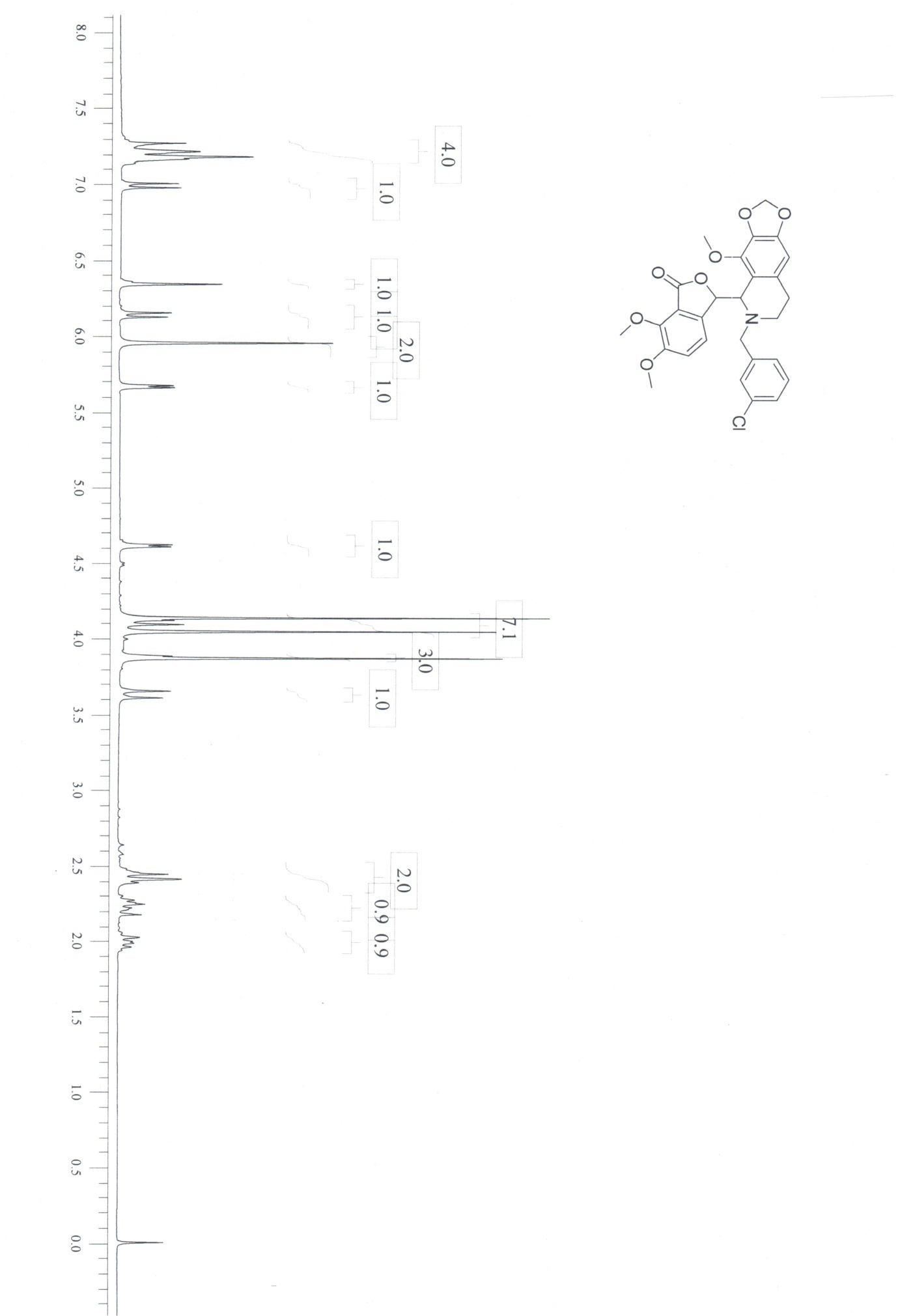 |

| **^13^C NMR spectra of 6f.** |
| --- |
| 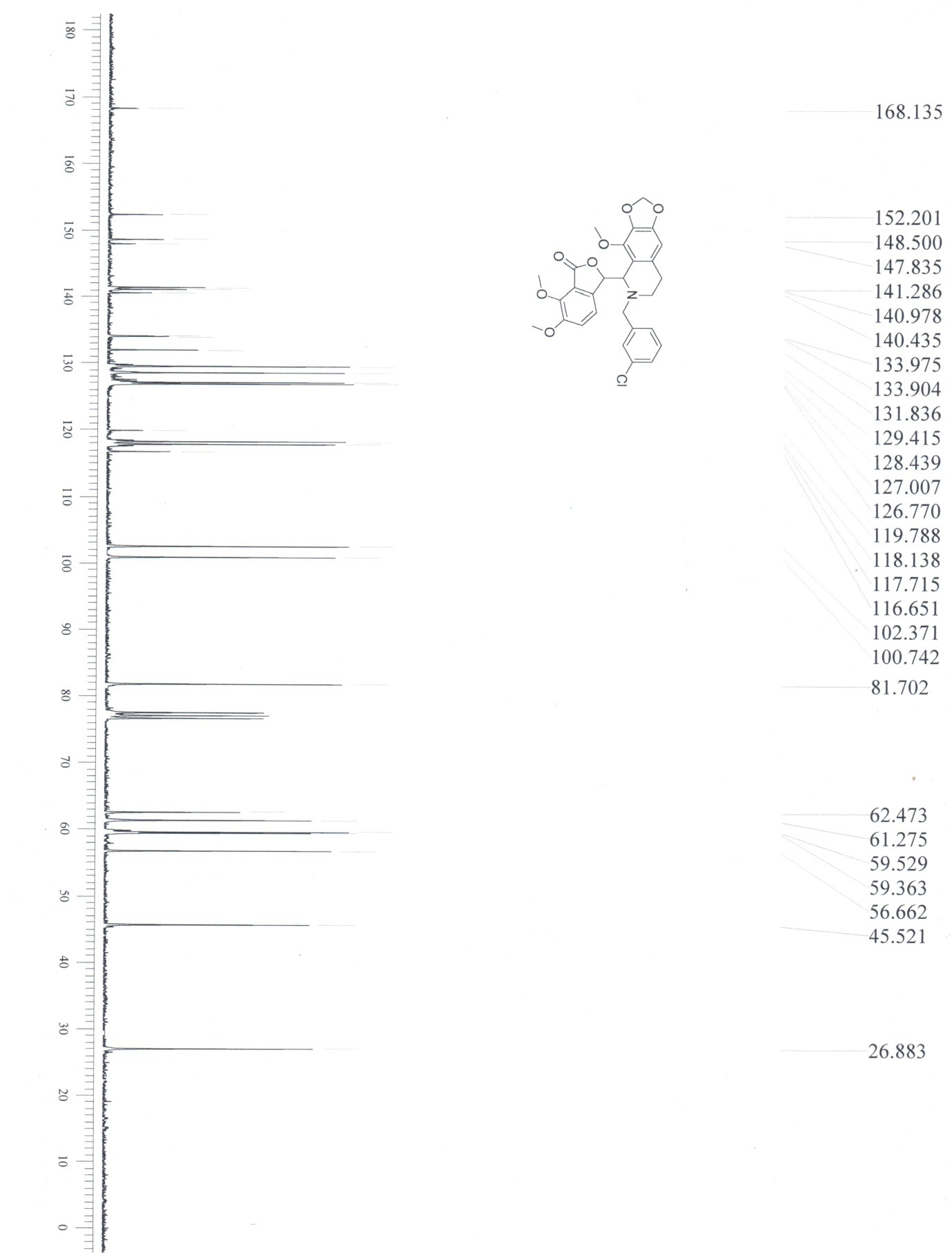 |

| **ESI spectra of 6f.** |
| --- |
| 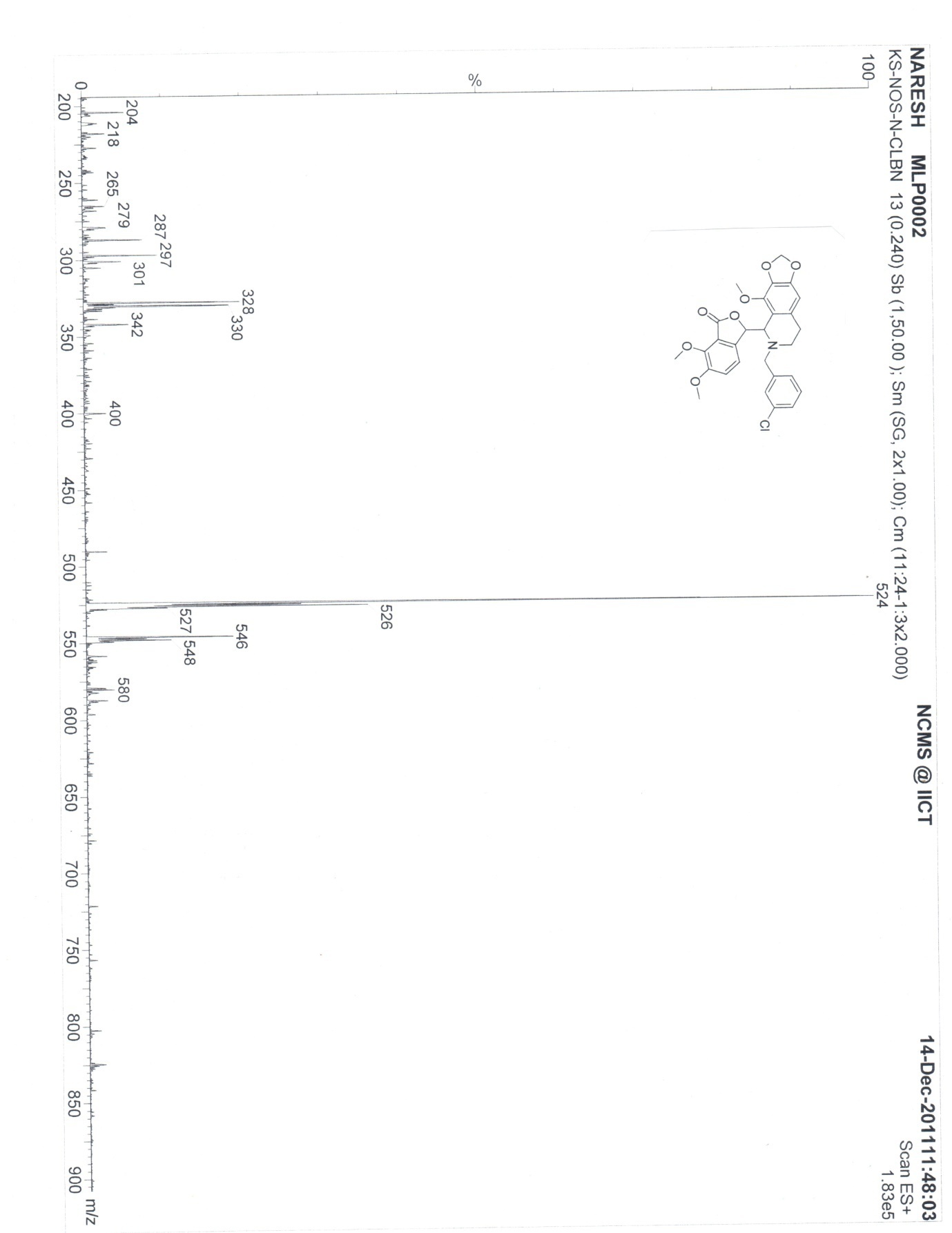 |

| **^1^H NMR spectra of 6g.** |
| --- |
| 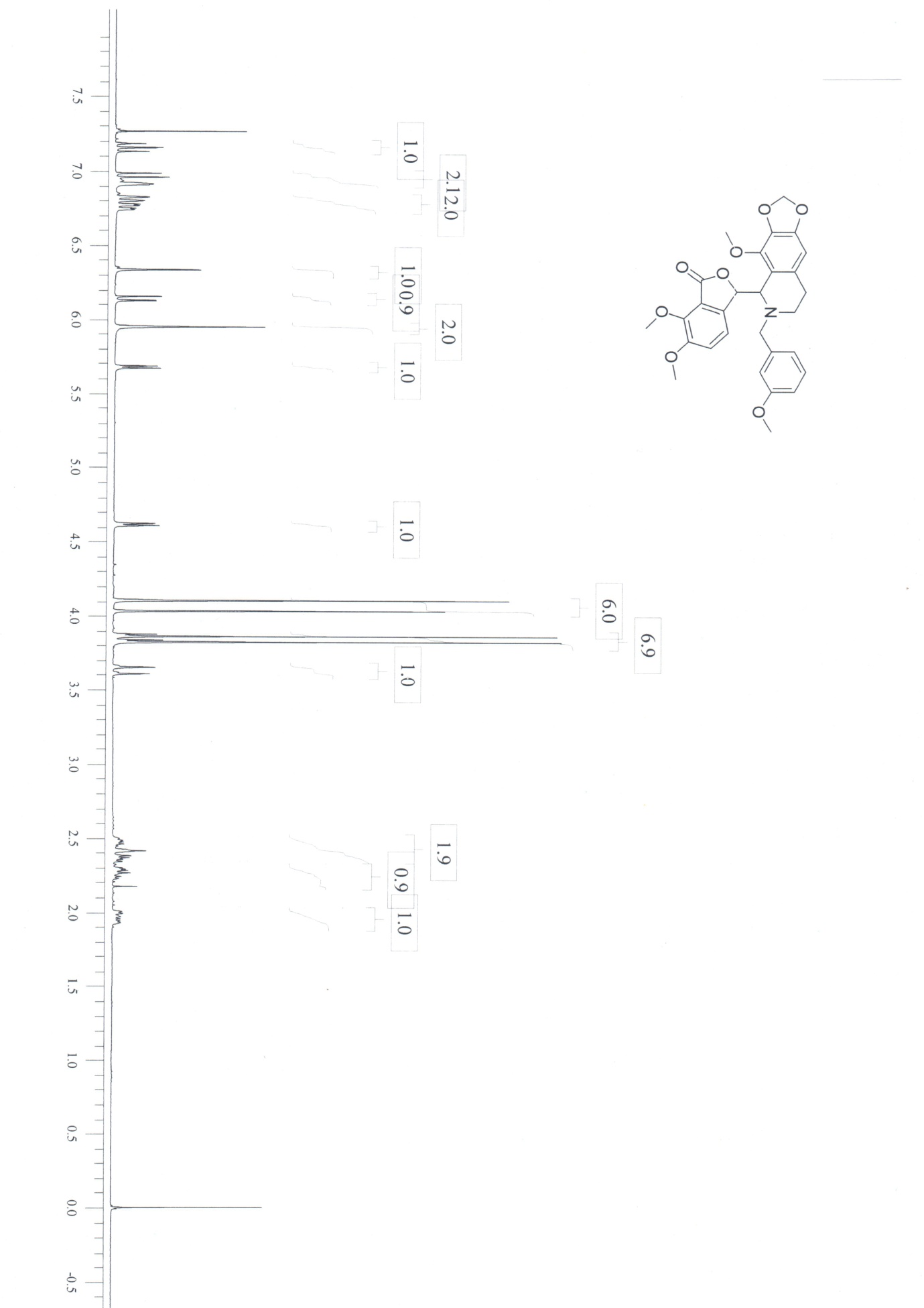 |

| **^13^C NMR of 6g.** |
| --- |
| 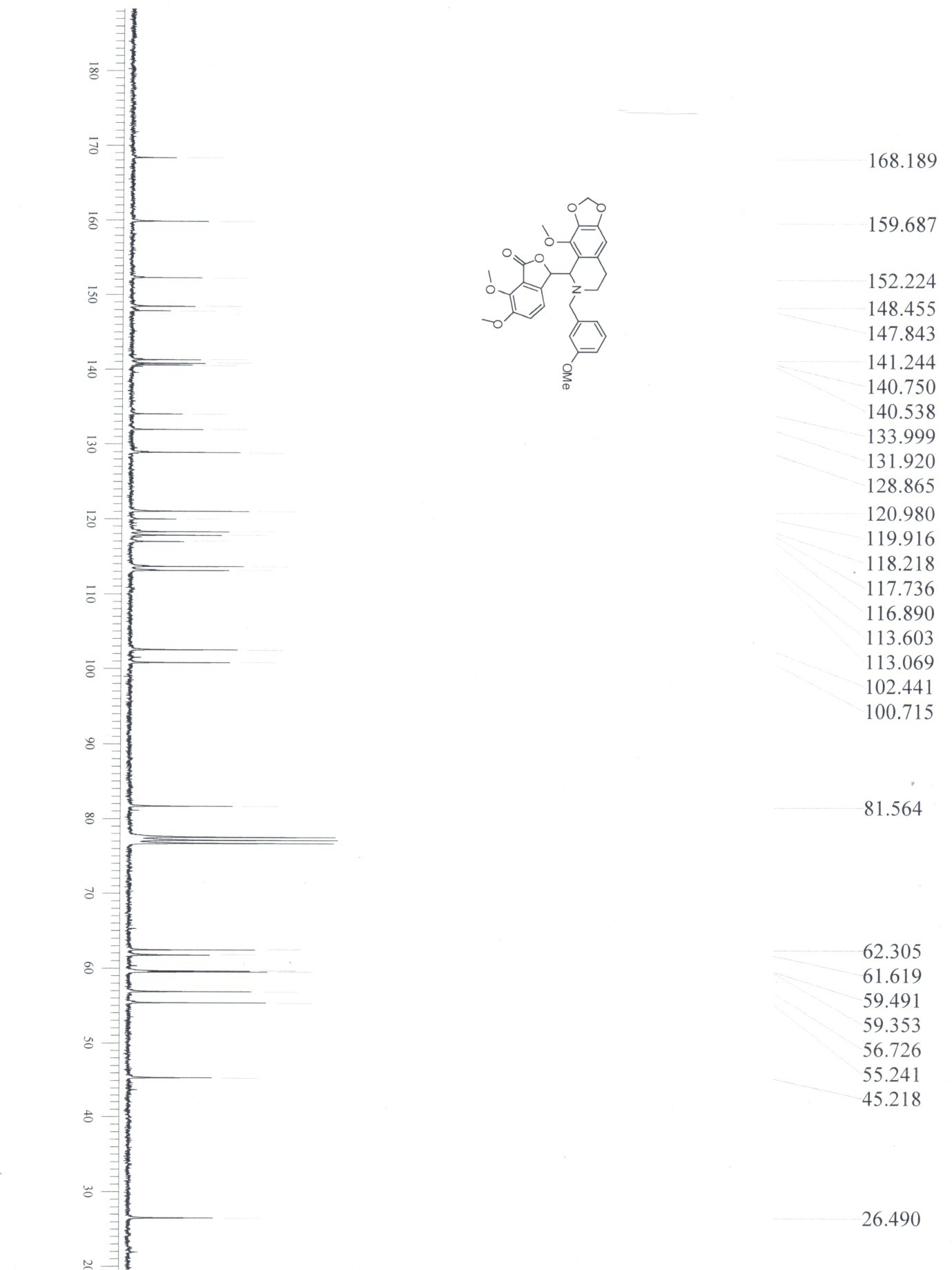 |

| **ESI spectra of 6g.** |
| --- |
| 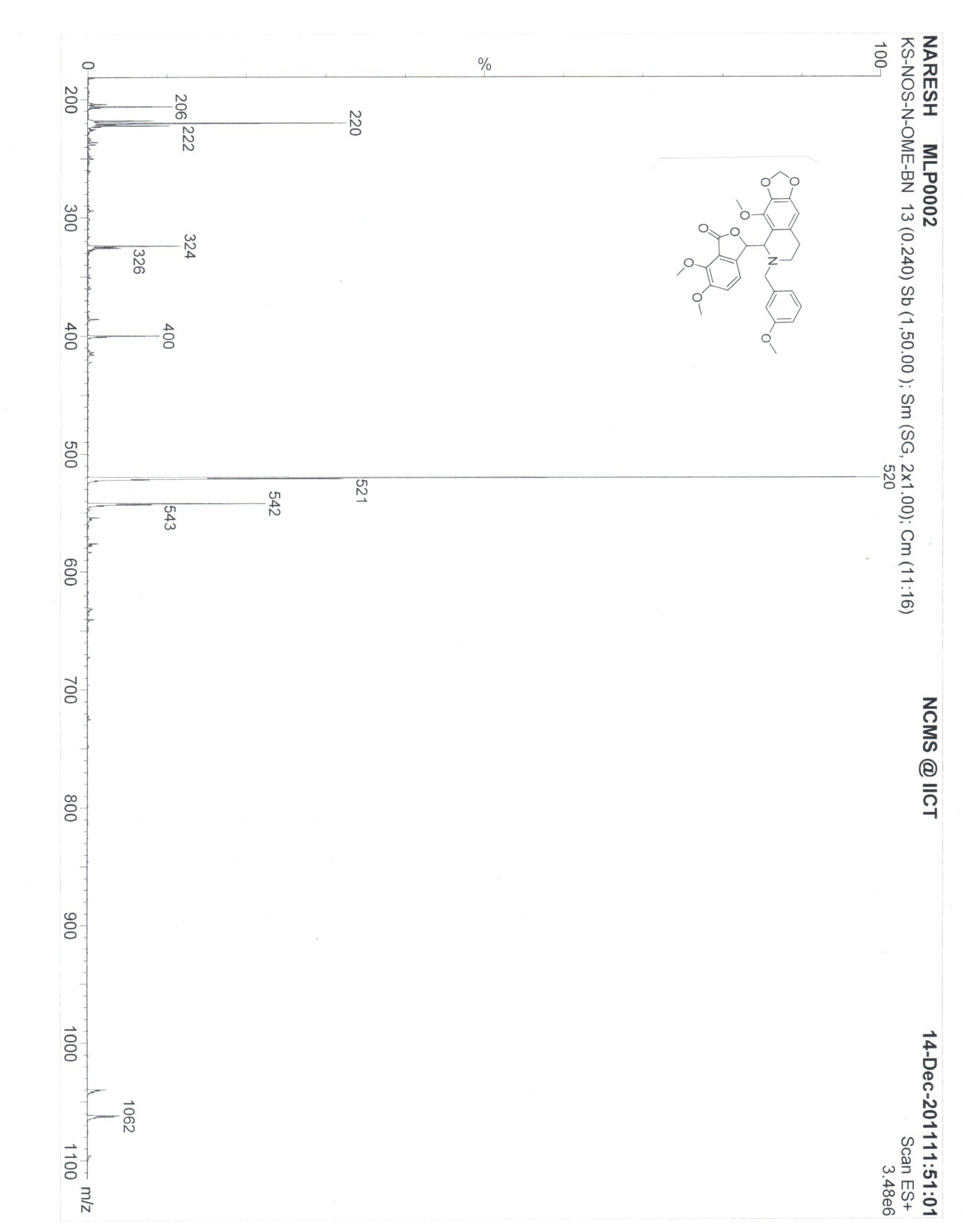 |

| **^1^H NMR spectra of 6h.** |
| --- |
| 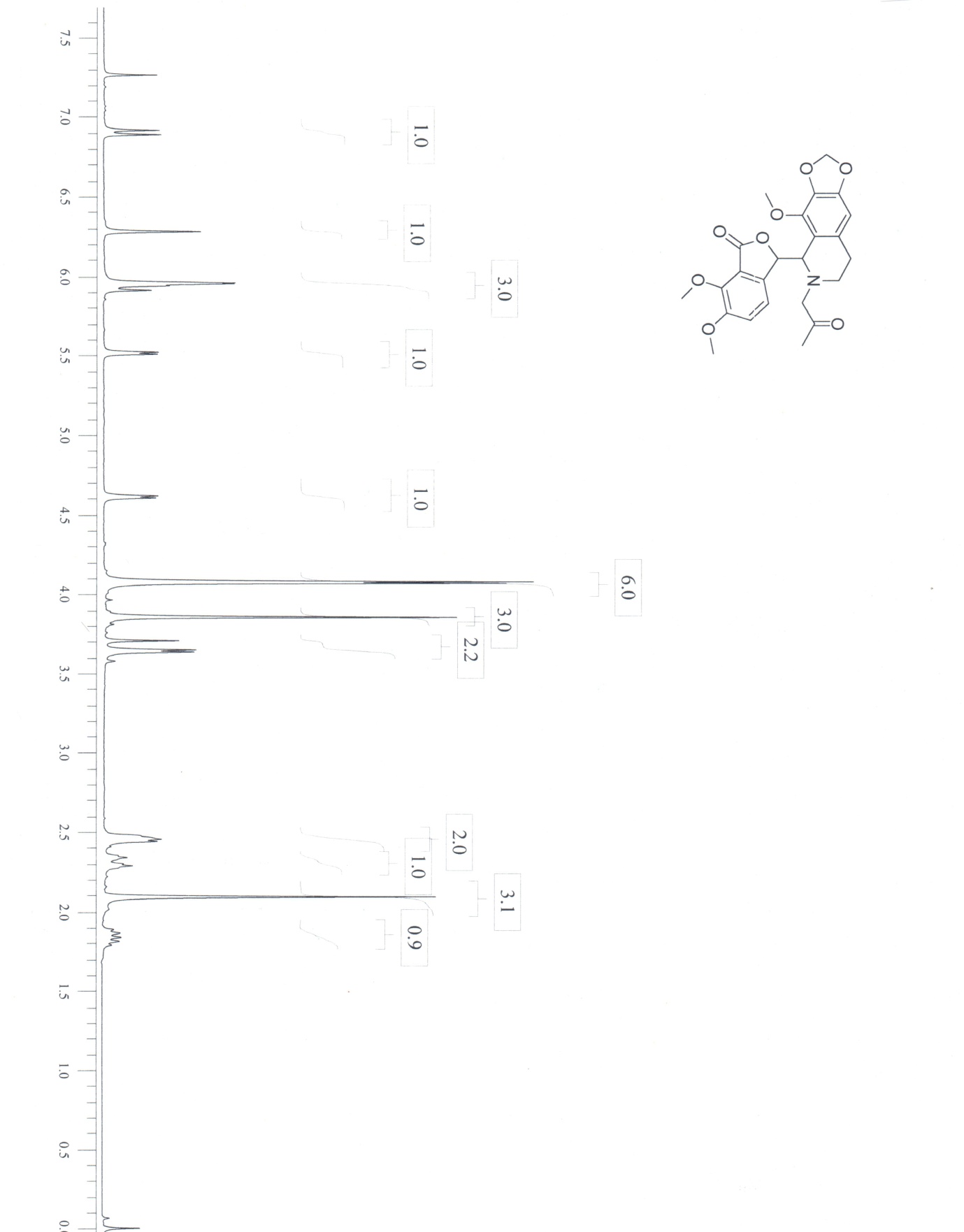 |

| **^13^C NMR spectra of 6h.** |
| --- |
| 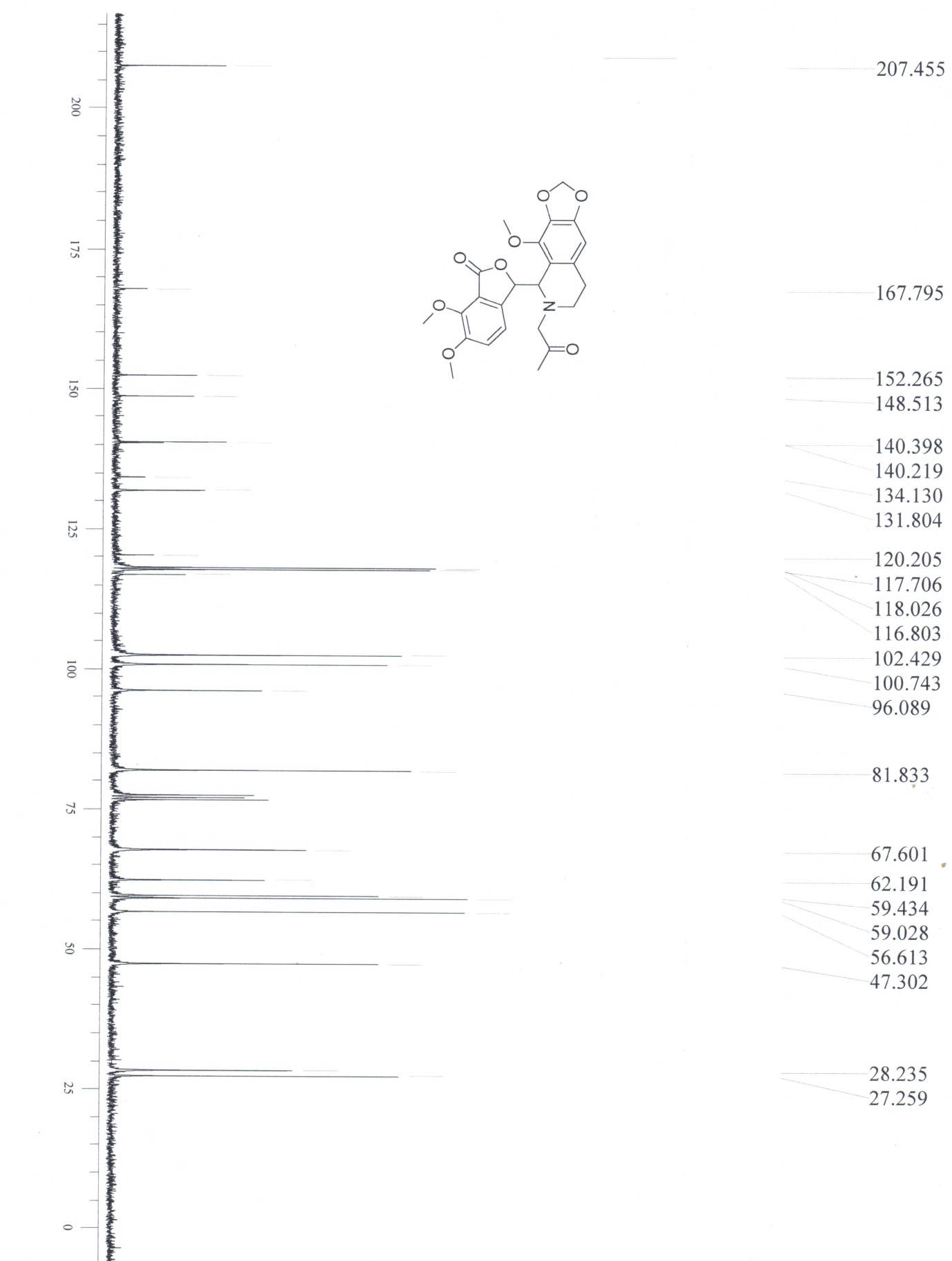 |

| **ESI spectra of 6h.** |
| --- |
| 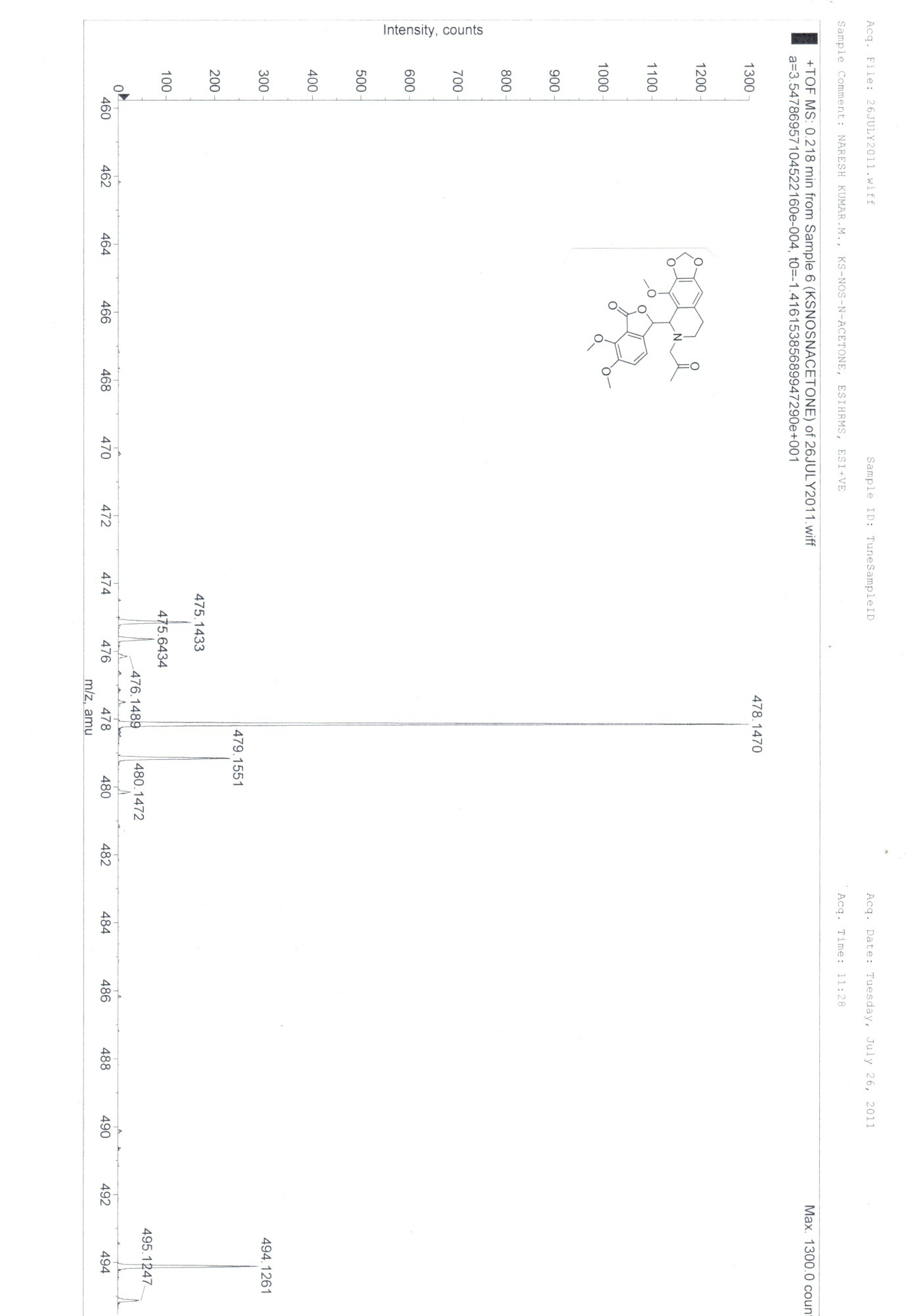 |

| **HRMS spectra of 6h.** |
| --- |
| 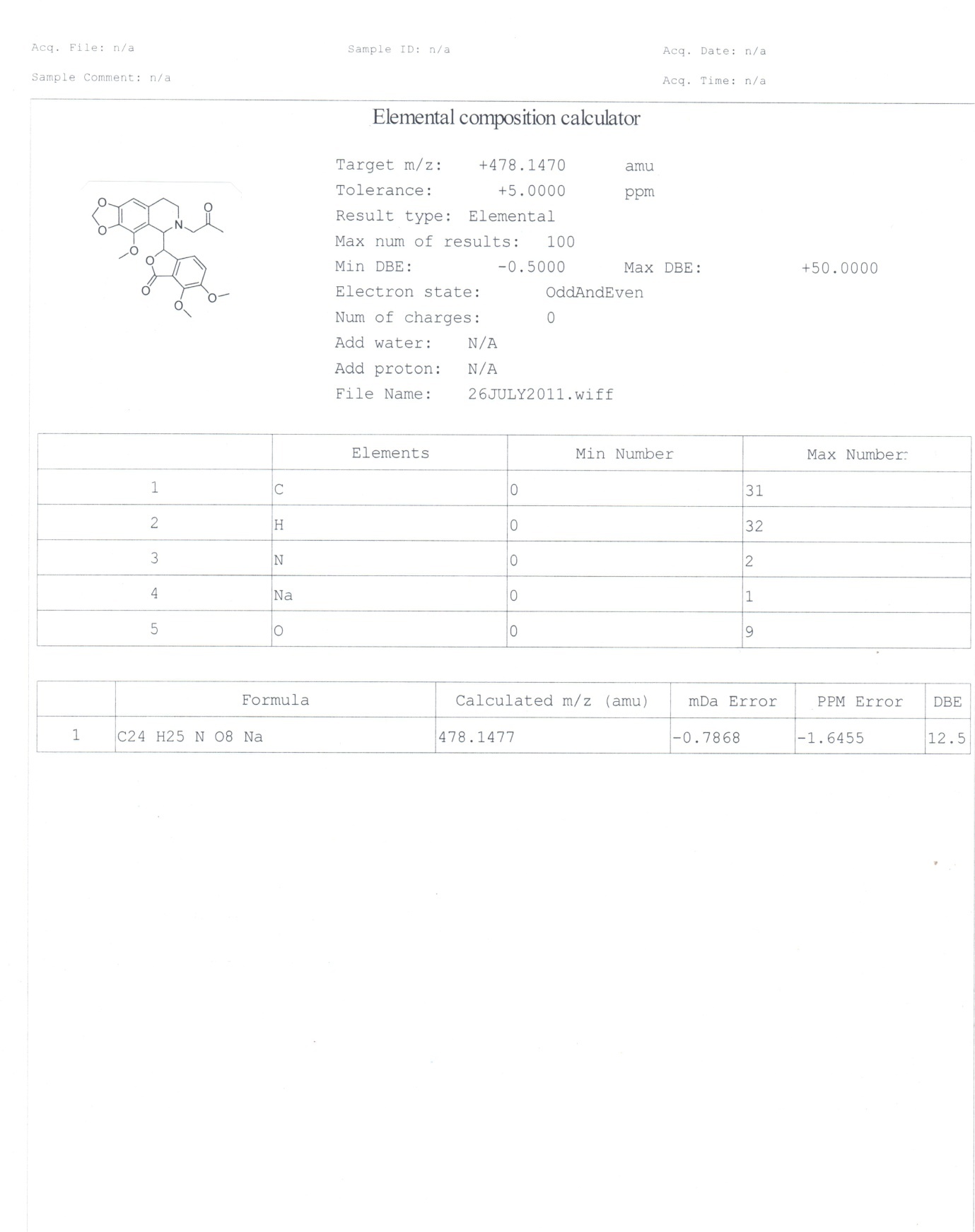 |

| **^1^H NMR spectra of 6i.** |
| --- |
| 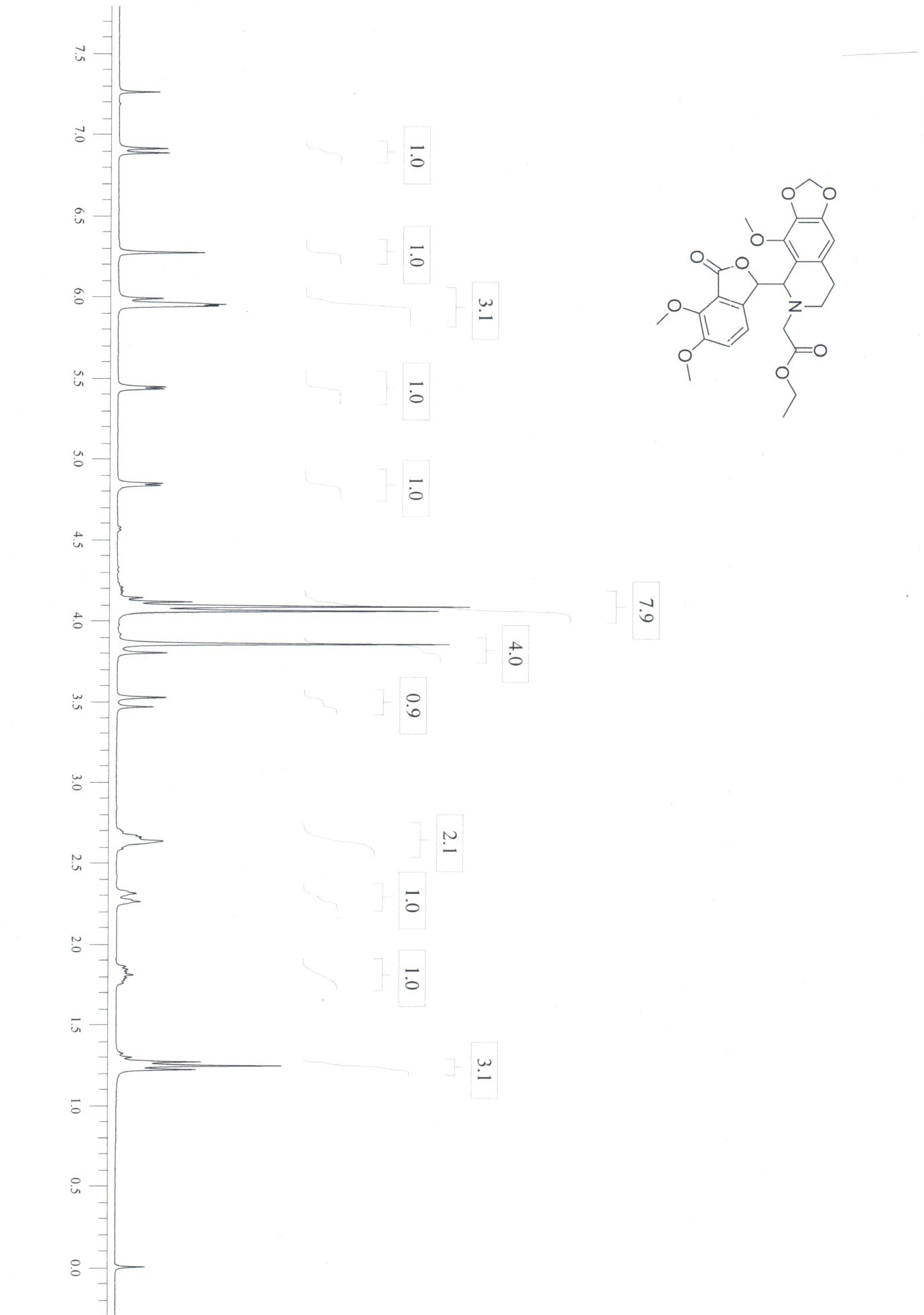 |

| **^13^C NMR spectra of 6i.** |
| --- |
| 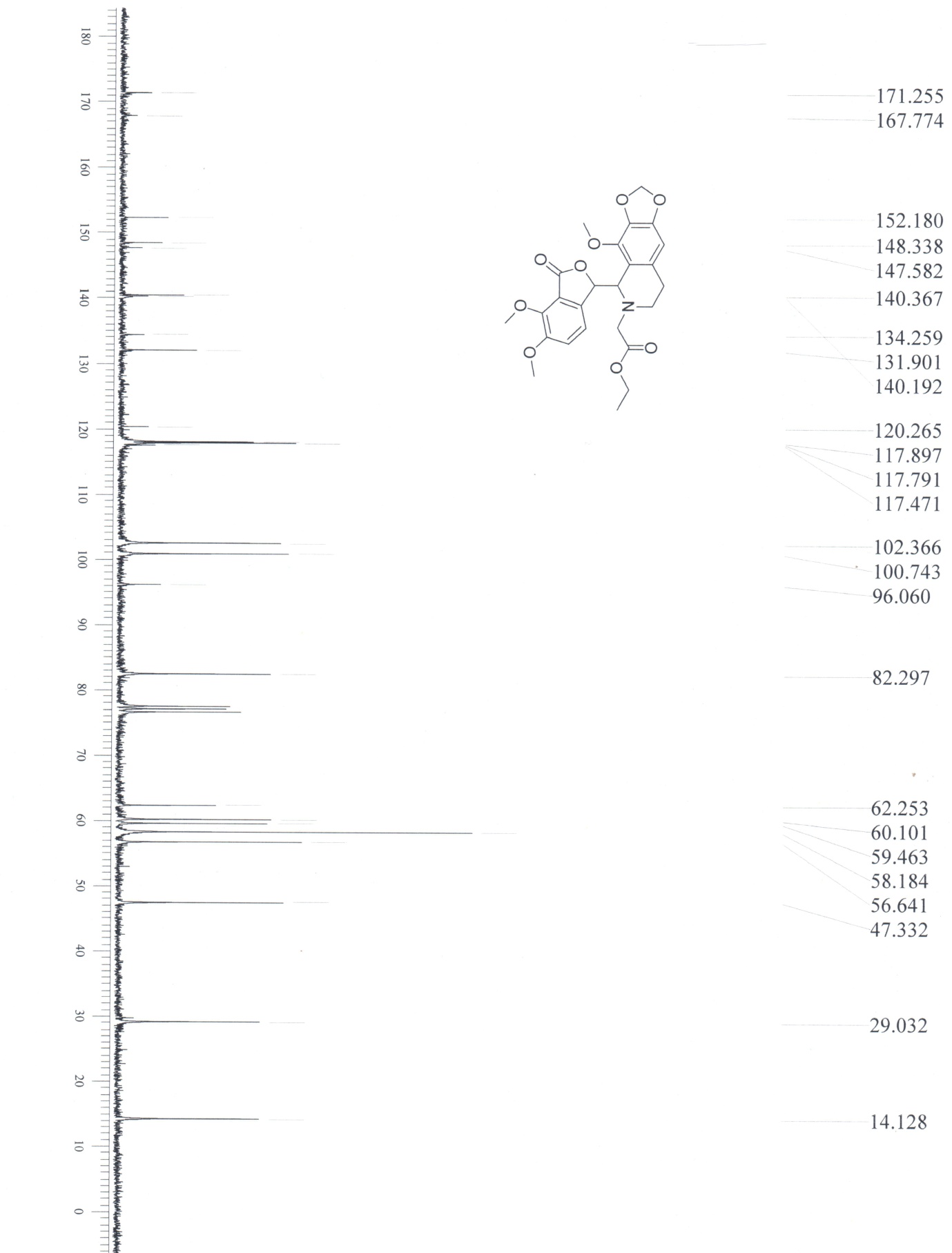 |

| **ESI spectra of 6i.** |
| --- |
| 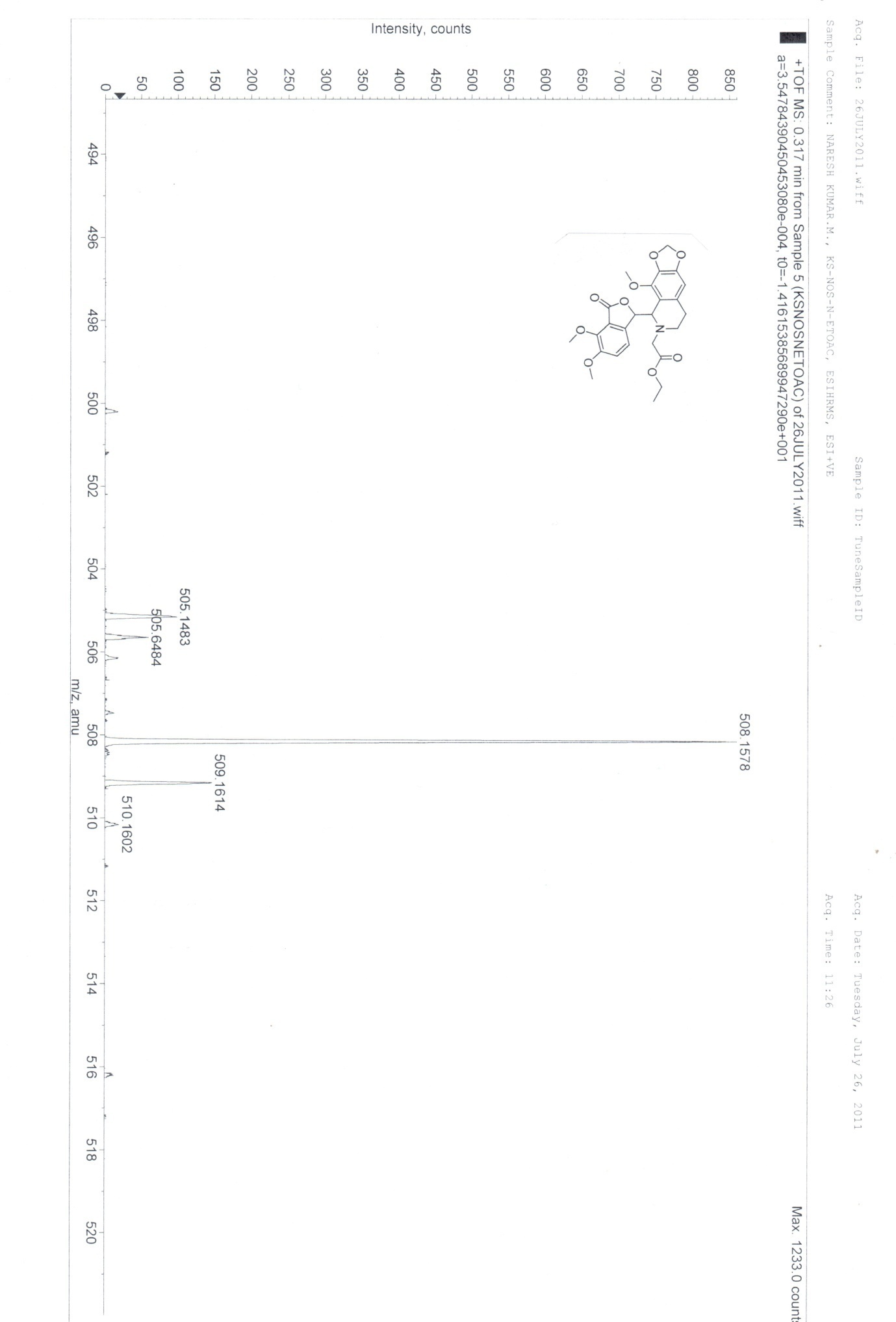 |

| **HRMS spectra of 6i.** |
| --- |
| 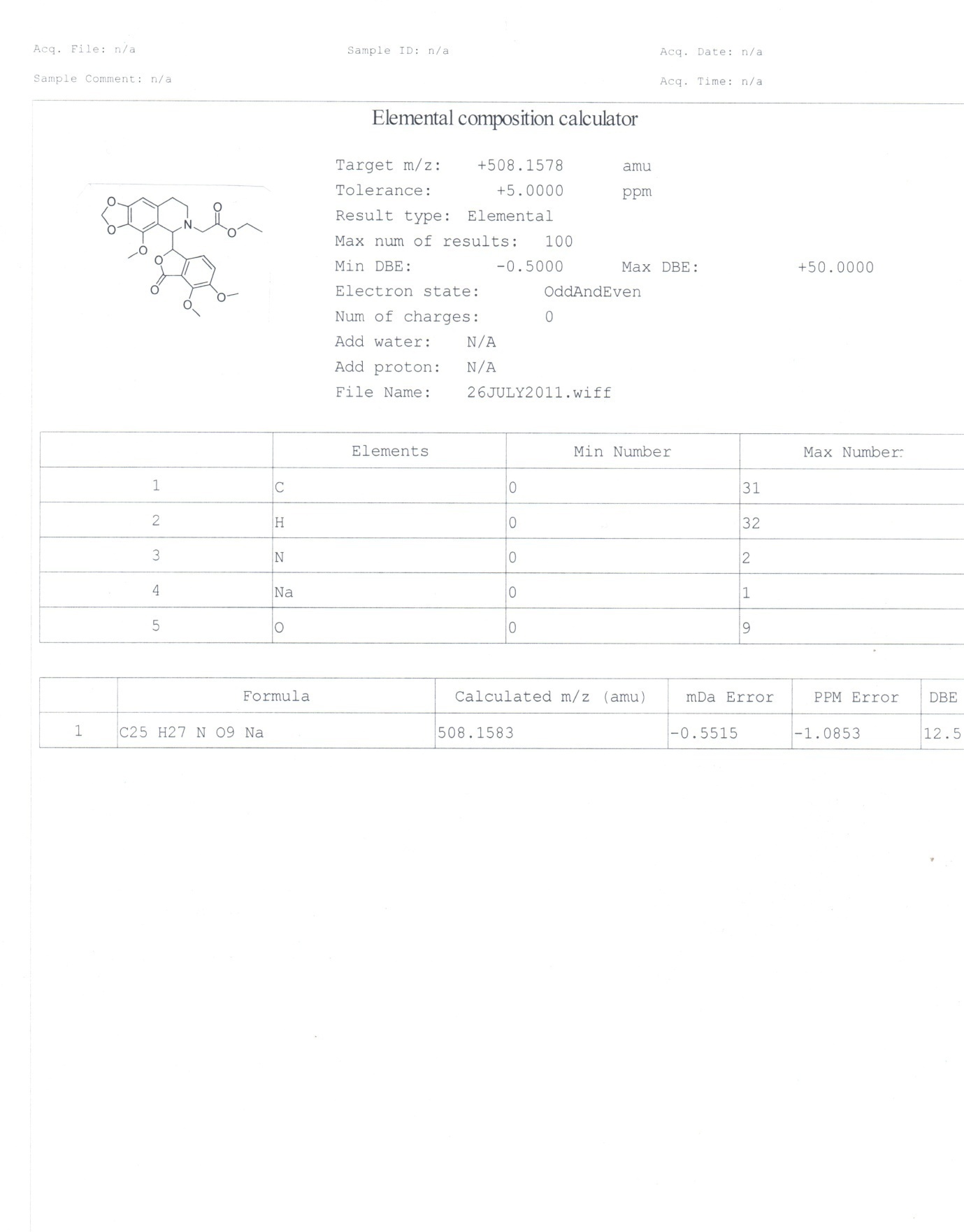 |

| **^1^H NMR spectra of 6j.** |
| --- |
| 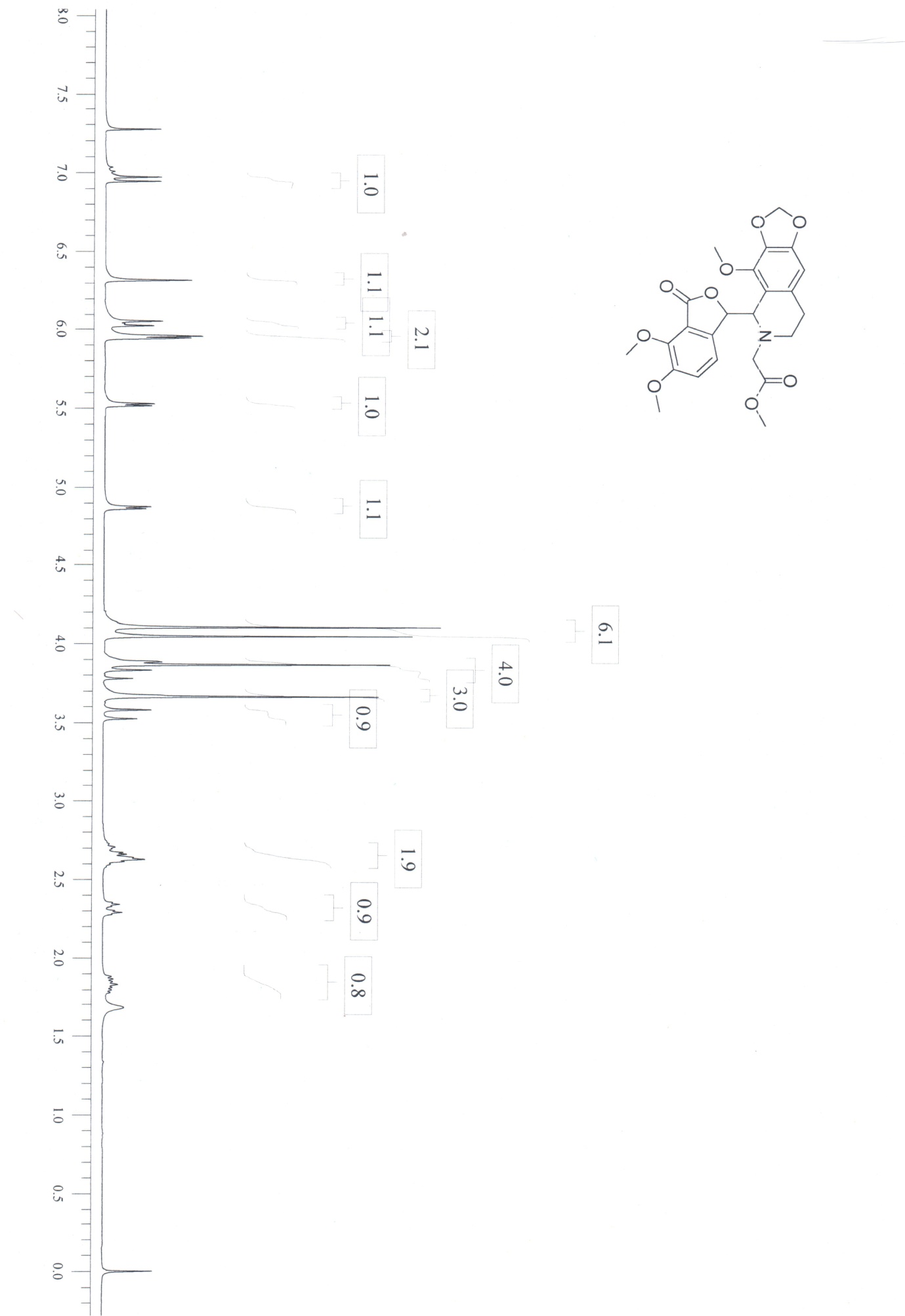 |

| **^13^C spectra of 6j.** |
| --- |
| 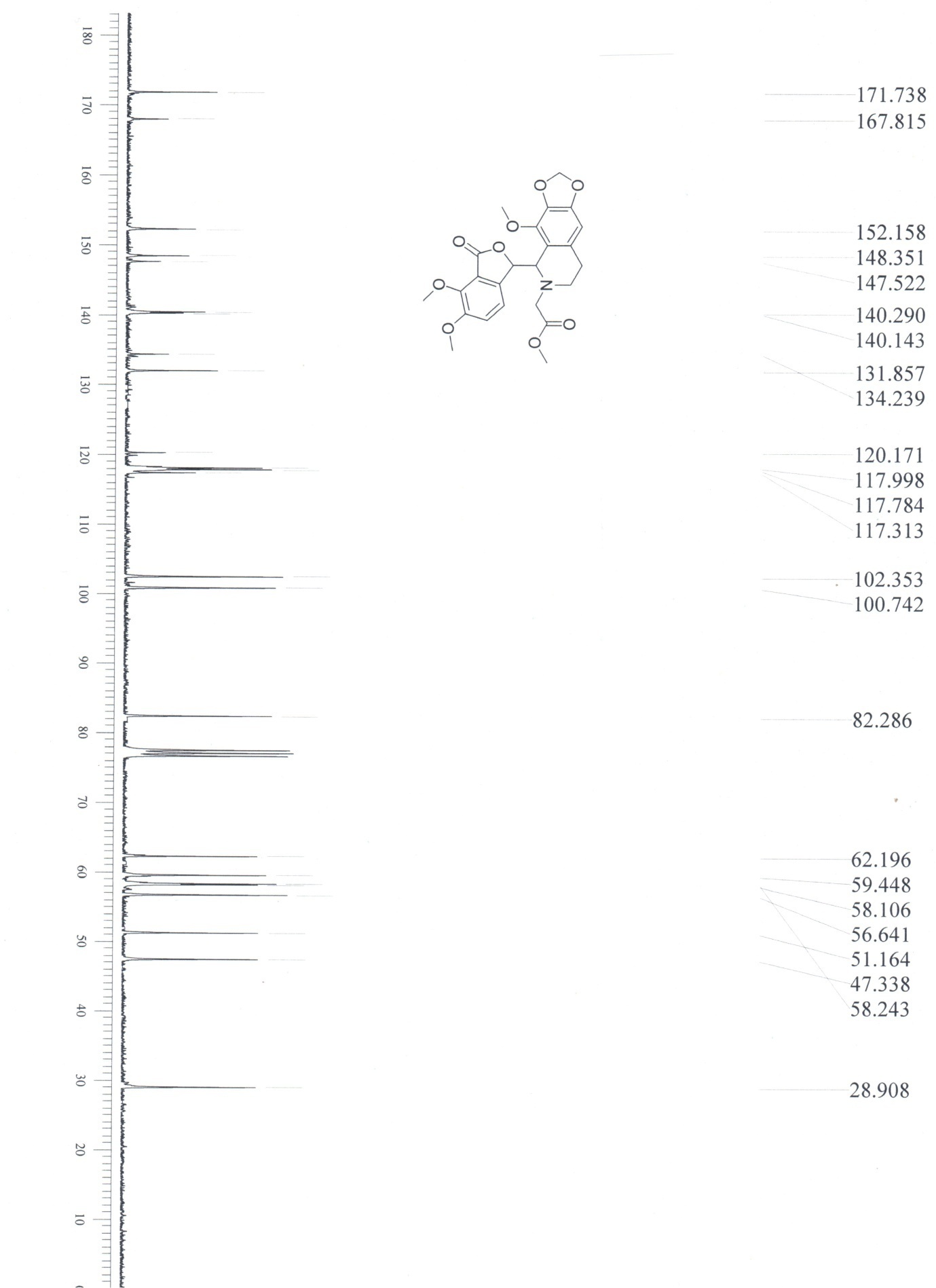 |

| **ESI spectra of 6j.** |
| --- |
| 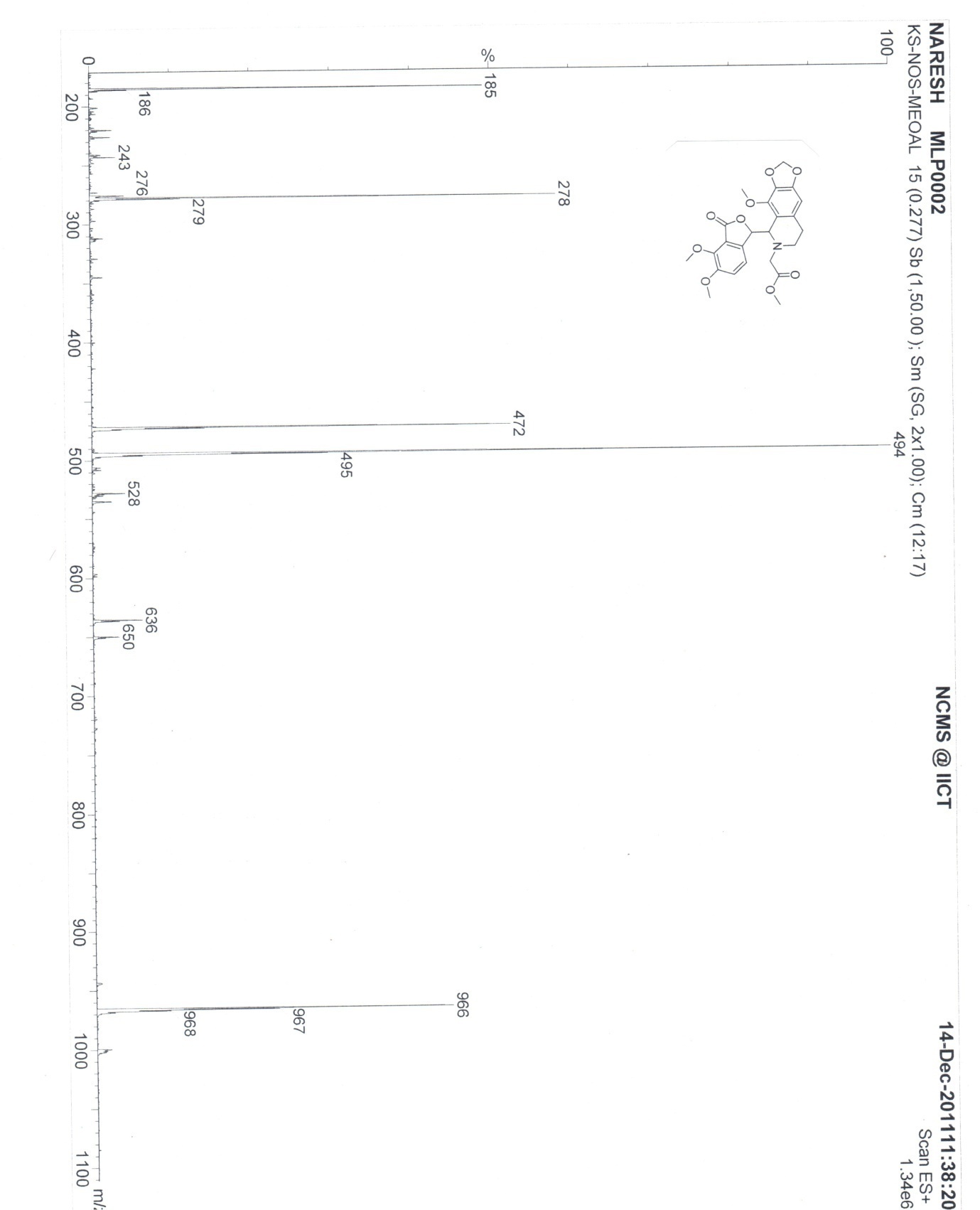 |
