# Supplementary material for: Rational Design, Synthesis, and Biological Evaluation of Third Generation α-Noscapine Analogues as Potent Tubulin Binding Anti-Cancer Agents
Source: PLoS One. 2013 Oct 21;8(10):e77970. doi: 10.1371/journal.pone.0077970 (PMC3804772; doi:10.1371/journal.pone.0077970)
Supplement: Table S2 — 1H NMR, 13C NMR and ESI / HRMS spectra of noscapinoids 5a and 6a-j. (DOCX) [file pone.0077970.s003.docx]

**Table S2:**  Binding of new noscapinoids **5a, 6c, 6f, 6i-j** to tubulin as measured by fluorescence quenching of tubulin. (A) quenching of tubulin fluorescence emission by noscapinoids **5a, 6c, 6f, 6i-j** in a concentration-dependent manner (control 0 μM (♦), 50 μM (■), 100 μM (▲) and 200 μM (x)). (B) double-reciprocal plot showing a dissociation constant (K_d_) of compounds (5a & 6a-j) binding to tubulin.

| Noscapinoids | A | B |
| --- | --- | --- |
| **5a**  (K_D_: 68 ± 0.7 μM) |  |  |
| **6c**  (K_D_: 91 ± 8 μM) |  |  |
| **6f**  (K_D_: 38 ± 4 μM) |  |  |
| **6i**  (K_D_: 79 ± 8 μM) | 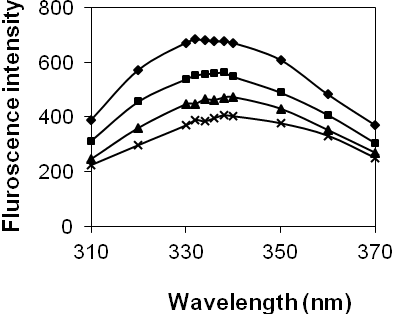 | 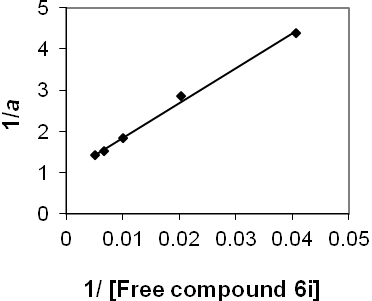 |
| **6j**  (K_D_: 228 ± 10 μM) | 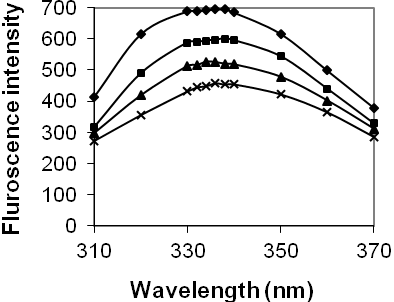 | 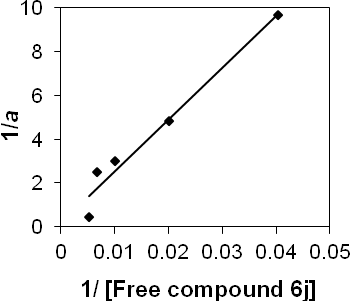 |
